# Sequence-based coevolutionary prediction of species-specific interactomes

**DOI:** 10.1371/journal.pcbi.1014839

**Published:** 2026-11-06

**Authors:** Miguel Fernandez-Martin, Camila Pontes, Victoria Ruiz-Serra, Alfonso Valencia

**Affiliations:** 1 Computational Biology Group, Life Sciences Department, Barcelona Supercomputing Center, Barcelona, Spain; 2 Evolutionary Systems Biophysics Group, Life Sciences Department, Barcelona Supercomputing Center, Barcelona, Spain; 3 Universitat Pompeu Fabra (UPF), Barcelona, Spain; 4 European Commission, Joint Research Centre (JRC), Geel, Belgium; 5 ICREA, Barcelona, Spain; Robert Koch Institute: Robert Koch Institut, GERMANY

## Abstract

Evolutionarily conserved protein-protein interactions (PPIs) reveal fundamental biological processes. However, predicting protein function solely from these interactions provides an incomplete picture of biological systems. Computational methods for predicting PPIs often struggle due to limited functional annotations in databases, making it difficult to fully understand unique biological systems. This study introduces ContextMirror2.0 (CM2.0), a coevolution-based method designed to address these limitations. Coevolutionary approaches, unlike supervised machine learning methods, do not rely on labelled datasets, proving valuable in addressing the scarcity of data for species-specific PPIs. Around 40% of CM2.0’s top-1000 predicted PPIs for *E. coli, S. enterica*, and *S. aureus* are supported by experimental PPI databases. However, comparative analysis of the resulting functional communities further revealed both highly conserved interaction patterns and species-specific interactions. CM2.0 leverages coevolutionary information at the phylogenetic level to explore protein interaction dynamics and understand the functional consequences of species-specific variations. This information can inform further studies on the emergence of novel functions, adaptation to specific environments, and the development of targeted therapies.

## Author summary

Predicting how proteins interact is essential for understanding how cells work, yet most protein–protein interactions remain unknown, particularly in bacteria and other less-studied organisms. ContextMirror 2.0 is an improved version of our unsupervised computational method that detects functional relationships between proteins by comparing how their evolutionary histories change across thousands of species. Unlike machine-learning and structure-based predictors, ContextMirror 2.0 does not require training data or detailed structural information, making it suitable for analyzing complete proteomes. We applied the method to three bacterial species and found that it accurately recovers many known interactions and identifies new, species-specific protein communities that suggest potential biological functions. We also tested its performance using several benchmarking strategies, including both balanced and realistically imbalanced datasets, and showed that it consistently enriches true interactions above random expectation. Because it is scalable and interpretable, ContextMirror 2.0 can help researchers explore poorly characterized proteomes and guide future experimental studies of protein function and interaction.

## 1 Introduction

Understanding protein interactions is crucial for deciphering cellular mechanisms, disease pathways, and evolutionary processes, yet significant knowledge gaps remain that limit our comprehensive understanding of biological systems. These gaps are especially concerning for species-specific interactions and those in non-model organisms with poorly annotated proteomes. In this context, computational methods offer valuable alternatives to predict potential interactions and prioritize experimental validation efforts, ultimately accelerating our understanding of protein networks across diverse biological systems [1].

Traditional computational approaches for predicting protein-protein interactions (PPIs) encompass sequence-based methods such as homology detection, domain-domain interaction mapping, and phylogenetic profiling [2,3], alongside structure-based approaches including molecular docking and template-based modeling [4]. Network-based methods utilize topological features and graph theoretical approaches to identify potential interactions [5]. Recent developments have explored advanced machine learning architectures, with deep learning models incorporating multiple data modalities including sequence embeddings, structural features, and evolutionary information. Notable advances include Cong et al. (2019) [6], which introduced a large-scale coevolution-based framework for proteome-wide PPI prediction; Wu et al. (2023) [7] (DL‑PPI), which refined these approaches through a novel Inception‑based feature extraction module and Graph Neural Network‑driven relational reasoning network (FRN) to better integrate diverse biological features and improve prediction on unfamiliar datasets; and the recent Zhang et al. (2024) [8], which reviewed and benchmarked deep-learning approaches, emphasizing the challenges of scaling from balanced datasets (1:1) to realistic proteome-wide unbalanced scenarios (e.g., 1:1000). In addition, network-based approaches such as PPISB [9], which leverage mixed-membership stochastic blockmodels to detect community structures in PPI networks, have also been proposed; the main strength lies in identifying latent community structures in PPI networks and predicting new interactions.

Despite the advances, current computational methods for predicting complete interactomes are limited. Many machine learning (ML)-based approaches require extensive labelled data, hindering reliable extrapolation beyond a few reference proteomes [10–12]. For example, evaluating predicted PPI networks is challenging, even for small bacterial proteomes, given that a vast number of potential PPIs lack experimental evidence [13,14]. Furthermore, the explainability of ML models, particularly deep neural networks, is often limited, and most of the existing PPI prediction methods are either supervised [15–17] or rely on structural information and do not work for large scale prediction [18–20]. Although recent developments have improved the scalability of structure-based approaches [21], systematic proteome-wide prediction remains computationally demanding and challenging to benchmark. In the case of network-based approaches such as PPISB, additional limitations include poor scalability to very large datasets and reduced recall in highly complex networks.

To address these challenges, we have developed ContextMirror2.0 (CM2.0), a coevolution-based method built upon our previous work [22]. CM2.0 is a new implementation of the ContextMirror framework based on a redesigned algorithm that enables efficient proteome-scale prediction while preserving the original concept of exploiting phylogenetic context to capture coevolutionary signals. We believe its core idea remains valuable, as it relies solely on evolutionary information and does not require training data or supervision, providing a complementary perspective to existing PPI prediction methods. The workflow first filters protein pairs according to the number of matching species represented in their paired alignments, excluding pairs lacking sufficient evolutionary information for reliable coevolutionary analysis, and then ranks the retained pairs according to the strength of their coevolutionary signal. For the retained pairs CM2.0 measures coevolution by comparing sequence distance patterns among orthologous protein families across species. In other words, it leverages the coevolutionary network of an organism, defined as the network containing all of the pairwise phylogenetic tree similarities, to quantify the coevolutionary signal between two proteins. The central idea is that proteins involved in the same biological processes tend to exhibit similar patterns of evolution with other proteins. CM2.0 compares these evolutionary profiles to measure their coevolutionary signal, which is then used to predict functional associations. Unlike existing approaches, CM2.0 is entirely unsupervised and sequence-based, making it fundamentally different from (ML)-based methods. Additionally, it provides a confidence score of the predictions, providing a reliable guide for future experimental and computational research. Compared with the original ContextMirror framework, CM2.0 introduces several methodological advances, including a redesigned algorithm that enables efficient proteome-scale analyses, an automated workflow for ortholog retrieval and paired MSA construction, revised statistical procedures for interaction scoring, and an unsupervised community-detection framework for network analysis, together with numerous technical improvements to modernize the computational implementation.

We validated CM2.0 predictions using a combined dataset of positive interactions of three bacterial proteomes *(E. coli, S. enterica* and *S. aureus*), achieving 40% precision for the 1000 predictions. This accuracy, while moderate, is clearly significant and sufficient for identifying sets of specific PPIs, especially when reinforced by community search of combined multispecies PPI networks as we observed a 3–8% increase in precision for intra-community edges.

Nevertheless, to place CM2.0 in the context of state-of-the-art PPI prediction methods, we benchmarked it against representative AI-based approaches, namely the sequence-based D-Script [23] and the structure-based RoseTTAFold2-PPI [24], as well as the coevolution-based method of Cong et al. (2019) [6].

Additionally, by comparing interaction networks across species, we identified species-specific interactions worthy of further investigation, including examples, such as proteins from the phn gene community in *E. coli* and a flavoprotein complex in *S. enterica*.

In summary, CM2.0 offers a valuable approach for predicting PPIs, identifying both conserved interaction clusters while also uncovering species-specific interactions with potential biological significance. This information can contribute to the functional annotation of poorly characterised proteins and the identification of new complexes in understudied organisms, as exemplified by the case of *S. aureus* in this study.

## 2 Results

### 2.1 ContextMirror2.0 accurately predicts pairwise PPIs

We applied ContextMirror2.0 (CM2.0), a new high-throughput version of the ContextMirror method [22] (see Methods), to predict the interactomes of *E. coli*, *S. enterica*, and *S. aureus*, based on species-specific alignments using as heads the proteins of each one of the three organisms. CM2.0 first applies a coevolutionary filtering stage to remove protein pairs lacking sufficient evolutionary support and subsequently assigns an interaction score (IntScore) to the retained candidates. This score incorporates the phylogenetic correlation between protein families, corrected for background noise, and the average influence of third proteins’ phylogenetic profiles, that is, proteins that influence the correlation between two given protein interactors (see Methods). Higher scores reflect stronger coevolutionary signals, which is interpreted as an indicator of the quality of the predictions. There is also a positive relationship between phylogenetic correlation and the number of influencing third proteins (S1 Fig), suggesting that more strongly correlated profiles tend to be influenced by many third proteins. This relation is consistent with the modular organization of protein interaction networks in which proteins involved in related biological functions are connected through multiple direct and indirect relationships.

However, the integration of these components into CM2.0’s interaction score could lead to potential biases. Specifically, when a PPI is influenced by only a few third proteins, the interaction score can be inflated by a single strong partial correlation. In contrast, when many third proteins are involved, the interaction score is averaged across all of them, potentially lowering the score but providing a more comprehensive coevolutionary context, especially for PPIs influenced by a small number of third proteins. To reduce the influence of isolated high partial correlations (influences), we introduced a penalization strategy to lower the IntScore of PPIs influenced by a small number of third proteins (<50). This cutoff was selected because the relationship between phylogenetic correlation and the number of influencing proteins levels off below this value, and statistical analyses further demonstrated a significantly higher concentration of false positives in this range (see Methods and S1 Fig).

CM2.0 interaction score (IntScore) is a number between 0 and 2. The selection of an appropriate IntScore threshold is crucial when analysing CM2.0 predictions, as it serves as a confidence indicator. The ideal threshold will depend on the application. A low threshold will include a high number of PPIs of both high- and low-confidence, while a high threshold will include a lower number of high-confidence PPIs. Here, we considered three IntScore thresholds: 1.6, 1.7, and 1.8, adjusting them as needed to address specific research questions. These thresholds should be interpreted as representative confidence levels that illustrate the trade-off between coverage and reliability. In the absence of benchmark interaction data, users may select more stringent or permissive portions of the ranked interactome depending on whether their goal is high-confidence prediction or broader exploratory analysis.

Fig 1A demonstrates high precision for top-ranked interactions (approximately 40% for the top 1000 predictions), decreasing to around 20% with more predictions across all species. These precision values are reasonable considering the vast number of potential interactions (approximately 9 million for *E. coli)* and potential incompleteness of the ground truth data (precision values for various interaction score thresholds are given in S1 Table). The precision of our highest-ranked interactions significantly surpasses baseline rates, calculated as the fraction of validated interactions among all possible protein pairs after ortholog filtering (4.9% for *E. coli*, 4% for *S. enterica*, and 3.9% for *S. aureus*), yielding a substantial improvement over random chance (≈8.5 fold enrichment). This indicates that CM2.0 effectively ranks known interactions while also identifying novel potential interactions.

**Fig 1:**
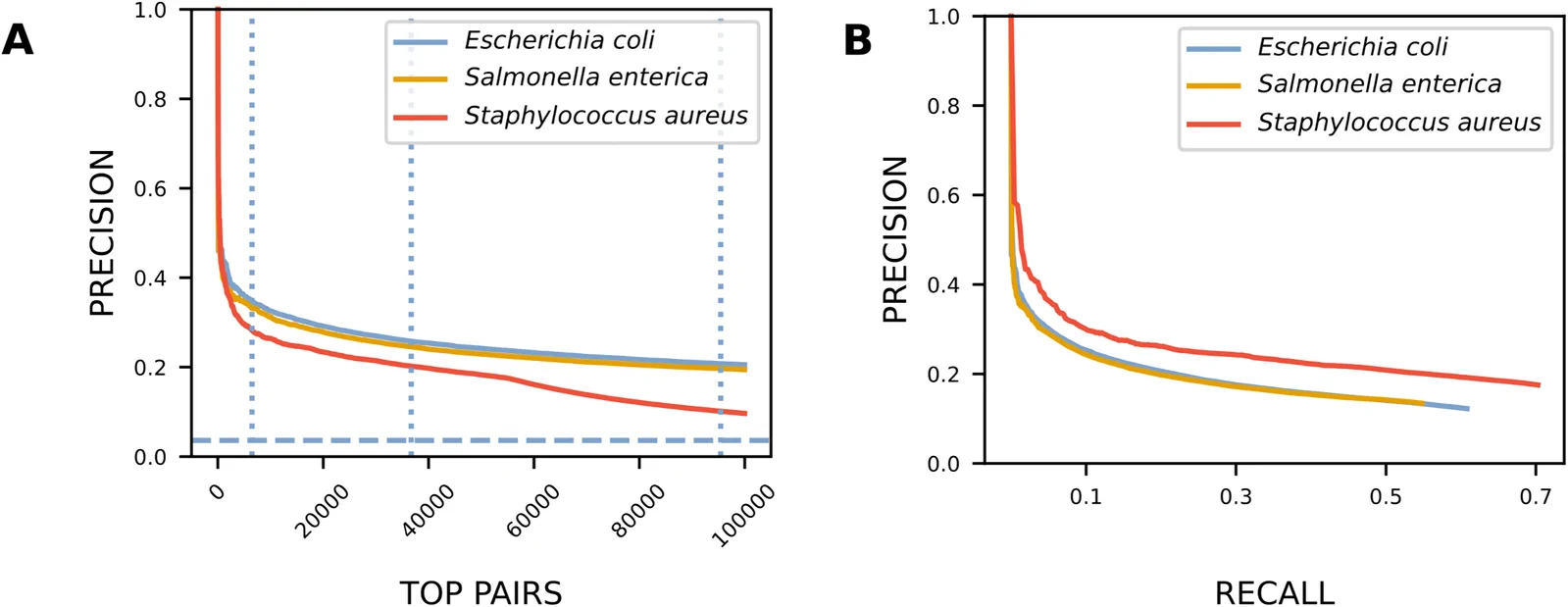
Pairwise accuracy of ContextMirror2.0 predictions. **A)** Precision results for different sets of top-ranked predicted interactions for *E. coli* (blue), *S. enterica* (orange) and *S. aureus* (red). The dashed line corresponds to the precision values that would be expected by chance for *E. coli* (4.9%), *S. enterica* (4%) and *S. aureus* (3.9%). The dotted lines indicate the precision corresponding to different interaction scores of 1.8, 1.7 and 1.6 in *E. coli*. **B)** Precision-recall curve for all species.

Next, we examined the precision-recall trade-off (Fig 1B). For *E. coli*, CM2.0 predicted 505,294 PPIs, compared to 312,961 interactions from the STRING database. Because CM2.0 only scores protein pairs that pass the initial coevolutionary filters, approximately 20% of the ground truth set was recovered with a precision of roughly 20%, comparable to the performance reported by Cong et al. (2019), using residue-level coevolution (DCA) in *E. coli* [6], although this approach was computationally more intensive.

We investigated the relationship between interaction scores and paired multiple sequence alignment (MSA) depth, quantified as the effective depth (Neff) of the paired MSA. A moderate correlation was observed (0.47 for *E. coli*, 0.48 for *S. enterica*, and 0.3 for *S. aureus*, all with p-values < 0.00001), with alignment depth explaining 13% of the variance in interaction scores for *E. coli* and *S. enterica* (16% for *S. aureus*) (S2 Fig). This indicates that while alignment depth contributes to interaction scores, it is not the only determining factor.

Neff reflects the effective diversity of the paired MSA rather than the total number of matching species contributing to it. We therefore investigated whether the predictive power of IntScore reflected two well-established proxies of bacterial protein interactions: the number of matching species used to construct the paired MSA and genomic proximity. Protein pairs represented across a larger number of matching species are more likely to correspond to experimentally validated interactions. Since CM2.0 explicitly exploits the coevolutionary information contained in these paired alignments, some dependence of IntScore on the number of matching species is expected. Likewise, proteins encoded by genes in close genomic proximity are frequently co-transcribed and functionally related [25]. To determine whether IntScore provides predictive information beyond these variables, protein pairs were stratified according to the number of matching species or intergenic distance. In both analyses, validated interactions consistently received higher IntScores than unsupported interactions within every bin (S3 Fig). For the matching-species analysis, the separation became more pronounced as the number of matching species increased, consistent with the greater amount of coevolutionary information available in deeper paired alignments. Logistic regression further confirmed that IntScore remained a significant predictor of interaction status after controlling for either the number of matching species or intergenic distance (S2 Table).

Finally, we evaluated operon organization, one of the strongest genomic determinants of bacterial protein interactions [26]. As expected, proteins encoded within the same operon generally received high IntScores and are experimentally validated (S3C Fig). However, high-confidence predictions were not restricted to intra-operon pairs. At IntScore ≥1.8, CM2.0 recovered 53 validated same-operon interactions together with 2,202 validated interactions involving proteins not annotated in the same operon (S3 Table). Consequently, the ranked interactome is overwhelmingly composed of inter-operon predictions, and the changes in precision observed across IntScore thresholds are primarily driven by these interactions. These results indicate that although CM2.0 captures operon organization, it also identifies large numbers of experimentally supported interactions connecting different transcriptional units.

Collectively, these analyses indicate that IntScore captures several biological features associated with protein interactions, including evolutionary information, genomic proximity and operon organization, while retaining significant discriminative power between supported and unsupported interactions.

To further characterize a subset of CM2.0 predictions, we modelled 60 randomly selected PPIs (10 high- and 10 low-IntScore pairs from each organism) using AlphaFold Multimer [27,28] and evaluated the resulting complexes using the pDockQ2 score [29] (see Methods). Three high-IntScore PPIs yielded acceptable structural models (pDockQ2 > 0.23), whereas only one of the low-scoring pairs did (S4 Fig). These results suggest that high IntScore interactions are more likely to be compatible with structural modelling, although many biologically relevant associations identified by CM2.0 are likely not mediated by stable physical complexes and therefore remain challenging for current structure-based predictors.

To compare CM2.0 with current state-of-the-art PPI prediction methods, we benchmarked it against RoseTTAFold2-PPI and D-Script using four experimentally characterized bacterial protein complexes: the Nuo and ATP synthase complexes from *E. coli* and the Dpp and Liv transporters from *S. enterica*. For each complex, all possible intra-complex protein pairs were generated together with pairs between complex members and unrelated cytosolic proteins. These benchmark datasets contained both experimentally validated and unsupported interactions, allowing us to evaluate the ability of each method to distinguish supported from unsupported PPIs.

Since RoseTTAFold2-PPI and D-Script assign scores to every queried protein pair, whereas CM2.0 only evaluates pairs that pass its initial coevolutionary filters, we first restricted the comparison to the subset of protein pairs scored by CM2.0. Under these conditions, IntScore consistently achieved the highest discrimination between experimentally validated and unsupported interactions across the four benchmark systems. For example, in the Nuo benchmark, IntScore achieved an AUC of 0.94, compared with 0.75 for D-Script and 0.52 for RoseTTAFold2-PPI (Fig 2). Similar behaviour was observed for the ATP, Dpp and Liv benchmarks (S5 Fig and S4 Table). These results indicate that, when evaluated on the same candidate interaction space, IntScore provides stronger discrimination between supported and unsupported interactions than either RoseTTAFold2-PPI or D-Script.

**Fig 2:**
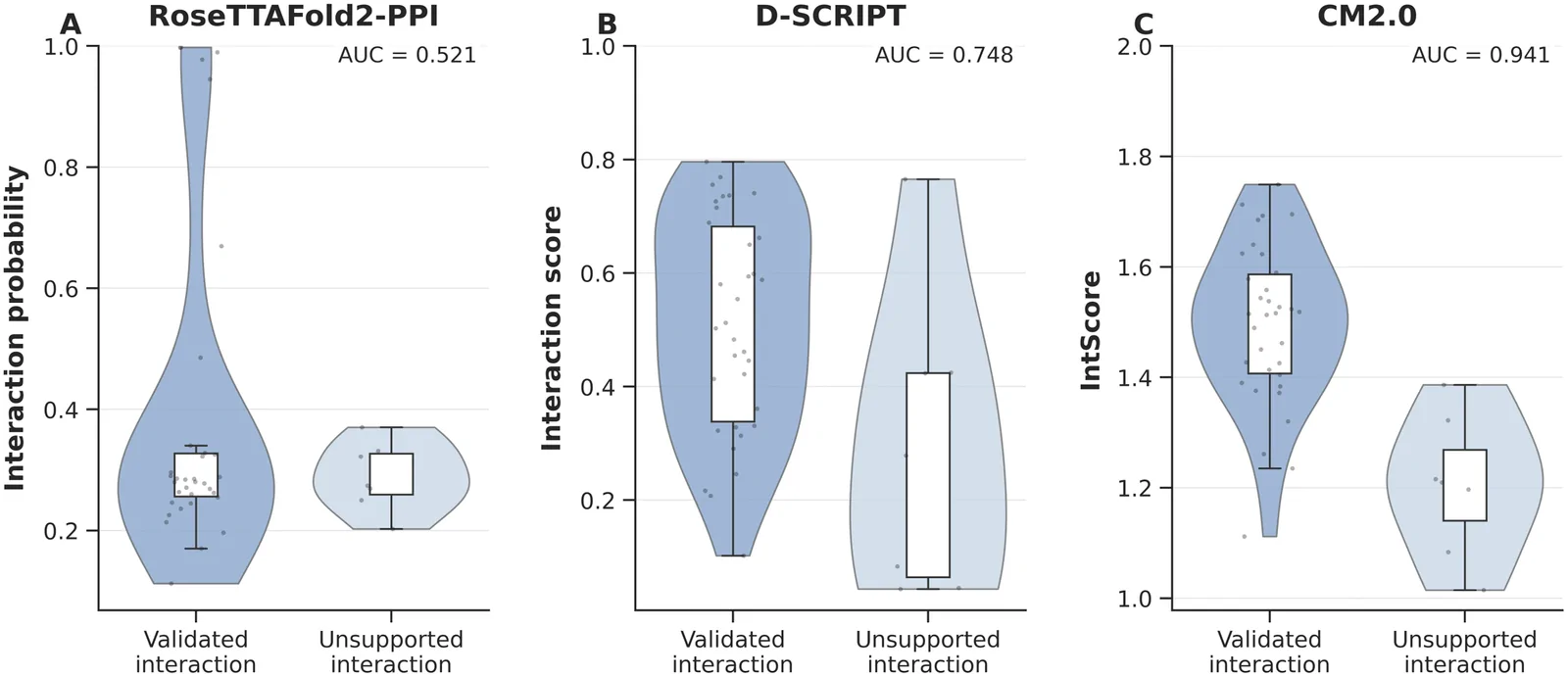
Score distributions for experimentally validated and unsupported protein pairs in the Nuo benchmark. Violin plots compare RoseTTAFold2-PPI **(A)**, D-Script (B) and CM2.0 IntScore (C) for the subset of protein pairs evaluated by CM2.0 after coevolutionary filtering.

Because experimentally validated interactions may represent only a subset of biologically relevant associations, we repeated the analysis using complex membership as an independent interaction proxy. The same qualitative conclusions were obtained (S6 Fig and S4 Table).

However, this comparison only evaluates the ranking stage of CM2.0. Unlike RoseTTAFold2-PPI and D-Script, CM2.0 first applies an unsupervised coevolutionary filter that removes protein pairs lacking sufficient evolutionary information before assigning an IntScore. To compare the complete workflows, we assigned IntScore = 0 to pairs initially discarded by the filter as a technical strategy to enable end-to-end evaluation. Under these conditions, CM2.0 continued to outperform both methods in the ATP and Dpp benchmarks, whereas performance decreased in the more aggressively filtered Nuo and Liv systems (S5 Table).

To understand these differences, we quantified the behaviour of the coevolutionary filtering step. Across all four benchmark systems, experimentally supported interactions were consistently more likely to survive the filter than unsupported interactions (Fisher OR = 4.1–∞; S7 Fig and S6 Table). Thus, the reduction in end-to-end performance observed in some systems does not arise because CM2.0 indiscriminately removes interactions, but because the filter becomes increasingly conservative when evolutionary information is limited. In Nuo and Liv this conservatism sacrifices a larger fraction of true interactions, whereas in ATP and Dpp most supported interactions survive the filtering stage.

These benchmarks complement our previous validation using the same curated datasets as Cong et al. (2019) and the BioGRID physical interaction benchmarks. On the PDB benchmark CM2.0 reached ≈3% precision, corresponding to a ten-fold enrichment over random (~0.2% baseline), a typical value for this highly imbalanced dataset where positives represent only a small fraction of all possible pairs; and ≈14-fold enrichment on functional datasets (Y2H, EcoCyc, APMS). Similarly, on BioGRID physical interaction dataset, CM2.0 achieved ≈0.89 precision (AUPRC = 0.77) in the balanced 1:1 setup and ≈ 0.009 precision in a strongly imbalanced 1:400 scenario, representative of realistic proteome-wide class imbalance, while still showing a ≈ 3.7 × enrichment over random. These complementary benchmarks follow recent recommendations to evaluate PPI predictors under both balanced and realistically imbalanced conditions [8].

Collectively, these analyses demonstrate that CM2.0 combines biologically meaningful interaction ranking within the candidate space defined by the initial coevolutionary filtering stage. Across multiple validation settings, IntScore consistently discriminated experimentally supported interactions and performed competitively with current state-of-the-art PPI prediction methods. In the following sections, we will investigate how the pairwise PPIs predicted for individual organisms can be leveraged to identify common and species-specific protein functional communities.

### 2.2 Species-specific functional communities can be identified within a multispecies PPI network

In this section, we evaluated CM2.0’s predictive accuracy in the context of conserved and species-specific protein-protein interactions (PPIs). To achieve this, we identified common and distinct PPIs by mapping homologous sequences across species through reciprocal best-hit homolog relationships (see Methods). In this context, we define species-specific interactions as those detected in one species but absent from the corresponding network of another species included in the analysis. For instance, the examples below highlight interactions identified in *S. aureus* that are not detected in either *E. coli* or *S. enterica*. These comparisons demonstrate CM2.0’s capacity to distinguish biologically relevant differences in interaction networks across species.

As expected, the Gram-negative bacteria *E. coli* and *S. enterica* shared a high proportion of pairwise interactions (70.0%), whereas the more distantly related Gram-positive *S. aureus* shared fewer interactions with both (40.7% and 48.3%, respectively) (Fig 3), a finding consistent with their phylogenetic distance and functional differences [30]. High-confidence predictions (i.e., higher interaction scores) showed greater conservation across species (S8 Fig), suggesting that conserved PPIs tend to involve ubiquitous bacterial proteins. Conversely, species-specific PPIs, being less well-characterised, rely on less diverse alignments, potentially leading to weaker coevolutionary signals. This reflects a precision-recall trade-off when predicting species-specific interactions.

**Fig 3:**
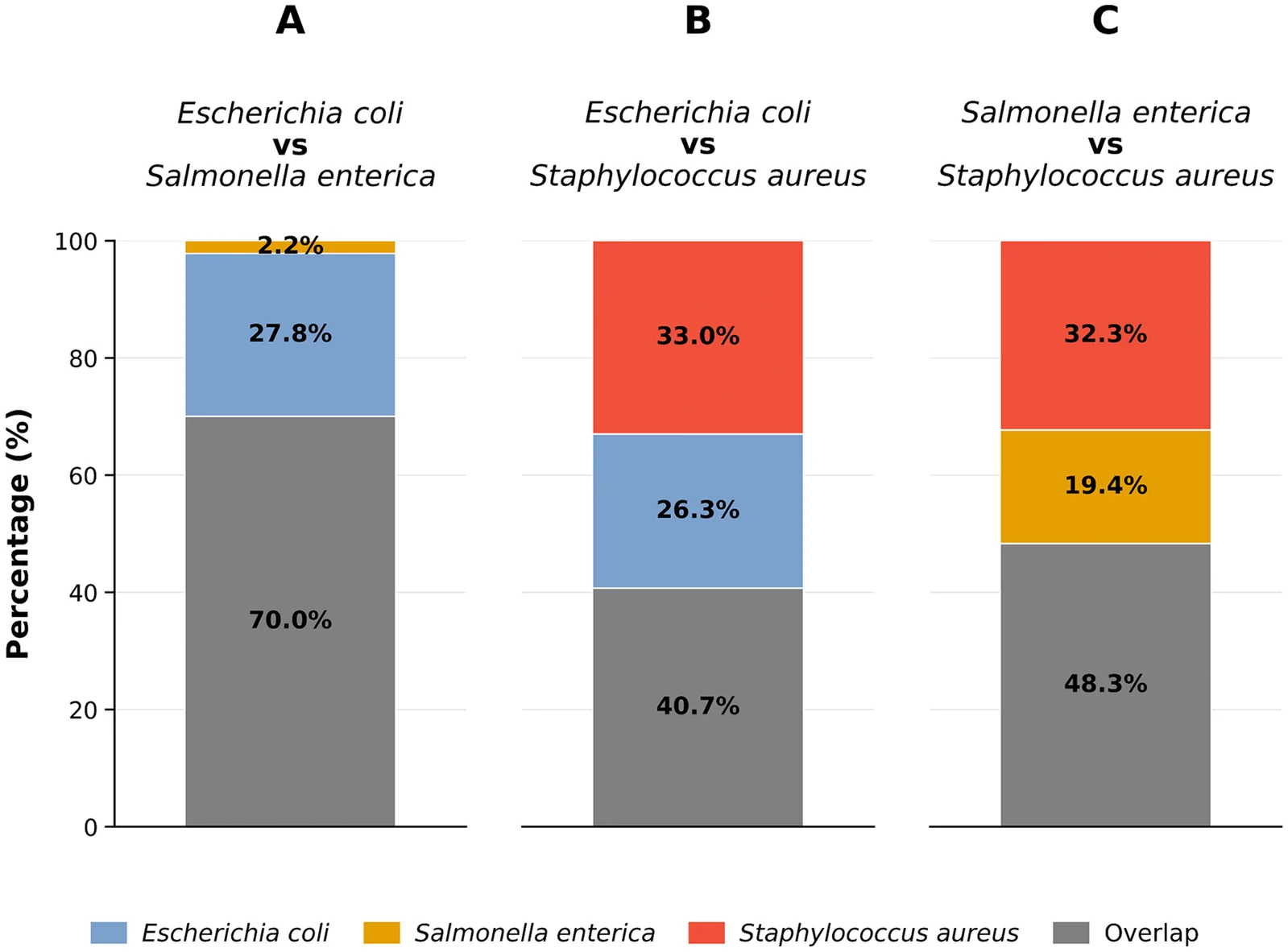
Percentage of shared PPIs between species. Panels A, B and C show the PPI overlap between *E. coli* and *S. enterica* (n = 467505), *E. coli* and *S. aureus* (n = 40403), and *S. enterica* and *S. aureus* (n = 53580), respectively. Shared interactions between species are shown in gray, *E. coli* unique interactions are shown in blue, *S. enterica* unique interactions are shown in orange, and *S. aureus* unique interactions are shown in red.

Next, we constructed PPI networks for the two better-characterised species, *E. coli* and *S. enterica,* with edges weighted according to their interaction score (IntScore). We generated a multispecies PPI network by combining these two by matching the homologous proteins (nodes) that were identified in the previous step. This was done considering different IntScore thresholds, i.e., IntScore > 1.8, IntScore > 1.7 and IntScore > 1.6. For the strictest threshold (IntScore > 1.8) for both species, precision was around 35%, and the combined network contained 531 nodes and 1492 edges (see S7 Table for thresholds 1.6 and 1.7).

To identify functional associations between groups of proteins, we predicted communities in the combined network using the Louvain algorithm with different initial random states (see Methods). S9 Fig shows the communities obtained on the run with highest overall intra-community precision, while the results presented in the following are averages from multiple runs. Fig 4 shows the average percentages of true positives (TPs) and false positives (FPs) within communities for the combined networks computed with different IntScore thresholds. The results show that as the IntScore threshold increases, the percentage of TPs rises and FPs decreases in both species, as expected. The proportion of predicted interactions that are TPs in one species and FPs in another remains low and constant across IntScore thresholds.

**Fig 4:**
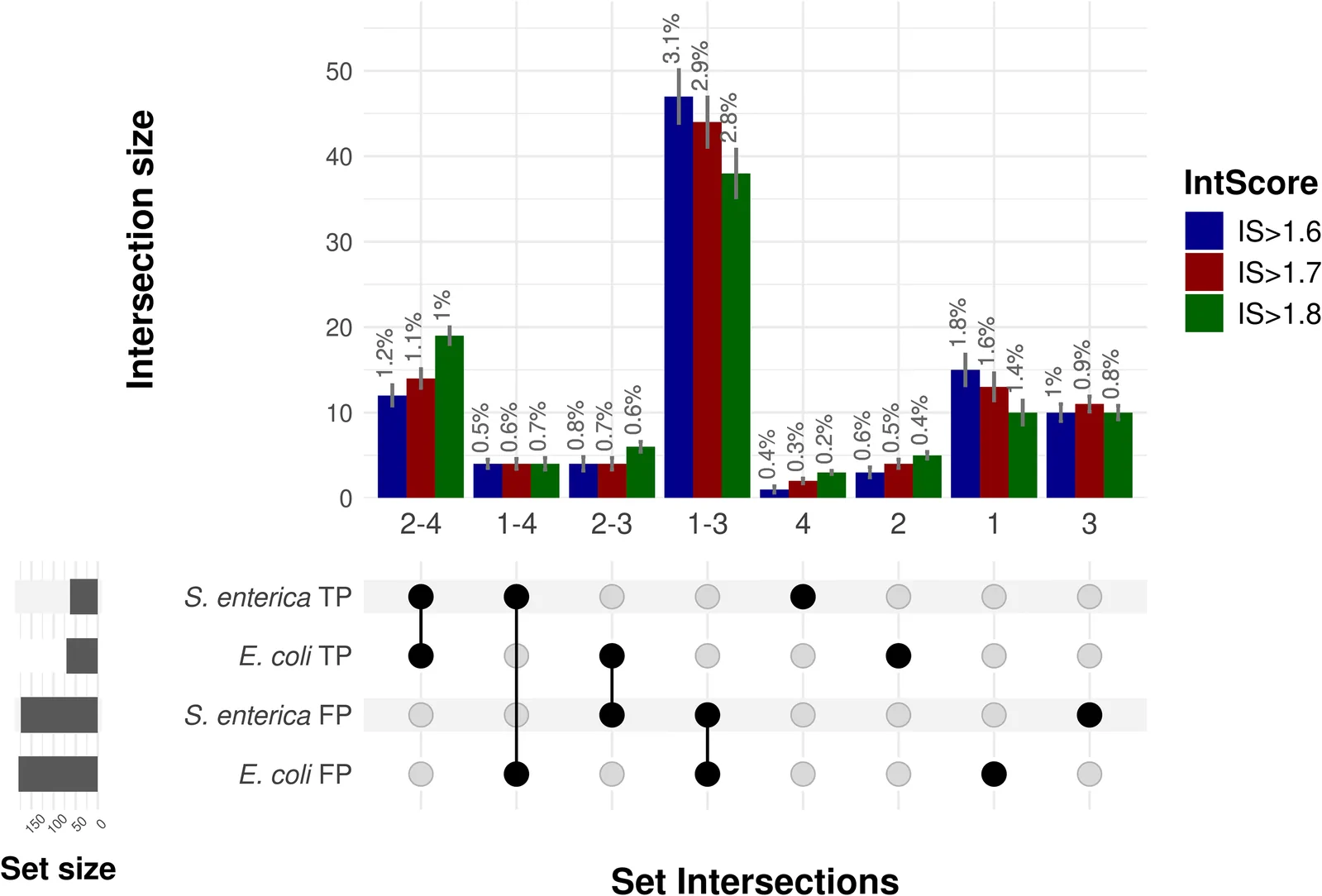
Average number of intra-community edges of different types in the combined PPI network of *E. coli* and *S. enterica*, as inferred by CM2.0. The first four sets (2-4, 1-4, 2-3 and 1-3) represent common PPIs while the last four sets (4, 2, 1 and 3) represent species-specific PPIs. The predicted PPIs are separated according to their validation status in the groundtruth PPI sets as a known interaction or true positive (TP), or as an unknown interaction or false positive (FP). The average number of intra-communities edges belonging to each set is shown for different interaction score (IntScore) thresholds: IntScore > 1.6 (blue), IntScore > 1.7 (red) and IntScore > 1.8 (green). Communities were determined using the Louvain algorithm with different initial random states.

This analysis identified three sets of interesting PPIs: (1) PPIs reported in one species but not in the other (sets 1–4 and 2–3 in Fig 4), (2) unreported PPIs identified in both species (set 1–3 in Fig 4), and (3) both reported and unreported species-specific PPIs (sets 4, 2, 1 and 3 in Fig 4). Specifically, we identified around 104 potentially novel PPIs in *S. enterica* (reported in *E. coli*), and 78 potentially novel PPIs in *E. coli* (confirmed in *S. enterica*). These cases may represent missing or incomplete annotations in one of the two species. In addition, we identified 610 high-confidence PPIs shared by both species that are not currently annotated in either ground-truth dataset. Although these interactions cannot be considered validated on the basis of the present analysis alone, their independent prediction in both species makes them plausible candidates for previously uncharacterized conserved associations, which could be further assessed using complementary sources of evidence such as transcriptomic co-expression or other functional association data. Finally, we identified 249 and 226 high-confidence species-specific PPIs in *E. coli* and *S. enterica*, respectively, of which 163 and 165 were not annotated in the corresponding ground-truth datasets. Here, species-specific is used in an operational sense to denote interactions predicted in one species but not in the other species under comparison; these unannotated interactions are currently not reported in the reference datasets but constitute high-confidence CM2.0 predictions, some of which may correspond to genuine lineage-specific or currently unannotated interactions. Complementary data sources, such as transcriptomic co-expression, could provide additional orthogonal support for these interactions. Similar results, though with lower precision, were observed at lower interaction score thresholds (S7 Table, S10 and S11 Figs).

Finally, the results show that intra-community precision slightly exceeded pairwise precision on average at all thresholds (e.g., 36.9% vs. 34.8% for *E. coli*, IntScore > 1.8), an indication that community-based analysis constitutes a reliable approach for predicting protein functional communities. When comparing species-specific vs. common PPIs, intra-community precision was lower for species-specific PPIs: 34.6% *vs.* 37.4% for *E. coli* and 27% *vs.* 35% for *S. enterica*. This reinforces the conclusion that, perhaps not surprisingly, species-specific PPIs identified by CM2.0 tend to correspond to less well-characterized interactions than those common across species.

Collectively, the results presented in this section show how pairwise interaction scores generated by CM2.0 can be leveraged to identify shared functional communities and potential species-specific relations. In the next section, we provide further details on some of the species-specific functional communities identified in *E. coli* and *S. enterica*.

### 2.3 Examples of species-specific functional communities

To further explore examples of functional protein communities with species-specific PPIs, we highlight two communities that are entirely species-specific. The first is the *phn* gene community, which drives the conversion of methylphosphonate into phosphate and methane [31] and was partially identified in *E. coli,* but not in *S. enterica* (Fig 5A). This is because the complex structure in *S. enterica* comprises different proteins [32] and the coevolutionary signals between them were not captured by our current set of parameters. The second community involves three electron transfer flavoproteins exclusive to *S. enterica* (STM0855, STM0856 and STM0858) [33] (Fig 5B). This *S. enterica*-specific community also includes yibF (STM3684), a glutathione S-transferase, and rfbI (STM2093), involved in O antigen modification as a putative ortholog of gtrB [34]. The identification of these communities highlights the capacity of PPI predictions to uncover complexes shaped by evolutionary pressures and ecological contexts specific to, in this case, *S. enterica*. RfbI’s role in O-antigen modification is functionally connected to lipopolysaccharide (LPS) biosynthesis, which plays a critical role in immune evasion and host-pathogen interactions [35]. Similarly, YibF is annotated as a glutathione S-transferase, a family of enzymes involved in detoxification and protection against oxidative damage [36]. The co-prediction of YibF and the electron transfer flavoproteins in this community suggests a possible link between redox homeostasis, energy metabolism, and species-specific stress adaptation in *S. enterica.*

**Fig 5:**
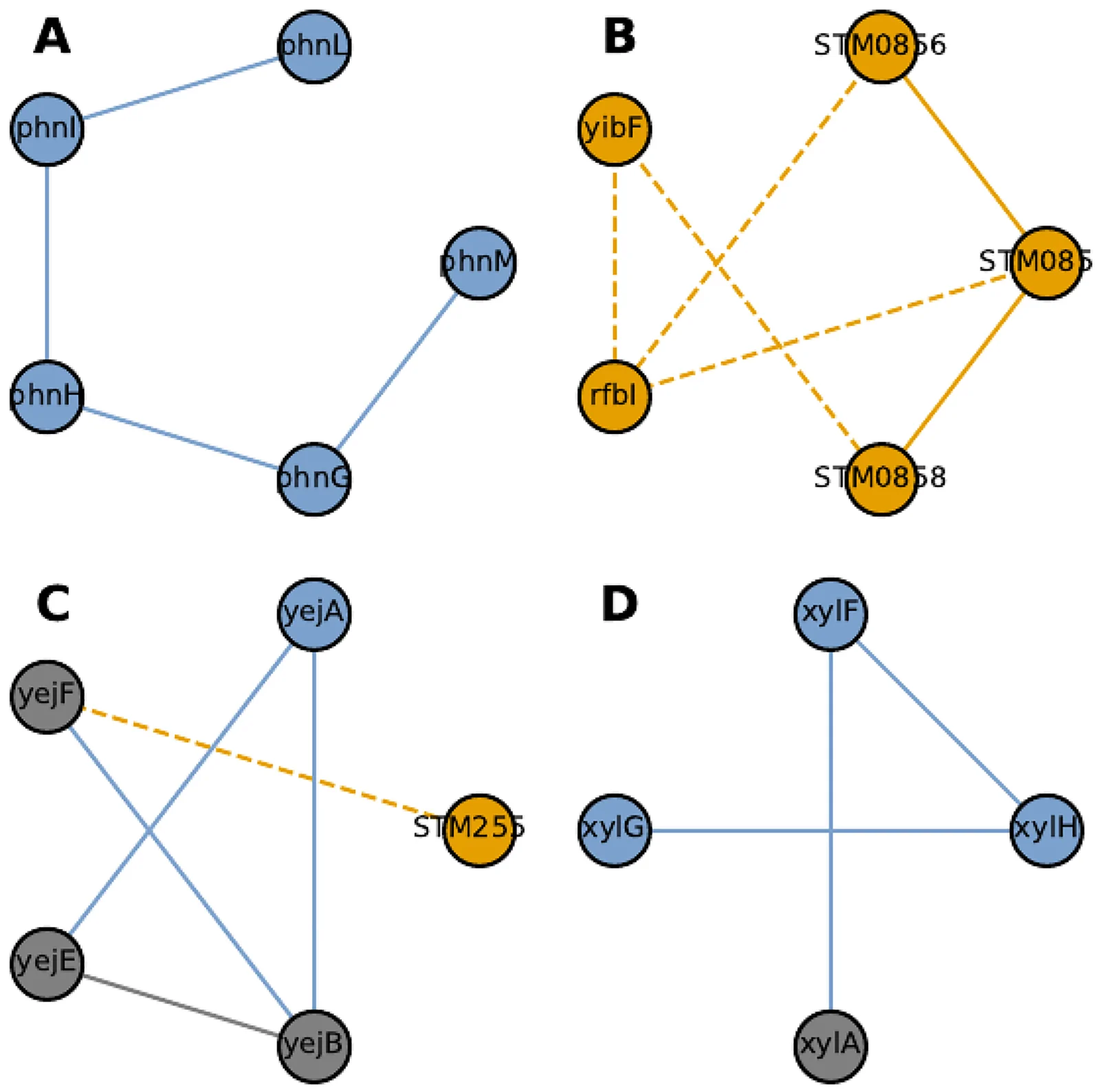
Examples of protein communities detected in the combined PPI network of *E. coli* and *S. enterica* at the threshold of IntScore > 1.7 containing species-specific PPIs. **A)** A community containing proteins from the *phn* gene cluster. **B)** A community containing three electron transfer flavoproteins (STM0855, STM0856 and STM0858). **C)** A community containing proteins encoded by the *yej* gene cluster. **D)** A community containing proteins encoded by genes from the *xyl* operon. The gray nodes/edges are common to both species, while the blue nodes/edges are only present in *E. coli* and the orange nodes/edges are only present in *S. enterica*. Solid edges indicate experimentally validated interactions and dashed edges indicate interactions without experimental validation.

Besides fully species-specific communities, there are species-specific PPIs within conserved functional communities. One example is a transporter complex composed of yejA, yejB, yejE and yejF (Fig 5C), which specifically recognizes the peptide component of Microcin C [37], an antibiotic produced by various microorganisms. These genes, present in both *E. coli* and *S. enterica,* confer resistance to antimicrobial peptides and contribute to virulence [38]. In the identified community, all proteins from the yejABEF complex are present. Notably, we observed a species-specific interaction involving an additional node exclusive to *S. enterica* (STM2551), which forms an uncharacterized interaction with YejF. This interaction suggests functional divergence between the transport systems in *S. enterica* and *E. coli*, as both the Yej complex and STM2551’s closest homolog (E-value = 5.8e-9) in *E. coli* (P76425 - rcnA), a nickel-cobalt efflux pump [39], are associated with the ATP-binding cassette (ABC) transporter family, involved in oligopeptide transport (e.g., microcin C) and nickel/cobalt homeostasis (KEGG PATHWAY: ko02010).

Finally, Fig 5D shows a community encompassing interactions between proteins encoded by the genes of the *xyl* operon, a key genetic component found in various bacteria that facilitates the metabolism of D-xylose [40]. While both *E. coli* and *S. enterica* have the xylA gene, *S. enterica* lacks the xylFGH transporter genes [41], suggesting alternative xylose transportation and metabolism mechanisms in this species. The presence of xylA in both species and its capability to operate independently highlight its significance in bacterial metabolism [40].

Highly conserved protein complexes, such as the flagellar assembly (*flg*), the ABC-transporter cassette (*ugp*), and the NADH:ubiquinone oxidoreductase (*nuo*) complexes (S12 Fig), were accurately identified across various interaction score thresholds. Their near-complete conservation of edges between *E. coli* and *S. enterica* reflects their essential roles in Gram-negative bacterial function. These examples show how CM2.0 can effectively filter predicted networks to identify biologically meaningful interactions. This approach offers valuable insights into species-specific adaptations and the intricate interplay between bacterial genomics and metabolism.

### 2.4 Multispecies PPI networks can be used to infer the function of unannotated proteins

To conclude our analyses, we selected well-characterized functional communities in *E. coli* and *S. enterica* (e.g., mur, pur, accb/fab) to showcase how CM2.0 predictions can enhance functional annotation of proteins in less-annotated species like *S. aureus.* We retrieved a list of interactors from EcoCyc [42] to reveal the predicted topology of these communities. This resulted in subnetworks of proteins encoded by well-annotated proteins in *E. coli* and *S. enterica,* which we compared with predictions in *S. aureus*. To refine annotations and discover additional functionally related proteins, we used a list of interactors as a proxy for identifying functional communities, moving beyond traditional operon-based approaches.

In Fig 6A, the conserved *mur* operon (murABCDEFI) [43] is shown. As expected, interactions between these proteins were identified across all species at relaxed thresholds and only partially at stricter thresholds (S13 Fig), indicating a very well-conserved coevolutionary signal. murG, the last enzyme in the peptidoglycan synthesis pathway [44], is crucial for bacterial cell wall biosynthesis. Unlike predictions for *E. coli* and *S. enterica*, murG is absent in the predicted interactome of *S. aureus*. However, the node WP_000160904.1 forms significant interactions with murA and murF, highlighting its central role in bacterial cell wall synthesis. In fact, there is a record of this sequence (WP_000160904.1) identified as a multispecies murG for all *Staphylococcus* species. Since murG is a promising antibiotic target [45], understanding differences in interaction patterns across these subnetworks could reveal potential vulnerabilities in the mur enzyme complex and provide valuable insights for developing antibacterial strategies.

**Fig 6:**
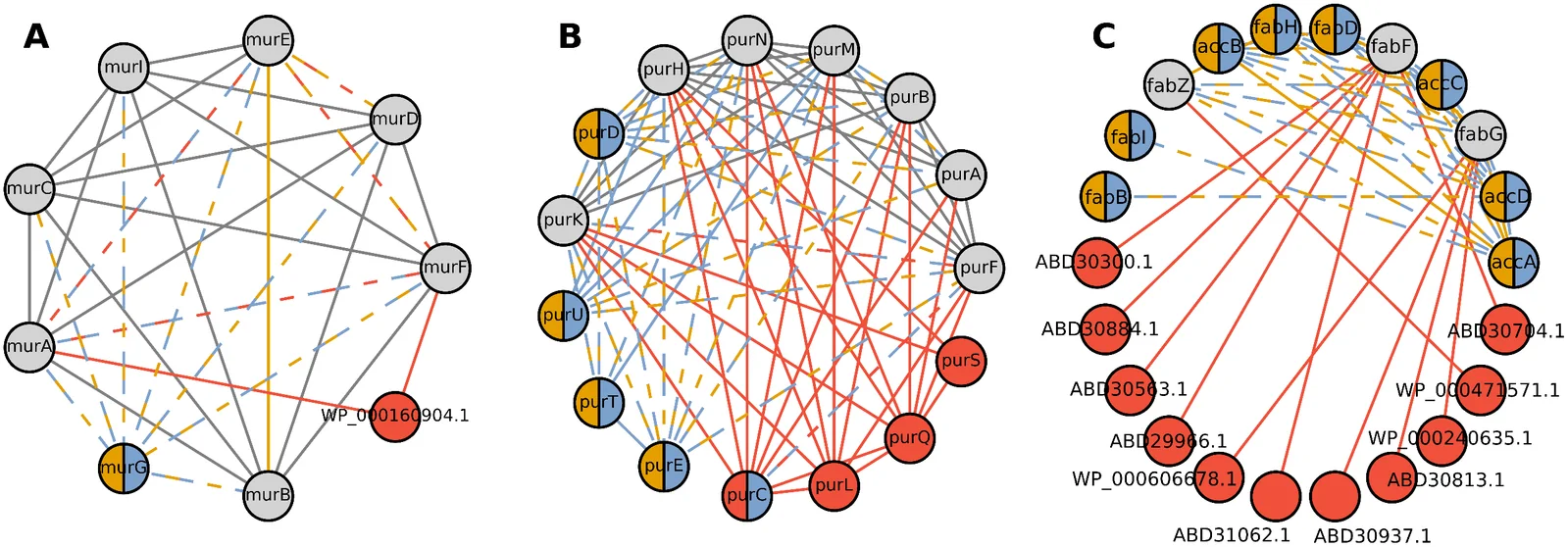
Predicted protein-protein interactions (PPIs) for the mur, pur, and acc + fab complexes in *E. coli*, *S. enterica*, and *S. aureus.* Interactions were filtered using the following interaction thresholds: **A)** 1.2 for the mur complex, **B)** 1.2 for the pur complex, and **C)** 1.7 for the acc + fab complex. Shared nodes and edges are shown in gray, *E. coli* predictions in blue, *S. enterica* predictions in orange, and *S. aureus* predictions in red. Solid edges indicate experimentally validated interactions and dashed edges indicate interactions without experimental validation.

Focusing on the *pur* family [46,47], involved in the purine biosynthesis, we identified a highly conserved cluster of proteins (purABCFHKMN) shared among the three species. This community was fully detected at a relaxed threshold of 1.2 (Fig 6B) but only partially at stricter thresholds (S14 Fig). Fig 6B shows that some nodes and edges (purDETU) are shared among Gram-negative bacteria, while purL, purQ, and purS are unique to the predicted interactome of *S. aureus*. These proteins constitute the phosphoribosylformylglycinamidine synthase complex (FGAR-AT complex / FGAM synthase complex). In Gram-positive bacteria and archaea, the FGAR-AT complex has three unique components (purS, purQ, purL), absent in Gram-negative bacteria [48]. The CM2.0 approach successfully identified these proteins only in the predicted network of *S. aureus*. Since the purine biosynthesis pathway is a promising antimicrobial target [49], this analysis could enhance the development of antibiotic treatments, particularly for Gram-positive bacteria with limited annotation.

As a final example, we examined interactions between proteins from the *fab* and the *acc* operons [50,51] (S15 Fig). Fig 6C shows that PPIs involving accABCD proteins were only retrieved for *E. coli* and *S. enterica*. However, these proteins are predicted to interact with nodes in the fab complex, also predicted in the poorly annotated proteome of *S. aureus*. This “bridge” node revealed a novel interaction in S. aureus: WP_000471571.1, potentially linked to the acc complex through its shared interaction with fabZ. Homology searches revealed that WP_000471571.1 matches the accD sequence (Q2YTE0) in *S. aureus* strain bovine RF122 / ET3–1, reported in the STRING database to interact with fabZ with a confidence score of 0.982 for *S. aureus* strain NCTC 8325 / PS 47.

We also identified several proteins of unknown function interacting with fabF. For example, ABD30300.1 likely encodes fatty acid kinase subunit A (fakA), which regulates virulence factor transcription and fatty acid incorporation into phospholipids [52,53]. Similarly, ABD30884.1 likely encodes pepP, a Xaa-Pro aminopeptidase and known virulence factor in *Campylobacter jejuni* [54]. ABD30563.1 may correspond to ypjA, an adhesin involved in adhesion and biofilm formation in *E. coli* [55], making it a promising candidate for *S. aureus* research. Another interesting interaction involves fabF and ABD29966.1, likely rnmV, a conserved 5S ribosomal maturase in low G + C Gram-positive bacteria [56,57]. Other notable interactions include ABD31062.1, a protein predicted to be membrane-associated in *E. coli* [58], and ABD30304.1, identified as a malonyl CoA-acyl carrier protein transacylase corresponding to fabD. These findings suggest diverse biological roles for fabF in *S. aureus* and merit further investigation.

Finally, fabG interacts with four unmapped proteins in *S. aureus*, namely WP_000606678.1, identified as Tripeptidase T (pepT) and reported for virulence importance [59]; ABD30937.1 (contains oxidoreductase HI0933 domain); WP_000240635.1, identified as a chaperonin whose inhibition is a promising antibiotic treatment [60]; and ABD30813.1, very likely spoT, a protein that increases survival under stress conditions in *E. coli* [61].

This section illustrates how multispecies PPIs inferred based on pairwise interactions predicted by CM2.0 can be used to infer the functional role of uncharacterized or unannotated proteins, which is particularly useful for poorly characterized organisms.

## 3 Discussion

Understanding the composition of protein complexes is crucial, yet there remains a significant knowledge gap in this area, particularly with respect to poorly annotated proteomes [1]. Current computational approaches, including homology-based annotation [62] and supervised ML algorithms, face considerable limitations when experimental data on protein interactions is scarce, hindering the prediction of new functions and the identification of species-specific interactions [10–12]. Moreover, ML approaches sometimes lack interpretability, and might suffer from the lack of negative examples for training [63]. CM2.0 offers a suitable alternative that does not require supervision and can produce functional annotations at the single-species level based on MSAs. CM2.0 uses an unsupervised coevolutionary filtering stage to exclude protein pairs lacking sufficient evolutionary information, i.e., insufficient number of sequences in the paired alignments, before ranking the retained pairs using a biologically informative interaction score. Our benchmarking analyses show that IntScore performs competitively with current state-of-the-art PPI prediction methods, while the preceding coevolutionary filtering stage substantially reduces the search space by preferentially retaining experimentally supported interactions.

One limitation of evolution based methods is their intrinsic inability to distinguish functional from physical interactions [64]. Coevolution may arise from direct physical interactions, but also from indirect functional relationships, participation in the same molecular complex, or membership in the same biological pathway. Consistent with this, we did not detect a significant correlation with the potential formation of physical complexes, as assessed by the quality of the corresponding structural models (S4 Fig). In other words, high IntScores did not necessarily correspond to confident structural models: some experimentally supported interactions with high IntScores yielded low-confidence AlphaFold Multimer models, whereas some low-IntScore pairs produced confident structural models (S16 Fig). This behavior reflects CM2.0’s design: the method is not intended for residue-level binding prediction, but for detecting large-scale coevolutionary and functional associations across the proteome. In any case, a full validation of the predicted complexes against structural predictions will remain a challenge dependent on the evolution of current methods [27,28], as pointed out in the recent CASP meeting [65,66]. To validate CM2.0, we used interaction annotations from different PPI databases alongside experimental data, treating functional and physical interactions equivalently. This validation set includes both direct physical interactions and indirect functional interactions (e.g., proteins in the same complex that do not contact directly) and is, therefore, adequate to CM2.0 that, as an evolution-based method, does not differentiate between physical and non-physical associations.

The benchmarking performed in this work, together with our analysis of the curated datasets of Cong *et al.* [6] and BioGRID, consistently show that CM2.0 enriches experimentally supported interactions above random expectation across diverse benchmarking scenarios. The comparison with RoseTTAFold2-PPI and D-Script further demonstrates that the interaction score itself performs competitively with current state-of-the-art methods, although the computational demands of these approaches limited the comparison to selected benchmark systems. In contrast, the principal limitation of the complete CM2.0 workflow lies in the amount of evolutionary information available for the initial coevolutionary filtering stage. This filter requires a minimum number of matching species, with a default threshold of 1000 for benchmarking (see Methods), although the parameter is adjustable and allows for broader coverage at the cost of statistical resolution. As sequence databases continue to expand, an increasing fraction of protein pairs is expected to satisfy the minimum evolutionary information required for reliable coevolutionary analysis. The benchmarking also illustrates this trade-off: the filter substantially reduces the search space and preferentially retains experimentally supported interactions, but becomes increasingly conservative when evolutionary information is limited.

Conceptually, CM2.0 acts not as a predictor of non-interactions but as a prioritization tool that ranks the most probable interacting pairs.

When analyzing bacterial proteomes, CM2.0 effectively prioritized true protein interactions, with around 40% of the top 1000 predictions matching known annotations from databases. These pairwise PPI predictions were leveraged to build multispecies PPI networks and automatically identify potential protein communities based on the pairwise interactions. Most of the high-confidence communities are present in multiple species and have a high density of interactions confirmed in databases. Some of these involve poorly annotated proteins, offering a first clue about their potential functions. Interestingly, we found a number of communities that are entirely species-specific. For example, the identification of the *phn* gene community in *E. coli* and the electron transfer flavoproteins in *S. enterica* illustrate how CM2.0 can uncover complexes that are relevant to specific metabolic and defensive mechanisms, pointing to host-pathogen interactions and unique metabolic challenges associated to different species. Unlike traditional approaches that rely on operon information to infer functional relationships, we focused on identifying functional communities through protein interactors, using them as a dynamic proxy for relatedness. This shift allows for a more flexible understanding of cellular organization, enabling the discovery of conserved functional relationships across species without the constraints of static gene clusters [67]. Our approach enhances the ability to uncover novel and context-specific interactions and provides a more dynamic and comprehensive view of cellular functions because it emphasizes functional communities rather than fixed operon structures.

Because CM2.0 relies on extensive phylogenetic-tree computations, efficiency was a key design consideration. The use of the FastTree algorithm allows tree inference to be performed orders of magnitude faster than traditional likelihood-based methods, making large-scale analyses feasible. A detailed breakdown of runtime is provided in S8 Table.

Another limitation of the current framework is that CM2.0 was developed and validated using bacterial proteomes. Although the overall framework can, in principle, generate a putative list of protein interactions for any proteome as long as reliable orthologous relationships can be established, eukaryotic genomes present additional challenges, particularly the widespread occurrence of paralogous genes, which can dilute coevolutionary signals. Moreover, CM2.0 assumes that orthologous proteins follow tree-like evolutionary histories, so processes such as horizontal gene transfer may introduce confounding coevolutionary signals. However, it is also possible that these events reflect biologically meaningful co-transferred functional modules, as previously observed in phylogeny-based coevolution analysis [68], distinguishing between these scenarios is outside the scope of the current framework.

To summarize, CM2.0 is an unsupervised coevolutionary prioritization framework that leverages evolutionary information to prioritize biologically meaningful protein interactions at the proteome scale. Beyond pairwise predictions, the resulting interaction network can be used to uncover conserved clusters that may include previously poorly annotated proteins, while at the same time, it identifies potentially biologically relevant species-specific interactions and protein communities. Thus, CM2.0 can contribute to the annotation of orphan proteins and the identification of new complexes in understudied organisms. Our predictions rely on an intuitive confidence score, i.e., interaction score, which correlates with the reliability of the interactions and can serve to guide future experimental and computational research. CM2.0 opens the door to future applications extending the range of predictions to less well-known species, beyond the species explored here. Future developments may also benefit from deep learning strategies such as graph neural networks for sequence representation learning, which could provide complementary perspectives to coevolution-based approaches.

## 4 Methods

### 4.1 ContextMirror2.0

The ContextMirror2.0 method (Fig 7) consists of an initial prefiltering stage followed by three computational steps. First, an initial coevolutionary network is generated by calculating the tree similarities for every protein pair in the input file. Next, an optimized coevolution measure is derived for each protein pair by analyzing their coevolutionary patterns relative to all other proteins. Finally, the influence of additional proteins on the coevolution of a given pair is assessed. Each step is described in detail below.

**Fig 7:**
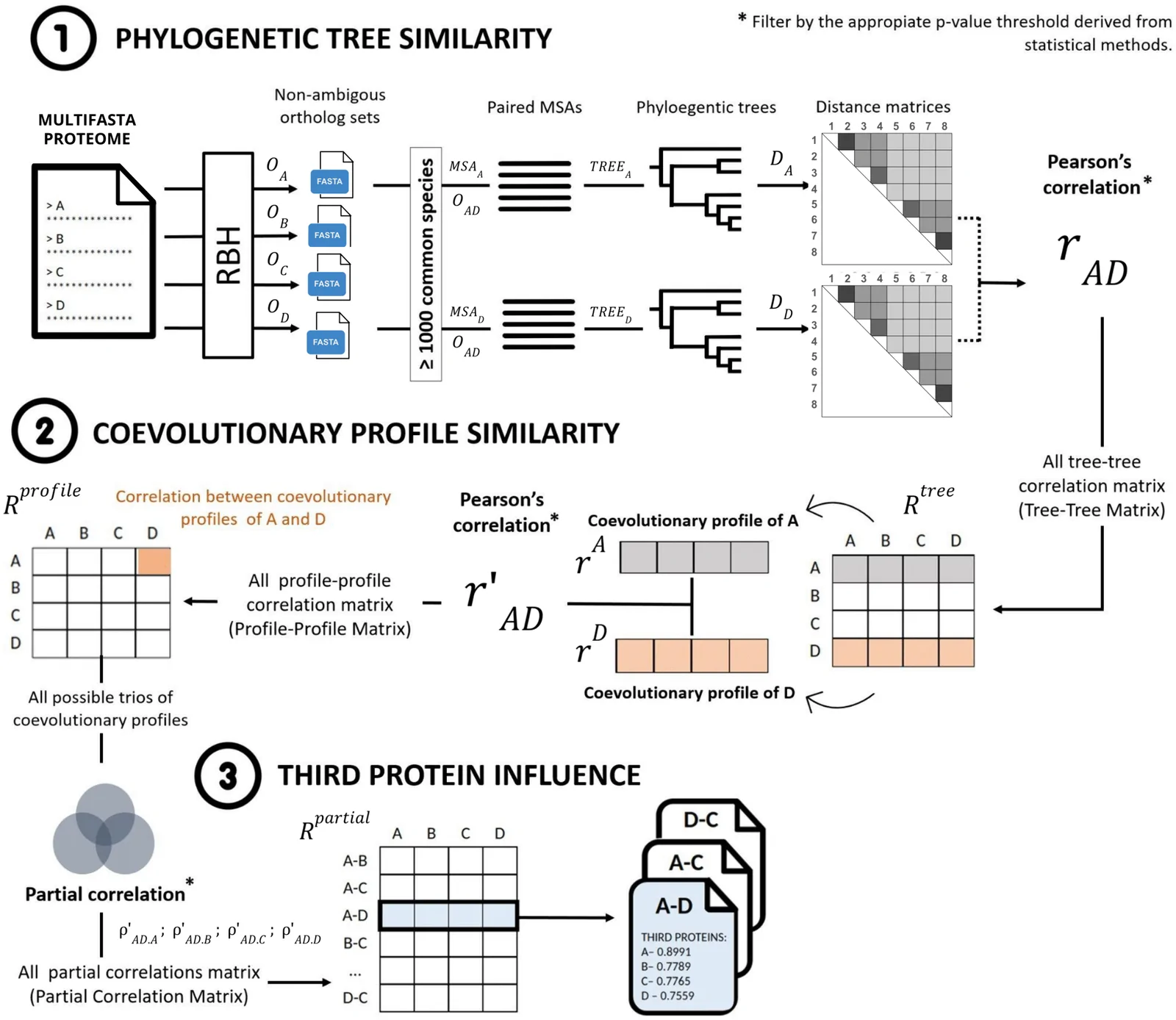
ContextMirror2.0 pipeline. The figure illustrates the overall workflow of the ContextMirror2.0 pipeline, with key steps outlined. Detailed descriptions of steps 1, 2, and 3 are provided in the Methods section (section 4.1, steps 1, 2, and 3, respectively).

*Step 0: Ortholog retrieval, paired MSA construction and prefiltering*

For each protein *p*, an ortholog set $O_{p}$, is retrieved using the standard Blast Reciprocal Best Hit (RBH) approach [69] with a cutoff e-value of 10^-5^, requiring an alignment of at least 70% of the query protein length, with a minimum 60% query coverage and at least a 30% of sequence identity. The forward Blast step is performed on the full NCBI’s non-redundant database (25/03/2022), while the reverse Blast is performed against the original input set of sequences.

For each possible protein pair *(p, q)*, the set of species with shared orthologs is defined as $O_{pq}$:


$$\begin{array}{c} O_{pq}=O_{p}\cap O_{q} \end{array}$$

Protein pairs are then filtered to contain a minimum number of shared species in their ortholog sets so that only pairs where $O_{pq}\geq N_{min}$, where $N_{min}$ is a user-defined threshold (default: 1000; see S9 Table) are kept. In these cases, paired multiple sequence alignments (MSAs) are constructed using only the sequences corresponding to the overlapping species in $O_{pq}$. This filtering step removes protein pairs lacking sufficient evolutionary information for subsequent coevolutionary analyses.

*Step1: All vs. all tree similarities (Tree-tree correlation -* $R^{tree}$)

Following the initial filtering stage, a network of pairwise tree similarities is computed for all retained protein pairs using the MirrorTree approach [68]. For each retained protein pair, a distance matrix (D) is obtained from the paired MSA using the McLachlan amino acid residue homology matrix [70], and the similarity between the two protein families is calculated as the Pearson’s linear correlation between the two matrices [71] as follows:


$$\begin{array}{c} r_{pq}=\frac{\sum_{i=\text{1}}^{n}\left(D_{i}^{p}-\underset{―}{D^{p}}\right)\cdot \left(D_{i}^{q}-\underset{―}{D^{q}}\right)}{\sqrt{\sum_{i=\text{1}}^{n}{\left(D_{i}^{p}-\underset{―}{D^{p}}\right)}^{\text{2}}\cdot }\sqrt{\sum_{i=\text{1}}^{n}{\left(D_{i}^{q}-\underset{―}{D^{q}}\right)}^{\text{2}}}} \end{array}$$

where $n=\frac{\left(N^{\text{2}}-N\right)}{\text{2}}$ and ${N=O}_{pq}$ is the number of shared orthologs (i.e., sequences in the paired MSA). $D_{i}^{p}$ and $D_{i}^{q}$ are the pairwise evolutionary distances between orthologs in the distance matrices derived from the MSAs of proteins *p* and *q*, respectively. $\underset{―}{D^{p}}$ and $\underset{―}{D^{q}}$ denote the mean distances across all *n* values for each protein.

Each correlation value $r_{pq}$ is considered significant based on a p-value cut-off of 10^-5^, as established in the original ContextMirror implementation [22]. As a result, a square matrix containing all tree-tree significant correlation values (raw tree similarities) is obtained.

*Step2: All vs all coevolutionary profile similarities (Profile-profile correlation -* $R^{profile}$)

If protein pairs are treated as independent interactors, a high rate of false positives is expected [72]. To avoid that, the full matrix of coevolutionary information is used to evaluate the coherence of the coevolutionary signal between a pair of protein families. In other words, protein pairs will only be considered as interacting if the comparison of the coevolutionary contexts are similar, i.e., if their pattern of coevolution with other proteins is similar. To evaluate that, we calculate the Pearson correlation between the coevolutionary profiles of the trees. A coevolutionary profile is a vector $r^{p}$ that contains the tree similarity values between a given protein *p* and all other proteins *i* in the input set, i.e., a specific row/column of the matrix that was generated in the first step (MirrorTree). The updated correlation coefficient ${r^{\prime }}_{pq}$ is then calculated as follows:


$$\begin{array}{c} {r^{\prime }}_{pq}=\frac{\sum_{i=\text{1}}^{M}\left(r_{i}^{p}-\underset{―}{r^{p}}\right)\cdot \left(r_{i}^{q}-\underset{―}{r^{q}}\right)}{\sqrt{\sum_{i=\text{1}}^{M}{\left(r_{i}^{p}-\underset{―}{r^{p}}\right)}^{\text{2}}}\cdot \sqrt{\sum_{i=\text{1}}^{M}{\left(r_{i}^{q}-\underset{―}{r^{q}}\right)}^{\text{2}}}} \end{array}$$

where M is the number of proteins that are significantly correlated (in MirrorTree) with both proteins *p* and *q*, $r_{i}^{p}$ and $r_{i}^{q}$ are correlation values between proteins *p* and *i*, and *q* and *i*, respectively; and $\underset{―}{r^{p}}$ and $\underset{―}{r^{q}}$ their respective means. Since this is done for all possible pairs of proteins, the result of this step is a NxN matrix where the correlation between all pairs of significantly correlated coevolutionary profiles is included.

In the original ContextMirror implementation [22], homologs were searched within a set of 116 reference genomes, and protein pairs with at least 15 species in common were considered significant. In that setting, profile–profile correlations (r′) were evaluated using a threshold of p ≤ 10^−5^. In our case, however, the use of the full non-redundant database and the requirement of ≥1,000 common species per pair produces much longer coevolutionary profiles. Statistically, the increased dimensionality translates into many more degrees of freedom, which drives the p-values to be extremely small even for weak correlations. We verified empirically that with a raw cut-off of p ≤ 10^−5^, essentially all correlations passed and the filter became uninformative. To correct for this effect, a Bonferroni correction is applied, which is a statistical adjustment that reduces the chance of obtaining false-positive results by lowering the threshold for statistical significance in proportion to the number of comparisons [73]. After the Bonferroni correction the p-value threshold in this matrix is set to 10^−11^, making the criteria for significance more stringent and thus reducing the likelihood of false positives.

*Step3: Influence of all profiles in every profile-profile correlation (Partial correlation -* $R^{\partial }$)

In this step, it is analysed if the coevolutionary signal between two proteins could be attributed to broad evolutionary tendencies involving many proteins. If correlation is not attributable to any other third protein, it means the signal is highly specific, and thus a probable indicator of a close functional and structural relationship. However, the components of protein complexes influence one another and therefore, patterns of third-protein influence will reveal relationships within these complexes, and with others as well.

To separate specific from general coevolution, the partial correlation coefficient between every pair of proteins and the rest of the proteome is calculated. The partial correlation coefficient between two proteins (*p and q*) given a third protein (k) is calculated as follows:


$$\begin{array}{c} {\rho^{\prime }}_{pq.k}=\frac{{r^{\prime }}_{pq}-{r^{\prime }}_{pk}{\cdot r^{\prime }}_{qk}}{\sqrt{\left(\text{1}-{{r^{\prime }}_{pk}}^{\text{2}}\right)\cdot \left(\text{1}-{{r^{\prime }}_{qk}}^{\text{2}}\right)}} \end{array}$$

where ${\rho^{\prime }}_{pq.k}$ is the partial correlation between the coevolutionary profiles of proteins *p* and *q* with a constant coevolutionary profile of protein *k*; and ${r^{\prime }}_{pq}$, ${r^{\prime }}_{pk}$ and ${r^{\prime }}_{qk}$ are the profile–profile correlation values between *p* and *q, p* and *k,* and *q* and *k*, respectively. In this step, only partial correlations with a p-value smaller or equal to 10^−6^ were considered significant, following the original implementation [22].

As a result, for each protein pair, a list of partial correlations with third proteins whose partial correlations remain statistically significant (p ≤ 10^−6^) is produced. This list displays the number of third proteins for which the partial correlation between the coevolutionary profiles of a pair of proteins remains significant, and the magnitude of the remaining association, represented by the partial correlation coefficient. This way, protein pairs retaining significant partial correlations for only a few third proteins represent more specific coevolutionary relationships, whereas pairs retaining significant partial correlations for many third proteins are more likely to belong to broader functional modules.

The final product of this implementation is a ranked list of predicted protein-protein interactions (PPIs). These PPIs are ranked by the level of confidence that they are true interactions, rather than artifacts produced by the implementation process. This ranking system is based on the integration of coevolutionary information obtained from each of the steps. During the initial prefiltering stage, we reduce all possible combinations of proteins to those that share orthologs in at least 1000 species and show a significant correlation between their evolutionary histories. The second step produces a correlation value for each of the considered pairs of coevolutionary profiles. This correlation value is added to the average of partial correlations of third proteins for each of the predictions to create an interaction score. The higher the interaction score, the more coevolutionary related these two proteins are and thus, the more likely it is that they are related functionally or structurally.

Finally, we observed that the predictions influenced by very few third proteins tended to be false positives (or true positives not recorded yet). To further increase the ranking power of this interaction score, a final update was introduced. A Mann-Whitney-U-Test was performed on the data and we found out that predictions influenced by a small number of third proteins are significantly more likely to be false positive (Statistic = 23651311893.500, p < 0.001). The rationale behind this penalization strategy is based on the understanding that highly specific protein-protein interactions (PPIs) should still reflect influence from other proteins within the same system. While the impact of third proteins on such PPIs may be weak, it should still be significant if the interaction occurs naturally. Conversely, PPIs that appear strongly correlated but show no significant partial correlations after controlling for third proteins are unlikely to represent true biological interactions and are more likely artifacts of the method or other biases. This penalization approach adjusts for such cases by reducing the interaction score of protein pairs with no substantial third-protein influence (≤ 50 third proteins), effectively lowering the rank of potentially spurious predictions. The final list is then rearranged according to the Interaction Score (IntScore) considering this penalty, producing the final output of the ContextMirror updated implementation as follows:


$$\begin{array}{c} {IntScore}_{pq}={r^{\prime }}_{pq}+n^{-\text{1}}\sum_{i=\text{1}}^{n}{\rho^{\prime }}_{pq.i}-\delta (n) \end{array}$$

With the penalty termδ(n) defined as:


$$\begin{array}{c} \delta (n)=0.1-(0.1-0.002)*(n-1)/49\;for\;n\leq 50,\;and\;\delta (n)=0\;for\;n>50. \end{array}$$

Where *p* and *q* represent the proteins being evaluated, and *n* is the number of third profiles for which the partial correlation remains statistically significant. ${r^{\prime }}_{pq}$ is the correlation value extracted from the profile–profile correlation matrix, and ${\rho^{\prime }}_{pq.i}$ is the partial correlation between the coevolutionary profiles of proteins *p* and *q*, controlling for a third protein *i*.

The penalty term δ(n) is applied only when n≤50, decreasing linearly from 0.1 (for interactions influenced by only one third protein) to 0.002 (for those influenced by 50). For interactions supported by more than 50 third proteins, the penalty is set to zero. This strategy penalizes weakly supported predictions while preventing score inflation for well-supported ones.

### 4.2 Analysis of the predicted PPIs

*Benchmarking*

As a benchmarking, we predicted the interactomes of three bacterial species from their reference proteomes: *Escherichia coli* (strain *K12 MG1655*), *Salmonella enterica* (subsp. enterica serovar *Typhimurium LT2*) and *Staphylococcus aureus* (strain *NCTC 8325* / *PS 47*), compared to the ground truth provided by experimental results stored in a combination of databases: IntAct [74], STRING [75], EcoCyc [42] and Interactome3D [76]. Interactions with score below 0.2 were filtered out from the STRING database to improve the quality of the data. For *S. aureus*, due to insufficient strain-specific data, experimental information from experimental findings at the species level were also incorporated [77].

The precision is calculated as the number of true positive predictions divided by the number of total predictions (true positives and false positives). The recall is calculated as the number of true positive predictions divided by the number of relevant cases. For intra-community calculations, only edges contained in the retrieved communities are considered. For the combined network precision analysis in Fig 4 and S7 Table, communities were automatically retrieved 100 times to calculate the mean and standard deviation. The selected communities in Figs 5, 6, S9–S15 were retrieved with the random seed that optimized the precision for shared predictions (seed = 76) as follows:


$$\begin{array}{c} \frac{\left(TPTP+TPFP+FPTP\right)}{\left(TPTP+TPFP+FPTP+FPFP\right)} \end{array}$$

where the numerator represents any interaction that is a true positive in any of the datasets, and the denominator represents all predictions made in both datasets.

The chance precision for a given network is calculated as the number of true positive predictions divided by the number of potential pairs formed by all the nodes in the network (S10 Table).

*Dependency of the interaction score on MSA diversity*

To assess the dependency of the interaction scores on the number of sequences contained in the paired MSAs, we quantified the sequence diversity of the MSAs defined by number of effective sequences (N_eff_) as follows:


$$\begin{array}{c} N_{eff}=\frac{\text{1}}{\sqrt{L}}\sum_{n=\text{1}}^{n}\frac{\text{1}}{\text{1}+\Sigma_{m=\text{1},m\neq n}^{N}I\left[s_{m,n}\geq \text{0}.\text{8}\right]} \end{array}$$

where *L* is the length of the query sequence, *N* is the number of sequences contained in the MSA, *S*_*m,n*_ is the sequence identity between the *m*^th^ and *n*^th^ sequences, and *I*[.] represents the Iverson bracket, which takes the value *I*[*S*_*m,n*_  ≥  0.8]  =  1 if *S*_*m,n*_  ≥  0.8, and 0 otherwise. After calculating N_eff_ for MSA A and MSA B in a given paired MSA, we defined the N_eff_ of the paired MSA as the minimum between N_eff_ A and N_eff_ B.

*Dependency of the interaction score on the number of matching-species between homolog sets*

Throughout the manuscript, *matching species* refers to the number of species belonging to the intersection of the ortholog sets of the two proteins ($O_{pq}$) and therefore corresponds to the number of sequences available to construct the paired MSA. Protein pairs were grouped according to $O_{pq}$, and within each bin, IntScore distributions were compared between validated and unsupported interactions (S3 Fig - left). To evaluate whether IntScore provided predictive information beyond the number of matching-species, logistic regression models were fitted using interaction status as the response variable, while IntScore and $O_{pq}$ were used as explanatory variables (S2 Table).

*Dependency of the interaction score on intergenic distance*

Genomic coordinates for *E. coli, S. enterica* and *S. aureus* were obtained from BioCyc [78]. For each protein pair, the intergenic distance was calculated as the shortest genomic distance separating the two coding sequences (distance between the end coordinate of the upstream coding sequence and the start coordinate of the downstream coding sequence. Zero for overlapping genes). Protein pairs were grouped according to intergenic distance, and within each bin, IntScore distributions were compared between validated and unsupported interactions (S3 Fig - middle). To evaluate whether IntScore provided predictive information beyond genomic proximity, logistic regression models were fitted using interaction status as the response variable, while IntScore and intergenic distance were used as explanatory variables (S2 Table).

*Dependency of the interaction score on operon membership*

Operon annotations for *E. coli* were obtained from ODB4 [79], whereas transcriptional unit annotations for *S. enterica* and *S. aureus* were retrieved from BioCyc [78]. Protein pairs were classified according to whether both proteins belonged to the same annotated operon (or transcriptional unit). The distribution of IntScores among intra and inter-operon pairs was evaluated (S3 Fig - right) and quantified as the numbers of validated same-operon and non-same-operon interactions recovered above IntScore thresholds of 1.6, 1.7 and 1.8 (S3 Table).

*Structural analysis*

For a number of predicted PPIs, structural models were generated using AlphaFold2.3.0 [28]. For our analysis, we used the top-ranked unrelaxed model provided by AlphaFold. We adhered to the default settings for multiple sequence alignment (MSA) generation and the prescribed number of recycles. Our final dataset included 60 protein dimer complexes, with 20 each derived from *Escherichia coli*, *Staphylococcus aureus*, and *Salmonella enterica*. The selection criteria for the 20 proteins from each species was based on the interaction score. For each species, the top10 and bottom10 PPIs were selected. For each protein complex, we calculated the pDockQ2 score as detailed in [29], the improved version of pDockQ metric, designed to evaluate the quality of modelled protein complex models.

To evaluate the accuracy of the complex models, we identified the minimum pDockQ2 score for each protein pair (S4 Fig - top). We adopted the threshold of 0.23, as recommended by the creators of pDockQ2, to signify a high-accuracy complex. Those complexes exceeding this threshold were chosen for detailed structural representation in S4 Fig - bottom.

Full details regarding the protein complex IDs, their interaction scores, and the corresponding pDockQ2 scores are provided in S11 Table.

*Benchmarking against RoseTTAFold2-PPI and D-Script*

To compare CM2.0 with current state-of-the-art PPI prediction methods, we benchmarked it against RoseTTAFold2-PPI and D-Script using four experimentally characterized bacterial protein complexes: the Nuo and ATP synthase complexes from *E. coli* and the Dpp and Liv transporters from *S. enterica*. For each complex, all possible protein pairs involving complex members were generated together with negative-control pairs formed between complex proteins and unrelated cytosolic proteins (S13 Table). All pairs were evaluated using RoseTTAFold2-PPI and D-Script, while CM2.0 predictions were obtained from the corresponding whole-proteome interactomes.

The primary benchmark evaluated the ability of each method to discriminate experimentally validated interactions from unsupported interactions. Because RoseTTAFold2-PPI and D-Script assign scores to every protein pair, but CM2.0 first applies a coevolutionary filtering stage, two complementary comparisons were performed. In the first, only protein pairs assigned an IntScore by CM2.0 were considered, which allows the comparison of the discriminative power of the scoring functions on the same candidate interaction space (S4 Table). In the second, protein pairs discarded by the CM2.0 coevolutionary filters were assigned an IntScore of 0 to enable comparison of the complete prediction workflows (S5 Table). This assignment was used exclusively for benchmarking purposes and should not be interpreted as the biological meaning of a missing IntScore.

Because interaction databases may be incomplete, the benchmark was repeated using complex membership as an independent proxy for biological interaction, classifying intra-complex pairs as positives and complex-control pairs as negatives (S6 Fig). The same benchmarking strategies were applied (S4 and S5 Tables). To characterize the behaviour of the CM2.0 filtering stage, contingency tables comparing retained and filtered interactions with validated and unsupported interactions were constructed, from which true-positive recovery, unsupported-interaction filtering rates, Fisher’s exact tests and odds ratios were calculated (S6 Table).

*Multispecies PPI networks*

Based on the pairwise interaction scores outputted by CM2.0 we build a network in which each node represents an individual protein, and the edges denote the predicted interactions between them and are weighted by the interaction score. We conducted community searches using the Louvain algorithm recursively (resolution = 1) to identify protein communities (maximum community size = 30 nodes) either in individual or in combined networks. We also used the network to identify the sets of interactions made between certain proteins of interest in different species.

To enable comparisons between proteins from distinct proteomes, we identified the closest homologs in the other species. Using these homolog lists, proteins across species could be compared as considered equivalent when they are found to be a reciprocal best hit within the top 5 closest homologs for each pairwise comparison. For each of the proteins, a custom proteome database was created using the “makeblastdb” tool from the BLAST+ suite [80] and standard blastp parameters were used to retrieve homologs from each corresponding combination of species. S12 Table gathers the results of these homolog lists.

For the combined networks of *E. coli* and *S. enterica*, nodes were superimposed according to the reciprocal best hit relationships calculated earlier. The color of the nodes indicate the network of origin, blue indicating *E. coli,* orange denoting *S. enterica* and red for *S. aureus*. Gray nodes represent reciprocal best hits. Edges follow the same logic and additionally, the edge can either be represented as a solid line (validated interaction) or a dashed line (unvalidated interaction). When predicted in both, the weight of the nodes is the average of the interaction scores predicted in both individual networks.

### 4.3 Technical specifications

*Software requirements*

We used BLAST version 2.13 to search the NCBI database for potential orthologs and MAFFT (Multiple Alignment using Fast Fourier Transform) version 7.471 to build MSAs [81]. The specifications of the alignment design include the *automatic* parameter that automatically chooses the best strategy between progressive (FFT-NS-2) and iterative (FFT-NS-I or L-INS-i) methods, and the *anysymbol* option that considers ambiguous residues (e.g., B, Z, J, X, period, …). The Python version employed to run this program is 3.6.1 with the following dependencies: os, sys, Bio v1.79 (SeqIo, AlignIo, Phylo, ClustalWCommandline and DistranceTreeConstructor), numpy v1.21.5, networkx v2.8.5, requests v2.28.1, pandas v1.4.3, community v0.16 and scipy v1.8.1. Additionally, FastTree v2.1 and csvkit v1.3.0 are needed. Any parallelization tool (e.g., GREASY) is recommended to increase speed.

Many of these are novel additions to the pipeline, as numerous beneficial tools have been developed since the original method was published. These adaptations include the update of the alignment software from MUSCLE to MAFFT, an overall faster and more consistent method [82]. Likewise, we replaced ClustalW with FastTree [83] as the phylogenetic-tree-building algorithm. Also, since the size of databases has increased drastically over the past years, the GREASY parallelization strategy was implemented to reduce the computing time and manage large datasets. Moreover, the update of the BLAST software and the local installation of databases significantly increased the speed of RBH operations.

Details regarding the resource usage and computing time allocated for predicting the three interactomes are presented in S8 Table.

## Supporting information

S1 Fig Correlation between the Profile-Profile Correlation and the number of third proteins for every predicted pair in *Escherichia coli* (A), *Salmonella enterica* (B) and *Staphylococcus aureus* (C). The green line represents the exponential curve fitted to the distribution of points in the scatter plot. The correlation displayed in the plots reflects the Pearson correlation between the Profile-Profile Correlation and the number of third proteins for which the partial correlation remains statistically significant for each pair.
(TIF)

S2 Fig Correlation between the Interaction Score and the minimum number of effective sequences (Min Nf) for 10000 random predicted pairs in *Escherichia coli* (A), *Salmonella enterica* (B) and *Staphylococcus aureus* (C). The green line represents the linear regression line fitted to the distribution of points in the scatter plot. The coefficient of determination (R²) for each model is displayed for each case.
(TIF)

S3 Fig IntScore captures predictive information beyond matching-species count, genomic proximity and operon organization. Protein pairs were stratified according to the number of matching species in the paired multiple sequence alignment (left) and intergenic distance (middle). Within each bin, experimentally validated interactions consistently received higher IntScores than unsupported interactions, resulting in higher precision than random selection from the same bin. Asterisks above each pair of violin plots indicate the significance of one-sided Mann–Whitney U tests (*P* < 0.05, P < 0.01, *P* < 0.001, P < 0.0001); ns indicates a non-significant comparison, typically due to the small number of protein pairs in that bin. (Right) operon composition of the highest-ranked CM2.0 predictions, displaying that the majority of high-confidence predictions correspond to inter-operon protein pairs (Incomplete refers to protein pairs in which one of the interactors belongs to an unknown operon. Unknown refers to cases in which there is no operon information for either of the interactors).
(TIF)

S4 Fig (top) Results of minimum pDockQ2 score per PPI versus interaction score. Dashed line is the threshold of the pDockQ2 score (0.23) to be considered as a prediction of likely interaction. Predictions of AF Multimer for 20 proteins of *E. coli*, *S. aureus* and *S. enterica* each, 10 with high and 10 with low ContextMirror Interaction Score. S11 Table contains the data used to produce this plot. **(bottom)** AlphaFold Multimer models with highest min pDockQ2. A) OppB (P0AFH2) - OppC (P0AFH6) Oligopeptide transport system in *E. coli*. B) OppB (P08005) - OppC (P08006), Oligopeptide transport system permease protein OppB-OppC in S. enterica. C) UgpA (P10905) - UgpE (P10906) glycerol uptake system in *E. coli*. None of the produced models are in PDB.
(TIF)

S5 Fig Score distributions for validated and unsupported protein pairs in the Atp (A), Dpp (B) and Liv (C) benchmarks. Violin plots compare RoseTTAFold2-PPI (left), D-Script (middle) and CM2.0 (right) IntScore for the subset of protein pairs evaluated by CM2.0 after coevolutionary filtering.
(TIF)

S6 Fig Score distributions for same-complex (e.g., nuo-nuo) and out-of-complex protein pairs (e.g., nuo-Control) in the Nuo (A), Atp (B), Dpp (C) and Liv (D) benchmarks. Violin plots compare RoseTTAFold2-PPI, D-Script and CM2.0 IntScore for the subset of protein pairs evaluated by CM2.0 after coevolutionary filtering.
(TIF)

S7 Fig Behaviour of the CM2.0 coevolutionary filtering stage across the benchmark protein complexes. Histograms show the number of database supported and unsupported protein pairs retained by or removed during the initial coevolutionary filtering stage for the Nuo and ATP synthase complexes from *E. coli* and the Dpp and Liv transporters from *S. enterica*. Across all benchmark systems, experimentally supported interactions were preferentially retained over unsupported interactions. Corresponding retention counts and Fisher’s exact test odds ratios are reported in S6 Table.
(TIF)

S8 Fig Proportion of shared PPIs at different interaction score thresholds for the three pairwise comparisons. (TIF)

S9 Fig Communities retrieved using the Louvain algorithm on the union of the predicted interactomes for *E. coli* and *S. enterica* with an interaction score threshold of 1.8. Common nodes and edges are colored gray, blue indicates predictions exclusive of *E. coli* and orange shows unique predictions for *S. enterica*.
(TIF)

S10 Fig Communities retrieved using the Louvain algorithm on the union of the predicted interactomes for *E. coli* and *S. enterica* with an interaction score threshold of 1.7. Common nodes and edges are colored gray, blue indicates predictions exclusive of *E. coli* and orange shows unique predictions for *S. enterica*.
(TIF)

S11 Fig Communities retrieved using the Louvain algorithm on the union of the predicted interactomes for *E. coli* and *S. enterica* with an interaction score threshold of 1.6. Common nodes and edges are colored gray, blue indicates predictions exclusive of *E. coli* and orange shows unique predictions for *S. enterica*.
(TIF)

S12 Fig Functional communities retrieved from the combined network of *E. coli* and *S. enterica* predicted interactomes at different interaction score thresholds. A) Flagellar assembly complex (IntScore > 1.7). B) ABC transporter complex UgpBAEC (IntScore > 1.6). C) NADH:ubiquinone oxidoreductase complex (IntScore > 1.6).
(TIF)

S13 Fig Predicted protein-protein interactions (PPIs) for the Mur complex in *E. coli, S. enterica,* and *S. aureus*, filtered with interaction thresholds of 1.2 (A), 1.5 (B), and 1.7 (C). Shared nodes and edges are depicted in gray, *E. coli* predictions are shown in blue, *S. enterica* predictions are highlighted in orange, and *S. aureus* predicted interactions are indicated in red.
(TIF)

S14 Fig Predicted protein-protein interactions (PPIs) for the Pur complex in *E. coli, S. enterica*, and *S. aureus*, filtered with interaction thresholds of 1.2 (A), 1.5 (B), and 1.7 (C). Shared nodes and edges are depicted in gray, *E. coli* predictions are shown in blue, *S. enterica* predictions are highlighted in orange, and *S. aureus* predicted interactions are indicated in red.
(TIF)

S15 Fig Predicted protein-protein interactions (PPIs) for the acc + fab complex in *E. coli, S. enterica*, and *S. aureus*, filtered with interaction thresholds of 1.5 (A), 1.6 (B). (**C**) Predicted protein-protein interactions (PPIs) for the acc + fab complex in *E. coli, S. enterica*, and *S. aureus*, filtered with interaction thresholds of 1.7, including unmapped proteins in *S. aureus*. Shared nodes and edges are depicted in gray, *E. coli* predictions are shown in blue, *S. enterica* predictions are highlighted in orange, and *S. aureus* predicted interactions are indicated in red.
(TIF)

S16 Fig AlphaFold Multimer model of P33355 - P76502 (yehS - sixA) validated structurally (min pDockQ2 = 0.562067), but predicted to have a low interaction score (IntScore = 0.40), yet not reported as an interaction in *E. coli* (TN). (TIF)

S1 Table Pairwise precision at different interaction score (IntScore) thresholds for different interactome predictions at various interaction score thresholds. (XLSX)

S2 Table Logistic regression models evaluating IntScore as predictor of interaction status after controlling for the number of matching species in the paired MSA (left) or intergenic distance (right). (XLSX)

S3 Table Number of predicted intra-operon and inter-operon protein pairs at different IntScore thresholds (≥1.6, ≥ 1.7 and ≥1.8) for *E. coli*, *S. enterica* and *S. aureus.* Precision values are reported separately for the intra-operon and inter-operon subsets.
(XLSX)

S4 Table AUC values for CM2.0, RoseTTAFold2-PPI and D-Script on the experimentally supported/unsupported and complex-membership benchmarks, restricted to the subset of protein pairs that passed the CM2.0 coevolutionary filters and were assigned an IntScore. (XLSX)

S5 Table AUC values for CM2.0, RoseTTAFold2-PPI and D-Script on the experimentally supported/unsupported and complex-membership benchmarks using all queried protein pairs. Pairs removed by the CM2.0 coevolutionary filter were assigned IntScore = 0 solely for this end-to-end benchmark.
(XLSX)

S6 Table Numbers of experimentally supported and unsupported protein pairs retained and filtered by the CM2.0 coevolutionary filtering stage in each benchmark system, together with Fisher’s exact test odds ratios and associated p-values. (XLSX)

S7 Table Summary of predictions included in communities extracted automatically from the union of the predicted interactomes of *E. coli* and *S. enterica* with three interaction score thresholds (S9–S11 Figs). GT stands for ground truth and IntScore is the interaction score from ContextMirror2.0.
(XLSX)

S8 Table Computational time and resource allocation for ContextMirror2.0. (XLSX)

S9 Table PPI comparative metrics across reference proteomes of *E. coli, S. enterica,* and *S. aureus.* (XLSX)

S10 Table Calculated chance precision metrics for predicted PPIs in *E. coli, S. enterica,* and *S. aureus.* (XLSX)

S11 Table Summary of protein complexes predicted using AlphaFold Multimer v2.3, with CM2.0 scores and pDockQ2 quality metrics. The table presents organisms, their associated complexes, CM2.0 scores, true positive (TP) validation status, and the minimum pDockQ2 scores for each complex. The pDockQ2 metric evaluates the quality of individual interfaces within a multimer, with the lowest pDockQ2 score for each complex shown. The TP column indicates either “NO” or the name of the source where validation was obtained. Rows in bold highlight complexes with a minimum pDockQ2 score exceeding 0.23, indicating higher interface quality.
(XLSX)

S12 Table Reciprocal best hits (RBH) considering the top 5 homolog results for each potential comparison. n indicates the size of each proteome. (XLSX)

S13 Table Protein pairs included in the complex benchmarking analyses. Each row corresponds to one protein pair evaluated in the benchmark datasets. Pairs are classified according to experimental support and whether they represent same-complex or complex-control pairs. The CM2.0 IntScore is reported for pairs retained after the initial coevolutionary filtering step; pairs excluded during filtering are indicated as “filtered out.”
(XLSX)
