# Supplementary material for: Sequence-based coevolutionary prediction of species-specific interactomes
Source: PLoS Comput Biol. 2026 Nov 6;22(11):e1014839. doi: 10.1371/journal.pcbi.1014839 (PMC13742926; doi:10.1371/journal.pcbi.1014839)
Supplement: Supplementary file 1 [file pcbi.1014839.s031.pdf]

We thank the reviewers and the editor for their thoughtful and constructive feedback. The central concern across the reviews is the systematic validation of CM2.0. We agree that benchmarking is essential, and in the revised manuscript we expanded our evaluation evaluation following the reference datasets introduced by Cong et al. (2019) and the benchmarking principles emphasized by Zhang et al. (2025), which together outline the main criteria for rigorous assessment of proteome-wide PPI predictors:

- **Positive-recovery benchmarks**. Ranking all predictions across the proteome, CM2.0 recovers ≈14-fold more known positives than expected by chance. For this analysis, the set of positives was taken from the same datasets used in Cong et al. (2019) (EcoCyc, AP-MS, Y2H), and the results are consistent with the ≈8.5 fold enrichment reported in our initial benchmarks

- **Benchmarks with explicit negatives.** On the PDB dataset used by Cong et al., CM2.0 reaches ≈0.03 precision versus a 0.002 baseline (≈10× enrichment). On BioGRID physical PPIs with random negatives, CM2.0 achieves ≈0.89 precision and AUPRC=0.77 in the balanced 1:1 setup (baseline 0.50), and ≈0.009 precision in the ≈1:400 setup (baseline 0.0025), corresponding to a ≈3.7× lift. The use of positives from BioGRID combined with random negatives is a standard procedure in benchmarking PPI prediction tools (as in D-Script), and it allows comparison with recent supervised approaches. These results illustrate how absolute precision drops as prevalence decreases, but enrichment above random is maintained even under extreme imbalance.

We believe that these analyses demonstrate both the limits and the strengths of CM2.0. The method is not designed for residue-level binding prediction but for large-scale discovery of functional associations. Accordingly, while it is not directly comparable to residue-level predictors, CM2.0 consistently enriches for true associations above chance across independent datasets. Conceptually, CM2.0 should be viewed not as a predictor of non-interactions, but as a tool that prioritizes pairs more likely to interact: it ranks pairs by how likely they are to interact.

We did not re-run Cong et al. (2019). As detailed in *Supplementary Methods M6.2* and *M7.1* of Cong et al. (2019), they describe a complex pipeline in which, for each protein pair, multiple residue-residue coevolutionary models (MI, DCA, GREMLIN, and GPU-based CCMPRED) are run, followed by exhaustive docking with 300,000 poses per pair. They do not report execution times or provide a readily available implementation, and **reproducing their pipeline would require reconstructing the full computational infrastructure used in 2019**, making a full re-run impractical both in terms of computational resources and reproducibility. More importantly, Cong et al. (2019)'s approach and CM2.0 are designed for different purposes: their method targets residue-level docking of physical binding interactions, while CM2.0 focuses on proteome-wide coevolutionary associations. For this reason, we consider the most appropriate comparison to be the evaluation of CM2.0 on the same curated datasets used by Cong et al. (2019) (PDB, EcoCyc, Y2H, AP-MS). To further ensure fairness and in line with the concerns raised by Zhang et al. (2025) in their recent review, we also included BioGRID-based benchmarks under both balanced and imbalanced scenarios.

Reviewers' comments:

Reviewer's Responses to Questions
Comments to the Authors:
Please note here if the review is uploaded as an attachment.

Reviewer #1:
In this paper the authors describe an updated version of their tool ContextMirror to version 2.0 which predicts PPI-interactions. They apply it to three bacterial genomes and identify a number of previously unannotated PPI-networks. The method is based on phylogenetic similarity of potentially interacting proteins across a number of specied.

What is severely missing in this paper is a proper benchmarking study. The authors use a positive set of proteins from : IntAct, STRING (score >0.2), EcoCyc and Interactome3D. Here it is unclear how STRING is used, as it is both a collection of experimental information and a prediction of interacting proteins using various method (including methods that are not that different from ContectMirror), so it can be asked if this is a suitable set of positive interactions. From what I understand all pairs not present in these databases are assumed to be negative - although this is certainly now true - it is likely an OK assumption. However the authors to need to answer how does the performance vary with a different set of positives. There are also large scale PPI studies (yeast2hybrid, tap-tag etc) primarily for yeast that can be used to benchmark the methods, Once a good set of "true" interactions are made - the authors do need to compare their method with state of the art methods, for instance RoseTTAfold-PPI is fast enough to run on all pairs, but you could also consider STRING (or Funcoup) as alternative predictors. There are also other methods (which perform badly in general) such as D-script that could be included.

We appreciate the reviewer's emphasis on benchmarking, and we have expanded this aspect of the manuscript in the revision. Below we clarify our original choices and describe the new analyses added in response.

In the revised manuscript we clarify that STRING was used only as a source of positives (confidence >0.2), together with IntAct, EcoCyc and Interactome3D, to maximize proteome-wide coverage. This design matches the intended scope of CM2.0, which predicts broad functional associations, not just residue-level physical contacts.

To complement our original evaluation, we tested CM2.0 on the benchmarking sets introduced by Cong et al. (PDB positives/negatives; EcoCyc, AP-MS, Y2H positives). On the PDB benchmark, CM2.0 achieved more than a tenfold enrichment over random expectation (≈0.2% baseline). Our original evaluation was a positive-recovery benchmark: all proteome-wide predictions are ranked, and enrichment of known positives is measured. We

repeated the positive-recovery evaluation using the curated EcoCyc, AP-MS, and Y2H datasets from Cong et al. (2019) and found that CM2.0 enriched for true interactions approximately 14-fold above chance, consistent with the signal observed in our original benchmarks. This indicates that the positive-recovery performance is robust to the choice of positive reference set.

In addition, to align with current practice in benchmarking PPI prediction tools (e.g. D-Script) and the issues pointed out in Zhang et al. (2025), we introduced new BioGRID-based tests with explicit negatives generated at different ratios. In the balanced 1:1 setup, CM2.0 reached ≈0.89 precision and an AUPRC of 0.77 (baseline 0.50). In the imbalanced ≈1:400 setup, absolute precision dropped to ≈0.009, as expected mathematically, but the model still achieved a ≈3.7× enrichment over random (baseline 0.0025).

We believe that these analyses demonstrate both the limits and the strengths of CM2.0. It is not competitive with residue-level predictors on physical binding benchmarks, but it is not a random classifier: across independent datasets, CM2.0 consistently recovers more true positives than expected by chance, independent of class imbalance. Conceptually, CM2.0 should be viewed not as a tool for ruling out non-interactions, but as a prioritization method for pairs that are more likely to interact. In other words, its strength lies in enriching true associations above random expectation, rather than estimating the probability that two proteins do not interact.

We added these clarifications in the Discussion section (lines 410-424).

Another question is if this method is useful beyond bacterial genomes. How does it apply to yeast, which is not that much bigger so it should be possible to run it. I would assume that the existence of paralogs might make it less accurate though.

We thank the reviewer for raising this point. The current study focused on bacterial genomes because CM2.0 is particularly well-suited to organisms with relatively low paralog redundancy and where experimental PPI coverage remains sparse. In principle, CM2.0 is fully scalable to eukaryotic genomes, including yeast, as the algorithm operates at the whole-proteome level and computational cost is not prohibitive for proteomes of this size.

A systematic application of CM2.0 to yeast (or other eukaryotes) is feasible, but beyond the scope of this manuscript, which was designed to demonstrate performance and scalability in prokaryotic systems. We see extending the method to eukaryotic proteomes as a natural next step, particularly in organisms like yeast that have both rich experimental PPI data for benchmarking and a moderate level of paralogy.

It should also be noted that the performance is actually really bad (10% recall at 30% precision). Perhaps the precision can be improved by using AlphaFold2 (or 3) to predict the interactions - this should filter away some FPs, I think.

We thank the reviewer for their comment. About the performance, it should be noted that while in other domains a performance like this would be considered bad, in the task of PPI prediction where the search space increases quadratically with the number of proteins in a given proteome, the performance of sequence-based methods is usually not so high (see

precision-recall curves in Cong et al.'s work before applying structural methods as an example). This is because of the difficulty of the task and the huge size of the search space (millions of possible PPI pairs). Another issue that arises when measuring performance is regarding the false positives, since each "false positive" might be an actual false positive or it can be an yet-to-be-discovered PPI that was correctly identified by the prediction method. In conclusion, given that performance metrics such as recall and precision should be looked at differently in PPI prediction tasks, the results achieved by CM are not worse than the results reported in similar works such as Cong et al.'s.
About the reviewers suggestion: given the recent progress achieved in protein structural prediction, we see how it might be tempting to use tools such as AlphaFold for the prediction of PPIs. However, in the AF3 paper the authors emphasize that the model still has many limitations for protein complex prediction, including "a tendency of the model to occasionally produce overlapping (clashing) atoms". We predicted the structure of 30 putative protein-protein pairs with AF and there were many stereochemical violations even in the cases that were true positives, i.e. known PPI pairs. This small test shows that the model is clearly not fit for PPI prediction. Additionally, adding a 3D modelling step to ContextMirror would highly increase the computational time required for the predictions, which is something we are trying to avoid by sticking to primary sequence information.

I looked at the code and it looks OK (but it is not good practice to include _pycache__ and other temporary directoris in the git repository, so a small cleanup would be good.

Thank you for noticing it. We removed the _pychache_ directories to make the repository cleaner.

Reviewer #2:
The author introduces ContextMirror 2.0 (CM2.0), a co-evolution analysis framework that is entirely sequence-dependent, unsupervised, and capable of large-scale implementation for predicting protein-protein interaction at the species level. This framework was used to compare conserved and species-specific interactions among three bacteria: E. coli, S. enterica, and S. aureus.

1. The current paper's format has significant room for improvement. Lines 39-67 focus solely on discussing the CM2.0 tool. In accordance with standard academic writing format, the introduction should address why this research is being conducted, the challenges present in the current field, an overview of related work, and a discussion of related work. These elements are currently missing from the introduction and should be rewritten. It is recommended that the author categorize related work by introducing traditional predictive PPI algorithms and recent developments, such as 10.1109/TCBB.2022.3196336 and 10.1109/JBHI.2024.3357979.
We thank the reviewer for this constructive feedback regarding the introduction structure and fully agree with the comment. We have substantially revised the introduction to follow a standard academic writing format by adding a new second paragraph and editing the third paragraph, which address traditional predictive PPI algorithms and recent developments, including the reference 10.1109/TCBB.2022.3196336. We have decided not to include the reference 10.1109/JBHI.2024.3357979, as its focus is highly specific and does not align directly with the scope of our study. We have also modified the rest of the introduction text to accommodate the new paragraph, mostly by repositioning content from the previous version

or reducing text that directly reported CM2.0 results, focusing instead on contextualizing our work within the broader field and clearly articulating the research gaps we address. The revised introduction now properly establishes the research context, identifies current limitations in the field, categorizes existing approaches, and clearly motivates our biological research objectives before presenting our specific contributions. All modifications are highlighted in red, including both changes in wording and changes in position.

2. The author mentions that, for a given genome, the top 1000 predictions achieve approximately 40% precision. I would like to understand what this number implies compared to other tools. How can the remaining 60% of PPI be validated, considering that these predictions are made but not found in the database?

We thank the reviewer for their insightful comment. Without experimental essays, it would be difficult to validate the remaining PPIs predicted by CM2.0. We actually investigated whether structural methods such as AlphaFold (AF) could be useful for validating the results in section 2.1 but found out that AF predictions are not reliable enough for PPI prediction. In practice, we see CM2.0 as a prioritization tool: it enriches for true interactions above random expectation, and the top-ranked predictions provide a tractable shortlist for experimental follow-up. Future work could explore integrating CM2.0 with emerging structure- and sequence-based frameworks (e.g., Boltz-2 or AlphaFold variants) to assess whether such hybrid approaches can help validate or refine the predicted interaction networks.

3. I am curious whether the author has evaluated the runtime of CM2 or compared it with similar tools, as this tool involves extensive phylogenetic tree computations, such as sequence alignment and profile alignment, which are relatively time-consuming calculations. I only saw the time of CM2 in Table S7.

We thank the reviewer for their comment. We agree that phylogenetic trees computation is computationally costly and we address this problem by using the fasttree algorithm to compute the trees which is faster than other methods. We did not run any other methods locally given the difficulty to find a method that would be a fair comparison to CM. Methods that rely on structural information (e.g. RoseTTAFold-PPI) are not comparable. Supervised ML methods (e.g. D-Script) are also not comparable and these two categories comprise most of the existing methods for PPI prediction. The only coevolution-based method of similar spirit is Cong et al. (2019), but their pipeline is not publicly available and would in any case be infeasible to reproduce, as it involves multiple residue-residue coevolution models (MI, DCA, GREMLIN/CCMPRED) followed by exhaustive docking with ~300,000 poses per pair (See Cong et al. (2019) - *Supplementary Methods M6.2* and *M7.1*). For these reasons we limited our analysis to reporting CM2.0's own runtime (Table S7) and clarifying the design choices that make it computationally tractable at proteome scale. We clarified these reasons in the Discussion (lines 442-452).

4. The author should address in Section discussion the tool's performance in certain protein families where public databases lack a sufficient number of shared orthologs, making it unable to meet the threshold of "N common species."

Thank you for raising this issue. If there are not enough common species to meet the threshold (e.g. 1000), then the pair of proteins is not considered in the analysis, since the

coevolutionary relationship between both proteins is not strong enough. The “N common species” threshold is a parameter that can be set by the user, so for species with less coverage, a lowering of the threshold is indicated. We added this clarification in the discussion (lines 453-459).

5. Regarding future work, please discuss the possibility of applying advanced graph neural networks (10.1109/JBHI.2024.3357979), that are developed to learn sequence embeddings, for improved performance of PPI prediction.

We thank the reviewer for this suggestion. We respectfully note, however, that the referenced work (Li et al., 2024, DOI: 10.1109/JBHI.2024.3357979) addresses the identification of RNA N6-methyladenosine (m6A) modification sites using a graph contrastive clustering framework. While the methodology is interesting within the RNA modification field, it is not directly aligned with our focus on coevolutionary methods for protein–protein interaction prediction. To acknowledge the reviewer’s broader point, we have already incorporated one of the suggested citations (10.1109/TCBB.2022.3196336) into the revised Introduction, as it more directly relates to predictive algorithms for PPIs.

In addition, we have added a general remark in the Discussion to recognize the potential of advanced machine learning approaches (lines 468-470):

“*Future developments may also benefit from deep learning strategies such as graph neural networks for sequence representation learning, which could provide complementary perspectives to coevolution-based approaches.*”

We believe this appropriately acknowledges the reviewer’s comment while keeping the focus of our paper consistent with our methodology.

Reviewer #3:

Summary:
The authors introduce a new method, ContextMirror2.0. It predicts the protein-protein interactions (PPIs) of a query proteome by quantifying the co-evolution of protein pairs. The approach evaluates all possible protein pairs (p, q), provided both proteins have a sufficient number of orthologs in other species. Co-evolution is quantified by correlating the similarity of MSA-derived phylogenetic trees. The resulting score is adjusted by accounting for the fact that proteins do not interact in isolation; thus, the co-evolution of a pair should also be reflected in third proteins. Protein pairs are then ranked based on this score, and those above a certain threshold are predicted to interact, either physically or functionally.
The authors demonstrate the usefulness of their method by predicting all PPIs for E. coli, S. enterica (as well-characterized, Gram-negative examples), and S. aureus (as a poorly characterized, Gram-positive example). By modeling their best predictions with AlphaFold Multimer, they show that their method mostly predicts non-direct interactions. They further merged the predicted networks of the three species by identifying close homologs to identify

functional communities preserved across species. Within these communities, the method enables hypothesis generation about the function and divergence of uncharacterized proteins.

The strengths of the method are that it does not need (the sparsely available) negative examples, that it is interpretable, rather fast (compared to deep learning approaches), and enables network community detection for functional analysis. The drawbacks of the method are that it is probably prone to false positives and does not distinguish between physical and non-physical, functional interactions (which could, depending on the application, also be an advantage). Overall, the research is novel, of high quality, and useful to the field. I strongly encourage publication after addressing the following points:

Major Points:

1. Methods: The methods section is at times difficult to follow, particularly regarding which steps are performed per protein and which are performed per protein pair. This could be greatly improved by including pseudocode or using mathematical notation. For example:
(1) For each protein p, we identify a set of orthologs O_p using RBH.
(2) For each possible protein pair (p,q) in the proteome of the query species, we construct O_{pq} = O_p \intersect O_q.
(3) If |O_{pq}| >= 1000, we calculate two MSEs MSE_p, MSE_q against all ortholog sequences of O_{pq}
… and so on. Please consider revising the methods accordingly, as this is central to the paper's clarity.

Thank you for this helpful suggestion. We agree that this distinction is central to understanding our pipeline. In response, we revised the Methods (Section 4.1) to make this distinction explicit. Specifically:

- We clarified that ortholog sets O_p are computed per protein (p) using RBH in line 493.
- We introduced mathematical notation to describe how shared species are identified for each protein pair in lines 495-498:

$$O_{pq} = O_p \cap O_q$$

- We revised the description of Steps 1–3 using consistent variable names across the method (e.g., r'_{pq}, $\rho'_{pq.k}$).
- All formulas now use standardized notation to clarify which quantities are computed from distance matrices, profile vectors, or partial correlations.

We opted for concise mathematical descriptions that directly align with the figure and implementation details and hope this clarifications improve the clarity and accessibility of the Methods section

2. Methods: Please also revise the figure of the ContextMirror2.0 pipeline (Fig. 7) for better clarity:
2.1. Change "Multi-FASTA protein" to, e.g., "Multi-FASTA file of the species proteome".
2.2. Show that the FASTA input consists of proteins A, B, C, D, possibly using color coding.
2.3. Make it visually clear that the next steps operate on pairs of proteins. Currently, it appears the steps are for a single protein. Highlight, for instance, that you're working with (A, D) in subsequent panels.
2.4. Indicate explicitly that each dot in the tree-tree correlation plots corresponds to a specific protein pair, and annotate accordingly (e.g., "(A, D)").
2.5. Label matrices with their names (e.g., r for the tree similarity matrix) for easier reference.

We thank the reviewer for the suggestion. Since we detailed the mathematical calculations more thoroughly in the revised Methods section, we also updated Figure 7 accordingly. As per your suggestion, the input file is now indicated as "MULTIFASTA PROTEOME," and proteins A, B, C and D are explicitly shown. Additionally, we clarified that the steps are performed for pairs of proteins (A-D in the case of the figure). The Pearson correlation dots you referred to were originally a visual representation of the calculation of a linear correlation coefficient. However, to improve clarity, we removed these icons and replaced them with the appropriate mathematical notation indicated in the Methods section (rAD, r'AD and ρ'AD.X , for tree-tree correlation, profile-profile correlation and partial correlation respectively). Finally, we added explicit mathematical notation to the matrices in the figure:

$R^{tree}: tree - tree\ correlation\ matrix$

$R^{profile}: profile - profile\ correlation\ matrix$

$R^{partial}: partial\ correlation\ correlation\ matrix$

3. Methods/Figure 1: I am unsure about the calculation of the random precision, more precisely, about the denominator. You currently calculate the probability of obtaining the TPs by chance among all possible edges. However, your method filters out edges that lack sufficient ortholog coverage (>1000). Shouldn't the denominator only include pairs that meet this criterion? Please clarify or correct this if needed.
We thank the reviewer for this comment and confirm that the random precision is indeed calculated as suggested: the denominator includes only the protein pairs that CM2.0 is able to evaluate (i.e., those with ≥1000 shared orthologs). In other words, pairs filtered out due to insufficient coverage are not part of the denominator, as they are not considered in the prediction space. The apparent discrepancy arose from a numerical inconsistency in Supplementary Table S4, where the number of true positives was miscounted, leading to slightly underestimated random baselines. The corrected values are now 4.9% for E. coli, 4.0% for S. enterica, and 3.9% for S. aureus. This correction does not affect CM2.0's predictions or conclusions but ensures that the reported chance precision matches the conceptual definition already implemented in the method.

4. Methods: You state that your interaction score is bounded between 0 and 2. I don't know if this is true. If n>6, -0.1+0.02(n-1) is >0, which makes the score grow with the number of third interactors. If we had a perfect correlation of 1 for PPcorr_{AB} and 100 perfectly correlated third interactors (+1/100*100=1), we would get 1+1-0.1+0.02*99=3.88. If they were perfectly

negatively correlated, we would get -1-1-0.1+0.02*99=-0.12. Am I misunderstanding something here? If so, please make this clearer in the formula or surrounding text.

Thank you for raising this important point. You are correct: the formula previously included in the manuscript implied a correction term of the form −0.1+0.02(n−1), which would indeed allow the interaction score (IS) to grow unbounded with increasing numbers of third interactors. In practice, however, the implementation used in all analyses, as reflected in our GitHub repository, applies a bounded penalty function that **only affects interactions influenced by ≤50** third proteins. The penalty decreases linearly from 0.1 (at n=1) to 0.002 (at n=50), and is zero beyond that. This approach penalizes under-supported interactions while preventing score inflation when many third proteins are present.

We have updated the formula in the Methods section (see below):

$$IntScore_{pq} = r'_{pq} + n^{-1} \sum_{i=1}^{n} \rho'_{pq.i} - \delta(n)$$

With the penalty term $\delta(n)$ defined as:

$$\delta(n) = \begin{cases} 0.1 - (0.1 - 0.002) \cdot \frac{n-1}{49}, & \text{if} n \leq 50 \\ 0, & \text{if} n > 50 \end{cases}$$

Thus, in your scenario:

- r'_{pq} = 1
- 100 third interactors, all perfectly correlated ⇒ average partial correlation = 1
- n=100 > 50 → $\delta(n)$ = 0

The final interaction score becomes:

$$IntScore_{pq} = 1\ (r'_{pq}) + 1\ (\sum_{i=1}^{n} \rho'_{pq.i}) - 0\ (\delta) = 2$$

$$IntScore_{pq} = 1 + 1 - 0 = 2$$

This is now the maximum score possible under the implemented logic. We have updated the Methods section with the correct formula and clarified that scores remain within a practical range close to [0, 2] due to the underlying correlation metrics (lines 615-618).

5. Results: Did you re-run the method from Cong et al. for your comparisons? Since their work dates back to 2019, rerunning it with current datasets could yield different results. Also,

did you compare run times from their reported values or based on your own benchmarking setup? Please clarify and, if not already done, consider re-running Cong et al. for a fair comparison.

As explained in our response to Reviewer #2 (point 3), Cong et al.'s (2019) method is not directly comparable to CM2.0. Their pipeline targets residue-level docking of physical contacts and requires extensive coevolution and docking computations that are not publicly available or feasible to reproduce. Because of these fundamental differences in scope and scale, re-running their approach or comparing runtimes would not be meaningful. CM2.0's own runtime is reported in Table S7.

To provide a fair basis of comparison, we applied CM2.0 to the same benchmarking sets that Cong et al. used in their study (PDB positives/negatives; EcoCyc, Y2H, and AP-MS positives). On the PDB set, CM2.0 achieved ≈3% precision versus a 0.2% random baseline (≈10× enrichment). This modest absolute precision is not unexpected, since the PDB benchmark focuses on residue-level binding contacts, whereas CM2.0 captures broader coevolutionary associations that do not always correspond to physical binding events. When using the EcoCyc, Y2H, and AP-MS collections instead, CM2.0 consistently recovered many more positives than chance (≈14-fold enrichment), showing that the positive-recovery signal is reproducible across independent sources. In addition, and following current practice in PPI benchmarking (e.g. D-Script), we included new BioGRID-based tests with random negatives at both balanced (1:1) and highly imbalanced (≈1:400) ratios. These benchmarks confirm that, although absolute precision decreases as prevalence drops, CM2.0 consistently enriches true positives above chance across different scenarios of class imbalance. Taken together, these complementary benchmarks highlight both the limitations of CM2.0 in residue-level binding tasks and its strength as a scalable, proteome-wide discovery tool.

6. Results: You appear to model only single copies of each protein. However, many protein complexes include multiple copies of the same subunit. If I understand correctly, your approach could capture this. If so, this is an important capability and should be discussed in the manuscript, especially given that many PPIs form complexes involving multiple copies of the same proteins, cofactors ,or ligands.
We thank the reviewer for this comment. CM2.0 was not designed to handle self-interactions since it is based on phylogenetic tree similarities (the similarity of a tree to itself would always be 1), hence the discussion you are suggesting is not present and would be out of the scope of our paper.

7. Availability: I cannot access your GitHub, I think it might be private. Please change it to public.

Thank you for raising this concern, we revised the repositories and the code is available at: https://github.com/bsc-life/ContextMirror2.0

Minor Points
1. Consider making Figure 7 your new Figure 1, as it outlines the core methodological contribution. You might also incorporate the interaction score formula in this figure to fully represent your pipeline visually.

We appreciate the reviewer's suggestion regarding Figure 7. However, we respectfully maintain our current figure organization as it better aligns with our paper's primary contribution and scope.

While we acknowledge that Figure 7 effectively illustrates our methodological pipeline, **our main contribution lies in the biological insights and results derived from applying this methodology**, rather than the computational pipeline itself. The pipeline serves as an analytical tool to uncover meaningful biological relationships, but it is not our central result.

We believe maintaining the current figure order better serves readers by immediately highlighting the biological significance of our work, which aligns with the journal's audience and our research objectives.

2. Results 2.1: The sentence: "…CM2.0 tends to consistently assign high scores to true positive PPIs […] with an accuracy […] comparable to other similar methods" could be improved:
2.1. "tends to consistently" contradicts itself. I would suggest to strike the consistently as you only recover ~20% of the ground truth set with a precision of ~20%.
2.2. As you compare only to Cong et al., please revise "other similar methods" to "Cong et al." for precision.

We thank the reviewer for pointing this out. We have revised the sentence in Section 2 (Results, 2.1) accordingly. Specifically, we removed the word "consistently" to avoid the contradiction with the reported performance values, and replaced "other similar methods" with "Cong et al." for accuracy and clarity. The revised sentence now reads:

"…with an accuracy over ten times higher than what would be expected by chance and comparable to Cong et al. state-of-the-art method"

3. Results 2.3: The transition from "three electron transfer flavoproteins are predicted to interact with yibF and rfbI" to "this suggests host-pathogen interactions and oxidative stress in S. enterica" happens too abruptly for me. As a bioinformatician with not much knowledge of bacterial pathways, I found this reasoning hard to follow. Please provide additional context or references to help readers understand the biological rationale.

Thank you for pointing this out. We agree that the biological rationale could be expanded to better support the conclusions, particularly for readers less familiar with bacterial physiology. We have revised this section to clarify that:

- RfbI is involved in O-antigen modification, a key component of LPS biosynthesis linked to host immune evasion and virulence in Gram-negative bacteria.

- YibF is a glutathione S-transferase, part of a well-known family involved in redox balance and oxidative stress response.

We added some explanations and included relevant references in lines 277-282.

4. Methods: In your interaction score, you write PPcorr_{AB}, but before, you term this matrix r'. For consistency, please update to r'_{AB}. Also, you sum from i=1 to n, but i does not occur.

Thank you for pointing this out. We have revised the formula to maintain consistent notation throughout the Methods section. The term previously labeled as $PPcorr_{AB}$ has been updated to r'_{pq}, consistent with the profile–profile correlation matrix defined in the previous step.

Additionally, the summation has been rewritten to explicitly reflect the indexed partial correlation terms by introducing $\sum_{i=1}^{n} \rho'_{pq.i}$ instead of the former $\sum_{i=1}^{n} (\rho'_{AB.R} + \rho'_{AB.Q} + ... + \rho'_{AB.N})$.

5. Methods: Why are there three different p-value cut-offs (10^-5 for r, 10^-11 for r', 10^-6 for rho)? Why is only r' corrected for multiple testing? Please justify the differences in thresholds and correction procedures, or adapt for consistency.

We thank the reviewer for this important observation. The significance thresholds for the tree-tree correlations (r) and the partial correlations (ρ') are inherited directly from the original ContextMirror implementation (Juan et al., PNAS 2008), where values of $p \leq 10^{-5}$ and $p \leq 10^{-6}$, for r and ρ respectively, were established and shown to provide good control of false positives. We retained these thresholds in order to ensure comparability with the original method. However, for the profile-profile correlations (r'), our implementation required an adjustment. In Juan et al. (2008), homologs were searched within a set of 116 genomes and pairs with as few as 15 species in common. In our case, we use the full non-redundant database and require at least 1,000 species in common. This results in much longer coevolutionary profiles (vectors) for each protein. Statistically, the increased dimensionality translates into many more degrees of freedom, which drives the p-values to be extremely small even for weak correlations. We verified empirically that with a raw cut-off of $p \leq 10^{-5}$, essentially all correlations passed and the filter became uninformative.

To address this, we applied a Bonferroni correction in the profile-profile step to account for the very large number of simultaneous comparisons. This resulted in a more stringent threshold of $p \leq 10^{-11}$, which effectively filters out the weaker/noisy correlations and retains only the most robust signals. Importantly, this modification improves control of false positives in our larger-scale implementation while keeping the spirit of the original method.

We have clarified this rationale in the revised Methods section (lines 513, 534-542 and 566).

6. Supplement, Figure S2: The x-axis label "Min Nf" is unclear. Please explain this in the figure caption.

We thank the reviewer for the observation. As stated in the Methods section (see 4.2):“*After calculating N_{eff} for MSA A and MSA B in a given paired MSA, we defined the N_{eff} of the paired MSA as the minimum between N_{eff} A and N_{eff} B.*”. We now included this clarification in the caption of Figure S2.

7. Methods: You currently refer to the interaction score as “IS,” which may carry unintended associations. Consider renaming it to something IntScore_{AB}? score_{AB}? CM2.0 score_{AB}?

Thank you for the suggestion. To avoid ambiguity and improve clarity, we have renamed the interaction score throughout the manuscript from “IS” to IntScore (e.g. $IntScore_{pq}$).

Reviewer #5:
The study introduces ContextMirror2.0 (CM2.0), a sequence-based coevolutionary method for predicting species-specific protein-protein interactions (PPIs), aiming to overcome limitations of previous computational and experimental approaches. Recent DL and ML tools have revolutionized PPIs prediction; however, predicting protein-protein or protein-ligand interactions without any prior knowledge remains a challenge for the scientific community. Achieving 40% precision in top predictions is considered strong for PPI prediction, as in realistic proteome-wide PPI datasets, interacting protein pairs are extremely rare compared to all possible pairs. Overall, this is a clear, concise, and well-written manuscript. I find the research topic interesting and relevant to PLOS Comp Bio. The simulation results are easy to follow, and the manuscript mostly describes methods used, presented figures, and structural details. The results achieved are well-discussed in the main body of the reported manuscript. There are a few points that should be addressed:

When tested with AlphaFold, only 3 out of 30 high-scoring predicted interactions produced structurally acceptable models. Is there a reason for AlphaFold's failure other than no experimental data to predict these interactions, or does this suggest that high coevolutionary scores do not always indicate physically plausible complexes?
We appreciate the reviewer’s question. Indeed, the limited success rate in AlphaFold modeling does not necessarily imply that high coevolutionary scores are incorrect. Several factors may explain the observed outcome: (1) AlphaFold performs best when experimental templates or strong evolutionary constraints are available. For many of the tested protein pairs, such supporting information is lacking, which likely hampers AlphaFold’s ability to generate plausible structural models. (2) Coevolutionary scores capture correlated mutations that suggest potential functional or physical interactions, but they do not guarantee that these interactions are structurally stable or detectable at the resolution required by AlphaFold.

We believe this point is already clarified in the 7th paragraph of Section 2.1, where we emphasize that translating coevolutionary signals into structural models remains challenging and that complementary methods are needed for validation. The relevant passage reads: “*These results highlight the limitations of AF Multimer in predicting PPIs, particularly when the interactions are not of a physical type. In such cases, additional aspects need to be considered, as covered by CM2.0. However, AF Multimer could still be useful for making*

distinctions among interactions, given that 3 out of 17 true positive interactions tested did surpass the pDockQ2 threshold". We hope this clarification addresses the reviewer's concern and that they are satisfied with our response.

Line#141-142: "…3 out of 17 true positive interactions tested did surpass the pDockQ2 threshold." This statement is not clear to me and needs elaboration on AF distinctions.
We thank the reviewer for pointing this out. The statement refers to the use of the pDockQ2 score to assess model quality. As described in the Methods section, a score above 0.23 is considered the threshold for acceptable structural models, as proposed by the developers of pDockQ2 (Zhu et al. 2023 - reference 8). In our results, 3 out of the 17 true positive interactions tested exceeded this threshold, indicating that AlphaFold Multimer was able to produce acceptable-quality structural models for a subset of the interactions. We have revised the text in Section 2.1 to make this point explicit.

The revised sentence now reads:
"…However, AF Multimer could still be useful for distinguishing among interactions, given that 3 out of 17 true positive interactions tested achieved a pDockQ2 score above the 0.23 threshold *for acceptable model quality, as proposed by the developers of the method*."

We have also improved clarity in the Discussion section accordingly:
[...] For instance, although a S. aureus PPI received a high CM2.0 interaction score and was validated in experimental databases, the AF-predicted complex had a very low pDockQ2, suggesting that the proteins are functionally related but do not form a stable physical interaction.

We hope these two clarifications address the reviewer's concern.
