# Supplementary material for: Sequence-based coevolutionary prediction of species-specific interactomes
Source: PLoS Comput Biol. 2026 Nov 6;22(11):e1014839. doi: 10.1371/journal.pcbi.1014839 (PMC13742926; doi:10.1371/journal.pcbi.1014839)
Supplement: Supplementary file 2 [file pcbi.1014839.s032.pdf]

Dear Editor,

We thank you and the reviewers for the careful evaluation of the revised manuscript. While we understand the basis of your decision, we would like to, respectfully, ask you to reconsider it on the basis of the three main arguments below.

Sincerely,

Alfonso Valencia

1.- The reports of Reviewer 2 and 5 are completely positive, Reviewer 3 comments are mainly easily addressable clarifications. Regarding Reviewer 4, this reviewer's comments were not included in the original decision letter, so we couldn't address them. We will be happy to respond to any specific concerns should they be communicated to us.

2.- Regarding Reviewer 1's concerns about not having addressed his comments, we want to clarify that we did answer all the comments and, accordingly, made substantial changes in the main document. We hope that the misunderstanding is now clarified in the response below.

3.- Along this line, a particularly important point to clarify is that the scope of our CM2.0 method is essentially different from the other systems, including structure-based predictors such as RoseTTAFold-PPI. Our proposal targets species-specific functional associations using unsupervised evolutionary signals, rather than residue-level physical binding interactions. Therefore, a full proteome-wide head-to-head comparison against docking/structure-based pipelines is not appropriate. Additionally, the structural methods are very expensive in computational time, making the full comparison unfeasible in practice. Instead, we have focused on validation strategies that are standard in the field and meaningful for these large-scale unsupervised PPI predictions.

Reviewer #1:

I really have no comments as the authors basically did not do anything regarding my raised concerns, so I leave it up to the editor to evaluate if my concerns were important or not.

We thank the reviewer for their careful reading of the manuscript and for the comments provided in the first round of review, and regret that our revisions may not have fully met the reviewer's expectations. We would like to clarify that all points raised in the first review were carefully considered and led to noticeable adjustments in the revised manuscript.

In direct response to the reviewer's comments, we (i) clarified the use of STRING as a source of true positive functional associations, (ii) evaluated performance across multiple independent positive reference sets (EcoCyc, AP-MS, Y2H, PDB) and (iii) introduced explicit negative benchmarks under both balanced and strongly imbalanced conditions, assessing performance as enrichment over random expectation following standard practice (e.g.

D-Script). These changes were introduced to address the concerns about benchmarking rigor, choice of positives, and interpretation of performance.

Some suggested analyses, such as systematic proteome-wide comparison against structure-based predictors (e.g. RoseTTAFold-PPI or AlphaFold-based complex prediction) or re-running the pipeline described in Cong et al. (2019), would address a different prediction task (residue-level physical binding rather than large-scale functional association) and would also be computationally infeasible at the scale considered here (exhaustive proteome-wide docking), particularly given that this study predicts complete interactomes for three species. For clarity, and as explained in detail in our first-round rebuttal, we reiterate here why we did not perform such comparisons.

"We did not run other tools locally because it is difficult to define a fair comparison for CM2.0. Methods that rely on structural information (e.g. RoseTTAFold-PPI) are not comparable. Supervised ML methods (e.g. D-Script) are also not comparable, and these two categories comprise most of the existing methods for PPI prediction. The only coevolution-based approach of comparable spirit is Cong et al. (2019), but their full pipeline is not publicly available and would be infeasible to reproduce at proteome scale, as it involves multiple residue-residue coevolution models (MI, DCA, GREMLIN/CCMPRED) followed by exhaustive docking (~300,000 poses per pair; see Cong et al., 2019, Supplementary Methods M6.2 and M7.1).

For these reasons, we focused on evaluating CM2.0 on the same curated datasets used by Cong et al. (PDB, EcoCyc, Y2H, AP-MS), complemented by BioGRID benchmarks with explicit negatives under both balanced and imbalanced settings, and on reporting CM2.0's own runtime (Table S7), which reflects the design choices that make proteome-wide analysis computationally tractable. This rationale is explicitly stated in the revised manuscript." (Discussion, lines 417-425).

To clarify this point: structure-based predictors are explicitly designed to model residue-level physical binding and require computationally intensive structural modeling, while supervised ML predictors rely on labeled training data and are optimized for classification of physical PPIs under different assumptions. In contrast, CM2.0 is an unsupervised, sequence-only method designed to recover proteome-wide evolutionary and functional associations.

We believe the revised manuscript substantially strengthens the evaluation of CM2.0 within its intended design and addresses the reviewer's concerns in a considered and transparent way.

Reviewer #2:

All of my concerns have been addressed.

We thank the reviewer for their positive assessment and are pleased that all concerns have been addressed.

Reviewer #3:

Almost all of my comments were adequately addressed. The only comment that is still confusing to me is the one about the calculation of the random precision:

In your answer, you stated that “the denominator includes only the protein pairs that CM2.0 is able to evaluate (i.e., those with ≥1000 shared orthologs)”. However, in Supplementary Table S4, you calculate the random precision as true positives / potential PPIs, where the number of potential PPIs is calculated as [N*(N-1)]/2, N := number of proteins in the proteome of the species. I still think you need to adjust this to the number of protein pairs that ContextMirror 2.0 could potentially predict, which is the number of protein pairs that survive the filtering step. An alternative approach would be to draw a second line into the plot/make another panel in Figure 1, representing this form of random precision, so that readers can see how ContextMirror2.0 performs in comparison to the baseline, which simply predicts a PPI for all protein pairs that share at least 1000 orthologs.

We thank the reviewer for their assessment and agree that our previous explanation was unclear. The confusion arises from the use of the term *potential PPIs* in different contexts, which we clarify below.

In Table S3, we report the total number of theoretical protein pairs in the input proteome, computed as N*(N-1)/2, where N is the number of all proteins in the provided proteome. For *E. coli*, the input proteome contains 4,298 proteins, resulting in 8,944,334 possible protein pairs prior to any ortholog-based filtering.

In Table S4 N denotes the number of proteins that participate in at least one protein pair passing the ≥1000 shared ortholog filter. In the case of *E. coli,* 718,073 protein pairs satisfy this filter and these pairs involve 4,126 unique proteins. The random precision baseline in Table S4 is therefore computed over all pairwise combinations among these 4,126 proteins (4126×4125/2=8,509,875).

We have revised the table captions and text to clarify this distinction and avoid ambiguity between the total theoretical proteome-wide interaction space (Table S3) and the effective protein universe considered for benchmarking (Table S4).

Apart from that, I just have some small formal comments:
1. In the introduction, a sentence starting with “We benchmarked” is left unfinished.

We thank the reviewer for noticing this. The sentence beginning with “We benchmarked” was inadvertently left unfinished during revision. This has now been corrected in the Introduction (line 88).

2. There are some formatting artifacts from the markup, but maybe that was just the submission system. Examples are: “…are kept.-In these cases…”, “…where N M is the number of proteins…”, “…given a third protein (N k)…”, “…of proteins A p and B q…”, or the definition of the penalty term.

We agree and thank the reviewer for pointing this out. These formatting artifacts were introduced during file conversion and markup. We have carefully revised the manuscript to

correct these issues and ensure that all equations and text display correctly in the revised version (lines 469, 494, 520, 522, 564).

3. In the formula of r_{pq}, there is a ^2 missing in the first term of the denominator.

We thank the reviewer for catching this error. The square exponent (^2) was indeed missing in the first term of the denominator of the r_{pq} formula. This has now been corrected in the Methods section (line 474).

Reviewer #4:

My recommendations were not included in the decision letter.

We note that this reviewer's recommendations were not included in the original decision letter, so we were unable to address them. We are happy to respond to any specific concerns should they be communicated by the editor.

Reviewer #5:

The authors have adequately addressed all of the revision requests. They have made significant improvements to the manuscript, and the revised version has been substantially strengthened. The current version meets the standards for publication and is suitable for acceptance.

We thank the reviewer for their positive assessment and for recognizing the improvements made to the manuscript.
