# Supplementary material for: Sequence-based coevolutionary prediction of species-specific interactomes
Source: PLoS Comput Biol. 2026 Nov 6;22(11):e1014839. doi: 10.1371/journal.pcbi.1014839 (PMC13742926; doi:10.1371/journal.pcbi.1014839)
Supplement: Supplementary file 3 [file pcbi.1014839.s033.pdf]

Reviewer #6

The manuscript "Sequence-based coevolutionary prediction of species-specific interactomes" reports on a phylogeny-based method for detecting pairs of proteins with strong coevolution signal suggesting functional and/or physical interaction. The method is applicable at large scale. The authors report predictive performance and enrichment compared to random baseline and showcase how it can be used to get new biological insights about functional communities.

The title is somewhat an over-statement since the method was applied and validated only in bacteria and the authors recognised in the first revision that it would require adaptation to apply it to eukaryotic species, in particular for disentangling paralogs. These aspects should be made explicit in the manuscript.

We thank the reviewer for this observation. We agree that the current implementation and validation of CM2.0 were performed exclusively on bacterial proteomes, and that additional methodological considerations would be required for optimal application to eukaryotic species. In particular, the extensive presence of paralogous genes in eukaryotic genomes complicates ortholog assignment and may confound coevolutionary signals if not explicitly addressed.

While we have retained the title because the underlying framework is, in principle, applicable to any group of organisms for which reliable orthologous relationships can be established, we have revised the manuscript to clarify the current scope of the method. We now explicitly state that CM2.0 was designed, optimized, and benchmarked for bacterial proteomes, and that extension to eukaryotic systems would require additional developments, including improved handling of paralogy and other eukaryote-specific genomic features. We have added these clarifications to the Discussion section of the manuscript (**lines 624-628**):

Another limitation of the current framework is that CM2.0 was developed and validated using bacterial proteomes. Although the overall framework can, in principle, generate a putative list of protein interactions for any proteome as long as reliable orthologous relationships can be established, eukaryotic genomes present additional challenges, particularly the widespread occurrence of paralogous genes, which can dilute coevolutionary signals.

"For instance, although a S. aureus PPI received a high CM2.0 interaction score and was validated in experimental databases, the AF-predicted complex had a very low pDockQ2, suggesting that the proteins are functionally related but do not form a stable 375 physical interaction. " I would recommend to nuance the statement here. AlphaFold is unable to predict even certain complexes that are experimentally resolved with high resolution and extended interface.

We thank the reviewer for the clarification. We agree that a low pDockQ2 score should not be interpreted as evidence that two proteins do not physically interact, as AlphaFold multimer has in fact been reported to fail in a subset of experimentally solved protein-protein complexes. The intention of this section is to highlight the discrepancy between CM2.0 interaction score, that aims to capture broad coevolutionary relations, and the traditional idea

of protein complexes as physically interacting units. Accordingly, in the revised manuscript, the AlphaFold Multimer evaluation has been moved to the Supplementary Information and is presented only as an exploratory assessment of a subset of CM2.0 predictions. The text now states that high-IntScore interactions are more likely to be compatible with structural modelling, while emphasizing that many biologically relevant associations identified by CM2.0 are not necessarily mediated by stable physical complexes and therefore remain challenging for current structure-based predictors (**lines 248-255**).

To further characterize a subset of CM2.0 predictions, we modelled 60 randomly selected PPIs (10 high- and 10 low-IntScore pairs from each organism) using AlphaFold Multimer [27,28] and evaluated the resulting complexes using the pDockQ2 score [29] (see Methods). Three high-IntScore PPIs yielded acceptable structural models (pDockQ2 > 0.23), whereas only one of the low-scoring pairs did (Figure S4). These results suggest that high IntScore interactions are more likely to be compatible with structural modelling, although many biologically relevant associations identified by CM2.0 are likely not mediated by stable physical complexes and therefore remain challenging for current structure-based predictors.

To provide a more appropriate evaluation of CM2.0 against current methods, we have incorporated a new benchmarking section comparing CM2.0 with RoseTTAFold-PPI and D-Script on curated bacterial protein complexes (**lines 256-293**). These analyses now constitute the primary comparison with state-of-the-art approaches in the revised manuscript.

Quantitative comparisons are reported only against a random baseline. While I understand that there are few currently available methods that address exactly the same question as CM2.0 and that it is not so easy to reproduce or update with more recent sequence data the work of Cong et al. 2019, the reader is left unsure about the positioning of the present work with respect to SOTA in PPI prediction (or even prioritization). The lack of predictive performance or even runtime comparisons with RoseTTAFold-PPI or D-Script for instance is not satisfactorily justified. If CM2.0 is faster, then the authors should quantify how much. If CM2.0 has lower prediction or resolution, then the authors should (even roughly) estimate by how much, and discuss the tradeoff between predictive performance on one side, and speed, interpretability and lack of supervision on the other side. The argument of defining an alternative or niche task for which the only available tool is CM2.0 is a bit light.

We thank the reviewer for the suggestion. We agree that comparisons against modern PPI prediction methods provide useful context for positioning CM2.0 relative to the current state of the art. To address this concern, we performed additional benchmarking against RoseTTAFold-PPI and D-Script using curated protein complexes from both *E. coli* and *S. enterica*. For each species, we selected two experimentally characterized complexes located either in the mitochondrial inner membrane or the periplasm (Nuo and ATP synthase for *E. coli* and Dpp and Liv transporters for *S. enterica*) and generated all possible intra-complex protein pairs. As controls, we generated pairs between complex members and proteins belonging to unrelated cytosolic systems. These datasets were then evaluated with RoseTTAFold-PPI, D-Script, and CM2.0.

Since RoseTTAFold-PPI and D-Script assign scores to every queried protein pair, whereas CM2.0 only evaluates pairs that pass its initial coevolutionary filters, we first restricted the

comparison to the subset of protein pairs scored by CM2.0. In this common evaluation space, IntScore consistently showed stronger discrimination between experimentally validated and unsupported interactions than the alternative methods (Table 1).

**Table 1. Evaluation space reduced to match CM2.0 predictions**						
	validated vs unsupported			intra-complex vs inter-complex		
AUC	CM2.0	Dscript	RosettaFold-PPI	CM2.0	Dscript	RosettaFold-PPI
nuo (n=41)	**0.941**	0.748	0.521	**0.936**	0.75	0.595
atp (n=45)	**0.715**	0.558	0.51	**0.743**	0.649	0.620
dpp (n=14)	**0.95**	0.7	0.8	**0.95**	0.7	0.8
liv (n=10)	**0.792**	0.75	0.667	**1**	0.81	0.81

Since this first comparison isolates only the ranking component of CM2.0, we next evaluated the complete workflow by assigning an IntScore of 0 to protein pairs discarded during the coevolutionary filtering stage. This end-to-end comparison showed that the complete pipeline remained competitive, although its performance depended on the amount of evolutionary information available for each benchmark system (Table 2).

**Table 2. IntScore = 0 for pairs that did not pass the coevolutionary filters**						
	validated vs unsupported			intra-complex vs inter-complex		
AUC	CM2.0	Dscript	RosettaFold-PPI	CM2.0	Dscript	RosettaFold-PPI
nuo (n=208)	0.669	0.709	**0.777**	0.69	0.744	**0.827**
atp (n=60)	**0.741**	0.556	0.580	**0.820**	0.674	0.652
dpp (n=60)	**0.996**	0.506	0.858	**0.996**	0.506	0.858
liv (n=60)	0.634	0.545	**0.829**	0.601	0.578	**0.880**

To explain these differences, we analysed the behaviour of the coevolutionary filtering stage. We found that experimentally supported interactions were consistently more likely to pass the coevolutionary filter than unsupported interactions, demonstrating that the filter preferentially retains experimentally supported interactions rather than acting indiscriminately. These analyses revealed that the reduction in end-to-end performance observed in some systems is a consequence of limited shared evolutionary information rather than reduced discriminative power of the interaction score.

As an additional validation, we repeated the benchmarking using complex membership (intra-complex versus control pairs) as an independent proxy for functional association. The same qualitative conclusions were obtained (Tables 1 and 2)

Taken together, these analyses show that the advantage of CM2.0 arises from two complementary components. First, IntScore provides information beyond that captured by RosettaFold-PPI or D-script when evaluating the same candidate interactions. Second, the unsupervised coevolutionary filtering step substantially reduces the candidate interaction space while preferentially removing unsupported interactions. CM2.0 should therefore be understood as a prioritizing framework, rather than just a PPI scoring method.

This benchmarking analysis is explained in detail in the Results section (**lines 256-293**), the complex retrieval and prediction are explained in Methods (**lines 867-890**), and the corresponding supplementary analyses are presented in Figures S5-S7 and Tables S4-S6.

Although we did not perform a formal runtime benchmark because the methods address different computational problems, we note that RoseTTAFold-PPI required approximately 2-9 minutes per evaluated protein pair in our benchmarking experiments. In contrast, CM2.0 performs a single proteome-wide analysis, requiring approximately 45 s per scored interaction on average for *E. coli* (29 s in *S. enterica*), although much of this computational cost corresponds to shared preprocessing rather than pair-specific computation.

Reviewer #7

I think that the presented method is worth being published; however, the current state of the paper doesn't do it justice. The paper could greatly benefit from some editions to make it more self-contained and easier to read. Some clarifications are needed, and some extra tests could be valuable to ensure a proper evaluation of the presented method.

A. Method evaluation.

1. The CM2.0 method is presented as an improvement over a previous version, ContextMirror. However, the paper does not compare them directly, so it is difficult to address whether there is an improvement in the quality of the protein-protein interaction predictions. I guess based on some phrases, such as the one indicating that this is “a new high-throughput version of the ContextMirror method” that running the original method at the genome level is unfeasible. However, I imagine that some comparisons can still be done at the community level. This idea of picking a community can also be useful for comparing against more expensive methods, such as the ones predicting structures of the complexes (e.g., AF3) and the method from Cong et al. (2019). In such cases, picking a community defined from the ground truth PPI graph could be a better approach than picking one resolved by CM2.0.

We thank the reviewer for this comment. We agree that a direct comparison between the original ContextMirror implementation and CM2.0 would be valuable. Unfortunately, the original ContextMirror software is no longer available and the computational details required to reproduce the original analyses were not reported, making a rigorous comparison impractical. Moreover, the primary objective of CM2.0 was not simply to improve the predictive performance of ContextMirror, but to redesign the original framework as a scalable and automated pipeline capable of proteome-wide analyses. To achieve this, several components of the workflow were redesigned and adapted to high-performance computing

environments, enabling the systematic evaluation of hundreds of thousands of protein pairs and the reconstruction of species-level interactomes.

We also considered the reviewer's suggestion of comparing CM2.0 against alternative computationally intensive approaches. Although the method of Cong et al. (2019) would be particularly relevant in this context, its implementation is not publicly available and reproducing the framework from the published description would require a substantial independent development effort that we believe falls beyond the scope of the present study. Nevertheless, following the reviewer's suggestion, we performed additional benchmarking using curated protein complexes from EcoCyc, and compared CM2.0 against two publicly available state-of-the-art approaches, RoseTTAFold-PPI and D-Script. Specifically, we selected four experimentally characterized complexes from *E. coli* and *S. enterica*. These analyses evaluate both the discriminative performance of the CM2.0 IntScore and the contribution of the initial coevolutionary filter. IntScore consistently outperformed the alternative methods when evaluated on the same candidate interaction space, while the complete CM2.0 workflow remained competitive in end-to-end comparisons. We believe these new analyses provide a substantially stronger positioning of CM2.0 with respect to current approaches and address the reviewer's request for a more comprehensive evaluation on ground-truth derived protein communities.

This benchmarking analysis is explained in detail in the Results section (**lines 256-293**), the complex retrieval and prediction are explained in Methods (**lines 867-890**), and the corresponding supplementary analyses are presented in Figures S5-S7 and Tables S4-S6.

2. Figure 1 shows the “precision values that would be expected by chance”, which are calculated as the number of true positives over the potential PPIs after ortholog filtering, as stated in Table S4 and in the methods section. However, a reader of the paper needs to go to those places to know that. It would have been better to state that earlier in the manuscript. In any case, such a value is “too random” to be the only metric to compare CM2.0 against. I would propose that a better comparison could have been to measure the precision and recall of inferring functional interactions using operons as communities. An idea that it is qualitatively referenced in the paper, but there is no quantitative comparison between the approaches. Furthermore, most of the genes in the communities shown in Figure 5 belong to operons. I can see that CM2.0 can also capture relations among operons, but still, it would be the most sensible baseline. Another simple metric that could also be evaluated as a baseline is the number of shared putative-interacting ortholog sequences in the MSA. It would be nice to see the precision and recall curves of those baselines and to compare the AUC values.

We thank the reviewer for this suggestion. We agree that the random expectation shown in Figure 1 should not be interpreted as the only relevant baseline. In the revised manuscript, we now clarify earlier in the text that the random precision corresponds to the fraction of validated interactions among all possible protein pairs remaining after ortholog filtering (**line 174**).

The precision of our highest-ranked interactions significantly surpasses baseline rates, calculated as the fraction of validated interactions among all possible protein pairs after ortholog filtering (4.9% for E. coli, 4% for S. enterica, and 3.9% for S. aureus)

Following the reviewer's recommendation, we substantially expanded the evaluation of CM2.0 by introducing new baseline analyses based on biologically motivated predictors of functional association, namely the number of matching species contributing to the paired MSA, genomic proximity, and operon organization. Rather than comparing only overall precision, we explicitly evaluated whether IntScore provides predictive information beyond each of these variables.

First, because CM2.0 explicitly exploits coevolutionary information, we evaluated whether IntScore simply reflects the number of matching species used to construct the paired MSA. Since CM2.0 is built on orthology-guided coevolutionary profiles, some relationship between ortholog count and IntScore is expected. Indeed, we observed a moderate positive correlation between both variables ($r \approx 0.47–0.49$), indicating that protein pairs with broader ortholog coverage tend to receive higher interaction scores. Protein pairs were stratified according to the number of matching species, and within every bin validated interactions consistently received higher IntScores than unsupported interactions. Moreover, the separation between validated and unsupported interactions became progressively larger as the number of matching species increased, reflecting the greater amount of coevolutionary information available in deeper paired alignments. Logistic regression further confirmed that IntScore remained a significant predictor of interaction status after controlling for the number of matching species. Notably, in *S. aureus*, where ortholog coverage is substantially lower than in *E. coli* and *S. enterica*, CM2.0 retained strong predictive performance despite the more limited evolutionary information.

Second, we performed the same analysis using genomic proximity. As expected, neighbouring genes showed a high enrichment of experimentally validated interactions. Protein pairs were grouped according to intergenic distance and compared within distance bins. Again, validated interactions consistently received higher IntScores than unsupported interactions across all bins, and logistic regression confirmed that IntScore remained a significant predictor after accounting for genomic distance. Notably, the enrichment became particularly pronounced for distant gene pairs, where genomic proximity alone provides little to no predictive power.

Finally, we evaluated operon organization as a baseline. As expected, proteins encoded within the same operon generally received high IntScores and were frequently experimentally validated. However, high-confidence predictions were not restricted to intra-operon pairs. At an IntScore ≥ 1.8, CM2.0 recovered 53 validated same-operon interactions together with 2,202 validated interactions involving proteins not annotated in the same operon. Consequently, the ranked interactome is overwhelmingly composed of experimentally supported inter-operon predictions, and the changes in precision observed across IntScore thresholds are primarily driven by these interactions. Thus, although CM2.0 clearly captures operon organization, its predictive power extends well beyond operon structure by prioritizing functional associations between distinct transcriptional units.

Together, these analyses demonstrate that CM2.0 captures biologically meaningful information beyond simple ortholog overlap, genomic proximity and operon organization. These new baseline analyses have been incorporated into the Results (**lines 219-247**), together with Figure S3 and Tables S2-S3.

3. In the section where AlphaFold-Multimer is used to assess selected predicted PPIs, the authors rely on pDockQ2 as the main confidence metric. I would suggest that the authors use ipTM or the combined AlphaFold-Multimer ranking confidence score (0.8·ipTM + 0.2·pTM) instead or as a complement. This would make the analysis more consistent with standard practice and would provide a more appropriate assessment of whether high-scoring CM2.0 pairs also tend to yield confident complex models. Furthermore, it would be great to compare ipTM (and the combined score) and the IntScore using a continuous metric, such as the Pearson correlation coefficient.

We thank the reviewer for this suggestion. We agree that ipTM and the AlphaFold-Multimer ranking confidence are widely used metrics for assessing the confidence of predicted protein complexes. However, following the reviewers' comments, we substantially revised the evaluation of CM2.0. Rather than expanding the structural benchmarking, we moved the AlphaFold-Multimer analysis to the Supplementary Material and now present it only as a qualitative characterization of a small subset of predicted interactions, where pDockQ2 is reported as an indicator of interface confidence rather than as a systematic validation of the method. The main validation has been replaced by a comprehensive benchmarking against the state-of-the-art PPI prediction methods RoseTTAFold-PPI and D-Script using experimentally characterized bacterial protein complexes. We believe this provides a more informative and biologically relevant assessment of CM2.0 than correlations with AlphaFold confidence metrics derived from a limited sample of predicted complexes.

4. Can CM2.0 be applied to non-bacterial genomes? Since the method was tested only on bacterial species, it is difficult to determine from the manuscript whether it generalizes beyond bacteria. If it is intended only for bacterial genomes, this limitation should be stated clearly. If not, including an eukaryotic example, ideally *H. sapiens* because of the abundance and quality of available PPI annotations, would strengthen the paper.

We thank the reviewer for this comment. CM2.0 was designed, optimized and validated using bacterial proteins, and we agree that this scope should be stated more explicitly in the manuscript. We have revised the text to clarify this in the Discussion (**lines 624-633**).

Another limitation of the current framework is that CM2.0 was developed and validated using bacterial proteomes. Although the overall framework can, in principle, generate a putative list of protein interactions for any proteome as long as reliable orthologous relationships can be established, eukaryotic genomes present additional challenges, particularly the widespread occurrence of paralogous genes, which can dilute coevolutionary signals. Moreover, CM2.0 assumes that orthologous proteins follow tree-like evolutionary histories, so processes such as horizontal gene transfer may introduce confounding coevolutionary signals. However, it is also possible that these events reflect biologically meaningful co-transferred functional modules, as previously observed in phylogeny-based coevolution analysis [63], distinguishing between these scenarios is outside the scope of the current framework.

In principle, the CM2.0 framework can be applied to any collection of proteins for which reliable orthologous relationships can be established. However, eukaryotic genomes introduce additional challenges that were not addressed in the present work, most notably the widespread occurrence of paralogous genes and increased complexity of ortholog

assignment. These factors can complicate the interpretation of coevolutionary signals and would require additional methodological developments before the approach could be considered optimized for eukaryotic proteomes. Bacterial systems provide a suitable setting for this conceptual validation because ortholog assignment is more straightforward, large numbers of related genomes are available, and extensive experimental interaction data exist for benchmarking. We now explicitly discuss these limitations and identify the extension of CM2.0 to eukaryotic systems as an important direction for future work.

B. Other comments and questions.

Most of the style problems and missing details I have found came from the fact that the methods section is at the end. The methods section is well written and detailed; however, a reader that tackles the paper linearly is unable to fully understand the results. I would recommend introducing some method details early to ease readers tackling the paper linearly.

We thank the reviewer for his helpful comment. To address this concern, we have revised the manuscript to introduce key methodological concepts at the beginning of the relevant Results sections (**lines 133, 156, 173, 314,338**). These include the clarification on the prior filtering of protein pairs or the homolog-based mapping used for multispecies comparisons. Some examples:

CM2.0 first applies a coevolutionary filtering stage to remove protein pairs lacking sufficient evolutionary support and subsequently assigns an interaction score (IntScore) to the retained candidates.

This cutoff was selected because of the relationship between phylogenetic correlation and the number of influencing proteins levels off below this value, and statistical analyses further demonstrated a significantly higher concentration of false positives in this range (see Methods and Figure S1).

The precision of our highest-ranked interactions significantly surpasses baseline rates, calculated as the fraction of validated interactions among all possible protein pairs after ortholog filtering

To achieve this, we identified common and distinct PPIs by mapping homologous sequences across species through reciprocal best-hit homolog relationships (see Methods).

We generated a multispecies PPI network by combining these two by matching the homologous proteins (nodes) that were identified in the previous step.

1. I have found the abstract to be a little cryptic, as “coevolutionary approaches” can mean many things; I will emphasize in the abstract that CM2.0 is measuring coevolution at the phylogenetic level, similarly to MirrorTree.

We thank the reviewer for the comment. We agree that the term “coevolutionary approaches” can mean many things. The method is rooted in phylogenetic similarity, like

MirrorTree, which is the spiritual and methodological ancestor of this framework. We have now made this clear in the abstract and clarified this concept early in the Introduction:

CM2.0 leverages coevolutionary information at the phylogenetic level to explore protein interaction dynamics and understand the functional consequences of species-specific variations.

To address these challenges, we have developed ContextMirror2.0 (CM2.0), a coevolution-based method built upon our previous work [22]. CM2.0 is a new implementation of the ContextMirror framework based on a redesigned algorithm that enables efficient proteome-scale prediction while preserving the original concept of exploiting phylogenetic context to capture coevolutionary signals.

2. In the introduction, in the third paragraph, I would also acknowledge the current PPI models in AFDB and works such as Todor et al., "Predicting the protein interaction landscape of a free-living bacterium with pooled-AlphaFold3" in Molecular Systems Biology (2026). The field advances so quickly that this paragraph needs an update.

We thank the reviewer for the comment. It is true that the field evolves faster than the revision process and therefore we have updated the third paragraph of the Introduction to acknowledge recent advances in structure-based proteome-wide PPI prediction by citing the suggested work.

Furthermore, the explainability of ML models, particularly deep neural networks, is often limited, and most of the existing PPI prediction methods are either supervised [15–17] or rely on structural information and do not work for large scale prediction [18–20]~~, which poses a challenge for systematic benchmarking~~. Although recent developments have improved the scalability of structure-based approaches [21], systematic proteome-wide prediction remains computationally demanding and challenging to benchmark. In the case of network-based approaches such as PPISB, additional limitations include poor scalability to very large datasets and reduced recall in highly complex networks.

3. In the fourth introduction paragraph, I would add a longer description of the previous method, ContextMirror (and make more clear what CM 2.0 introduces). As mentioned before, coevolution can be measured among different entities, and the reader needs to know it as early as possible when reading the paper which one is being used here.

We thank the reviewer for this suggestion. We agree that readers unfamiliar with the original ContextMirror publication may benefit from a brief description of the underlying coevolutionary framework earlier in the Introduction. We have therefore expanded the fourth introductory paragraph to summarize the conceptual basis of ContextMirror, namely the use of phylogenetic coevolutionary profiles derived from orthologous protein families and the comparison of these profiles to identify functionally associated proteins. We also clarify how CM2.0 was conceived and what we believe contributes to the field (**lines 84-102**).

To address these challenges, we have developed ContextMirror2.0 (CM2.0), a coevolution-based method built upon our previous work [22]. CM2.0 is a new implementation of the ContextMirror framework based on a redesigned algorithm that enables efficient proteome-scale prediction while preserving the original concept of exploiting phylogenetic context to capture coevolutionary signals.

We believe its core idea remains valuable, as it relies solely on evolutionary information and does not require training data or supervision, providing a complementary perspective to existing PPI prediction methods. The workflow first filters protein pairs according to the number of matching species represented in their paired alignments, excluding pairs lacking sufficient evolutionary information for reliable coevolutionary analysis, and then ranks the retained pairs according to the strength of their coevolutionary signal. For the retained pairs CM2.0 measures coevolution by comparing sequence distance patterns among orthologous protein families across species. In other words, it leverages the coevolutionary network of an organism, defined as the network containing all of the pairwise phylogenetic tree similarities, to quantify the coevolutionary signal between two proteins. The central idea is that proteins involved in the same biological processes tend to exhibit similar patterns of evolution with other proteins. CM2.0 compares these evolutionary profiles to measure their coevolutionary signal, which is then used to predict functional associations. Unlike existing approaches, CM2.0 is entirely unsupervised and sequence-based, making it fundamentally different from (ML)-based methods. Additionally, it provides a confidence score of the predictions, providing a reliable guide for future experimental and computational research.

4. Can you really find species-specific interactions? While comparing species can highlight species-specific interactions, the level of resolution may be limited by the fact that at least 1,000 common species are needed when searching for orthologs. Therefore, it is possible that the method is actually detecting interactions that are specific at the genus or family level. However, truly species-specific interactions could also arise in contexts without that degree of phylogenetic conservation, and the method may fail to detect them. This is something that needs to be clarified or tested, as the current title of the article seems to suggest that the method identifies species-specific interactions.

We thank the reviewer for raising this important point. We agree that the notion of “species-specific interactions” requires careful interpretation in the context of phylogeny-based methods such as CM2.0. Because our approach relies on the comparison of ortholog presence across a large number of species, the detectable signal necessarily reflects evolutionary patterns at a certain phylogenetic depth, and may therefore capture interactions that are specific to a lineage (e.g. genus or family) rather than strictly restricted to a single species.

In this work, we use the term “species-specific” in an operational sense: interactions that are present in one species but not observed in another species under comparison. For example, as shown in the manuscript, we identify interactions that are specific to *S. aureus* when compared to *E. coli* or *S. enterica*. These results demonstrate that CM2.0 can capture biologically meaningful differences between species.

We acknowledge, however, that this definition does not imply that CM2.0 can detect all interactions that are strictly unique to a given species, particularly those that do not exhibit sufficient phylogenetic conservation. We have clarified this point in the revised Results section (**lines 314-320** and **374-379**).

To achieve this, we identified common and distinct PPIs by mapping homologous sequences across species through reciprocal best-hit homolog relationships (see Methods). In this context, we define species-specific interactions as those detected in one species but absent from the corresponding network of another species included in the analysis. For instance, the examples below highlight interactions identified in S. aureus that are not detected in either E. coli or S. enterica. These comparisons demonstrate CM2.0's capacity to distinguish biologically relevant differences in interaction networks across species.

Here, species-specific is used in an operational sense to denote interactions predicted in one species but not in the other species under comparison; these unannotated interactions are currently not reported in the reference datasets but constitute high-confidence CM2.0 predictions, some of which may correspond to genuine lineage-specific or currently unannotated interactions. Complementary data sources, such as transcriptomic co-expression, could provide additional orthogonal support for these interactions.

5. In the first paragraph of the Results section, the authors note that strongly correlated protein pairs tend to be influenced by many third proteins. I wonder to what extent this trend may be expected from the topology of protein-protein interaction networks themselves, which are often described as having a small-world, modular, and hub-rich architecture. In such networks, proteins belonging to the same functional neighborhoods or dense communities would naturally be associated with many indirect contextual influences. Therefore, this pattern may reflect not only the specific biological signal captured by CM2.0 but also a property of PPI network organization. I do not necessarily see this as a flaw of the method, but I think it would be worth commenting on it explicitly in the manuscript.

We thank the reviewer for this observation and agree that the tendency of strongly correlated protein pairs to be associated with many third proteins may, at least in part, reflect fundamental properties of biological interaction networks. Protein interaction networks are typically modular and hub-rich, so proteins within the same functional neighborhood are connected through both direct and indirect relationships. As a result, proteins involved in coherent biological processes typically share contextual influences from other members of the same functional module. Thus, proteins embedded in dense communities accumulate larger numbers of significant third-protein associations.

This interpretation is consistent with the conceptual framework of CM2.0. Rather than focusing exclusively on direct pairwise coevolution, the method was designed to exploit high-order coevolutionary relationships and to identify proteins that participate in shared evolutionary contexts. Consequently, the reviewer's observation that high-ranked piers tend to be associated with many third proteins reflects the underlying organization of interaction networks and the ability of CM2.0 to capture these functional dependencies.

We have added a brief discussion of this point in the revised manuscript to clarify that the observed pattern should be interpreted as a biologically meaningful consequence of the modular organization of cellular networks (**lines 138-143**).

*There is also a positive relationship between phylogenetic correlation and the number of influencing third proteins (**Figure S1**), suggesting that more strongly correlated profiles tend to be influenced by many third proteins. This relation is consistent with the modular organization of protein interaction*

networks in which proteins involved in related biological functions are connected through multiple direct and indirect relationships.

6. You have tested different IntScore thresholds (1.6, 1.7 and 1.8) but you have a ground truth data to compare with. How can users without such data set a proper IntScore value?

We thank the reviewer for the question. The purpose of evaluating multiple IntScore thresholds (1.6, 1.7 and 1.8) was not to define universal cutoffs, but rather to illustrate the relationship between prediction confidence and coverage. As expected, increasing the IntScore threshold results in smaller sets of predictions enriched in validated interactions, whereas lower thresholds increase the number of candidate interactions at the expense of including less confident predictions.

Importantly, CM2.0 was designed to generate a ranked interactome rather than a binary classification of interacting versus non-interacting proteins. Therefore, in scenarios where no validation dataset exists, we do not recommend relying on a single fixed threshold. Instead, users can interpret IntScore as a confidence measure and prioritize predictions according to their specific objectives. For example, highly conservative studies may focus on the strongest interactions, whereas exploratory analyses may consider larger portions of the ranked network.

The thresholds included in the manuscript are intended to demonstrate this trade-off and to provide guidance on how IntScore values relate to prediction reliability. We have clarified this point in the revised manuscript (**lines 159-168**).

CM2.0 interaction score (IntScore) is a number between 0 and 2. The selection of an appropriate IntScore threshold is crucial when analysing CM2.0 predictions, as it serves as a confidence indicator ~~quality filter~~. The ideal threshold will depend on the application. A low threshold will include a high number of PPIs of both high- and low-confidence, while a high threshold will include a lower number of high-confidence PPIs. Here, we considered three IntScore thresholds: 1.6, 1.7, and 1.8, adjusting them as needed to address specific research questions. These thresholds should be interpreted as representative confidence levels that illustrate the trade-off between coverage and reliability. In the absence of benchmark interaction data, users may select more stringent or permissive portions of the ranked interactome depending on whether their goal is high-confidence prediction or broader exploratory analysis.

7. When creating structural models, the authors tested whether these PPIs are present in the PDB and found that they are not. However, some of them may still have homologous interactions represented in the PDB, including entries deposited before the AlphaFold-Multimer training-data cutoff. If you want, you can use Foldseek-Multimer to find similar interfaces on the PDB.

We thank the reviewer for this suggestion. We agree that homologous interactions may be represented in the PDB even when the exact protein pair is absent. A systematic search for homologous interfaces using tools such as Foldseek-Multimer could provide additional structural context for these predictions. However, the goal of the present analysis was not to establish structural homology or identify template complexes, but rather to assess whether

structure-based predictors (e.g. AF-Multimer) confidence scores showed a relationship with CM2.0 interaction scores. We have now expanded the benchmarking section by comparing CM2.0 against RoseTTAFold-PPI and D-Script on experimentally characterized protein complexes and negative control pairs from *E. coli* and *S. enterica*. These analyses provide a broader and more quantitative assessment of the relationship between CM2.0 predictions and alternative PPI prediction frameworks than the exploratory AF-Multimer examples.

8. A Venn diagram is the most effective way to represent Figure 3. If it doesn't work, default to a bar chart, as it is more appropriate than a pie chart.

We thank the reviewer for the suggestion. Because the comparison is based on pairwise homolog relationships rather than simple set intersections, a conventional Venn diagram is not appropriate. We therefore replaced the pie chart with a bar-chart representation. Figure 3 has been updated.

9. The last paragraph of page 8 does not provide a clear assessment of what fraction of these 'potentially novel PPIs' are likely to correspond to true functional interactions. If I understand correctly, these are interactions predicted by CM2.0 that are absent from the PPI databases used as ground truth. Therefore, based on the current analysis alone, they are more accurately described as false positives rather than as novel PPIs. Of course, some may indeed be genuine unannotated interactions, but the manuscript does not establish the likelihood of that scenario. Could transcriptomic data can give another layer of evidence to assess those cases? Perhaps those novel PPIs are due to functional rather than to physical association; therefore, better captured by transcriptomic associations than by conventional PPI annotations in databases.

We thank the reviewer for this comment. We would like to clarify that we did not intend to imply that interactions predicted with high confidence, but absent from the databases used as ground truth should be considered true positives. Rather, we used the term "potentially novel PPIs" to distinguish these predictions from interactions already reported in the reference databases and to highlight them as candidates for further investigation.

More specifically, the manuscript includes three categories of unannotated predictions. The first comprises 104 interactions predicted in both species that are absent from the *S. enterica* ground-truth dataset but reported in the *E. coli* ground-truth dataset, and 78 interactions predicted in both species that show the reciprocal annotation pattern. We consider these cases plausible candidates for missing or incomplete annotation in one species because they have independent support from the corresponding orthologous interaction in the other species.

A different category comprises 610 high-confidence interactions predicted in both species that are not currently annotated in either ground-truth dataset. We agree that the present analyses cannot establish what fraction of these interactions correspond to genuine biological associations. Nevertheless, their independent prediction in both species makes them plausible candidates for previously uncharacterized conserved associations, which could be further assessed using complementary sources of evidence such as transcriptomic co-expression or other functional association data, as pointed out by the reviewer.

The third category comprises 163 and 165 high-confidence interactions predicted only in *E. coli* or *S. enterica*, respectively, that are not annotated in the corresponding ground-truth datasets. In the revised manuscript, we clarify that these interactions are described as species-specific in an operational sense, meaning that they are predicted in one species but not in the other species included in the comparison, rather than implying definitive species exclusivity.

Consequently, we have revised the manuscript to clarify this distinction and state that complementary data sources (e.g. transcriptomic co-expression) could provide additional orthogonal support for these interactions (**lines 360-380**).

This analysis identified three sets of interesting PPIs: (1) PPIs reported in one species but not in the other (sets 1-4 and 2-3 in ***Figure 4****), (2) unreported PPIs identified in both species (set 1-3 in* ***Figure 4****), and (3) both reported and unreported species-specific PPIs (sets 4, 2, 1 and 3 in* ***Figure 4****). Specifically, we identified around 104 potentially novel PPIs in S. enterica (reported in E. coli), and 78 potentially novel PPIs in E. coli (confirmed in S. enterica)~~;~~. These cases may represent missing or incomplete annotations in one of the two species. ~~610 previously uncharacterized PPIs shared by both species, and 249 and 226 potentially novel species-specific PPIs in E. coli and S. enterica, respectively. These cases may represent missing or incomplete annotations in one of the two species.~~ In addition, we identified 610 high-confidence PPIs shared by both species that are not currently annotated in either ground-truth dataset. Although these interactions cannot be considered validated on the basis of the present analysis alone, their independent prediction in both species makes them plausible candidates for previously uncharacterized conserved associations, which could be further assessed using complementary sources of evidence such as transcriptomic co-expression or other functional association data. Finally, we identified 249 and 226 high-confidence species-specific PPIs in E. coli and S. enterica, respectively, of which 163 and 165 were not annotated in the corresponding ground-truth datasets. Here, species-specific is used in an operational sense to denote interactions predicted in one species but not in the other species under comparison; these unannotated interactions are currently not reported in the reference datasets but constitute high-confidence CM2.0 predictions, some of which may correspond to genuine lineage-specific or currently unannotated interactions. Complementary data sources, such as transcriptomic co-expression, could provide additional orthogonal support for these interactions. Similar results, though with lower precision, were observed at lower interaction score thresholds (****Table S7, Figures S10 and S11****).*

10. When reading the paper, I often wondered to what extent your results could be confounded by operon organization and synteny, especially in Section 2.3, since most of the interactions shown in Figure 5 involve proteins encoded in the same operon. To account for synteny, it could be useful to include intergenic distance as an additional baseline, since it may serve as a proxy for both factors. However, I would appreciate it if you could find another way to better control or test for synteny.

We agree that operon organization and local genomic context are potential confounding factors when interpreting coevolution-based predictions. Following the reviewer's suggestion,

we performed additional analyses explicitly evaluating operon organization, genomic proximity and the number of matching species as baseline predictors of protein interactions.

As expected, proteins encoded within the same operon generally received high IntScores and were frequently experimentally validated. However, high-confidence predictions were not restricted to intra-operon pairs. At an IntScore ≥ 1.8, CM2.0 recovered 53 validated same-operon interactions together with 2,202 validated interactions involving proteins not annotated in the same operon. In reality, the ranked interactome is basically composed of inter-operon predictions, and the changes in precision observed across IntScore thresholds are primarily driven by these interactions. These results indicate that although CM2.0 captures operon organization, it also identifies functional associations between distinct transcriptional units.

To address synteny more directly, we evaluated intergenic distance as an additional baseline. Expectedly, neighbouring genes were strongly enriched in experimentally validated interactions. However, after stratifying protein pairs by intergenic distance, validated interactions consistently received higher IntScores than unsupported interactions within every distance class. Moreover, the enrichment provided by CM2.0 became particularly pronounced for distant gene pairs, where genomic proximity alone provides little to no predictive power. Logistic regression further confirmed that IntScore remained a highly significant predictor of validated interactions after controlling for genomic distance.

Together, these analyses demonstrate that operon organization and genomic proximity contribute to the coevolutionary signal captured by CM2.0 but do not fully explain its predictive performance. These new baseline analyses have been incorporated into the Results (**lines 219-244**), together with Figure S3 and Tables S2-S3.

11. Because this method is applied to bacterial genomes, where horizontal gene transfer can be common, I think the manuscript should explicitly acknowledge that non-tree-like evolutionary histories lie outside the scope of the presented method, if I understood correctly.

We agree with the reviewer that horizontal gene transfer and other non-tree-like evolutionary histories are an important limitation for phylogeny-based approaches, particularly in bacteria. CM2.0 assumes that the phylogenetic signal captured by orthologous protein trees is informative for coevolutionary association, but proteins affected by HGT may display evolutionary histories that deviate from the underlying species tree. Such cases may either introduce spurious correlations or reflect co-transferred functional modules, and distinguishing these alternatives is outside the current scope of the method. We have now explicitly acknowledged this limitation in the Discussion (**lines 628-633**).

Moreover, CM2.0 assumes that orthologous proteins follow tree-like evolutionary histories, so processes such as horizontal gene transfer may introduce confounding coevolutionary signals. However, it is also possible that these events reflect biologically meaningful co-transferred functional modules, as previously observed in phylogeny-based coevolution analysis [68], distinguishing between these scenarios is outside the scope of the current framework.

12. Does ABD30300.1 occupy a network environment similar to that of fakA? More generally, to what extent can similarity in network topology be used to transfer functional annotations across species?

We thank the reviewer for this interesting question. Our proposed annotation of ABD30300.1 was not based on a direct comparison of its network topology with that of FakA. Rather, it resulted from the comparative analysis of homologous communities across species. After superimposing the homologous communities, we observed that ABD30300.1 interacts with FabF. Since FakA has been reported to interact with FabF, we then performed a homology analysis, as described for WP_000471571.1, and found that homologs of ABD30300.1 are annotated as FakA in other *S. aureus* strains and/or bacterial species. This combination of evidence motivated our proposed functional assignment. The same rationale was applied to the remaining examples (ABD30884.1, ABD30563.1, ABD29966.1, etc.), where interaction context was complemented by homology searches to propose functional annotations.

Regarding the more general question, we agree that exploiting network topology for functional annotation transfer is an interesting idea. In particular, topological similarity could complement the homology-based superimposition strategy that we adopted. This could potentially improve the identification of candidate proteins for functional annotation. We believe this represents an interesting direction for future work.

13. In many places, correlation coefficients are filtered using p-value cutoffs. However, it is not always clear at which steps FDR or Bonferroni corrections are applied. As far as I understand, a Bonferroni correction is explicitly used for the profile-profile correlation step but not clearly for the other filtering steps. If multiple-testing correction is not applied throughout, would the results improve, or at least become more statistically consistent, by considering FDR or another multiple-testing correction at each filtering step?

We thank the reviewer for this observation. Multiple-testing correction is applied only at the profile-profile correlation stage (Step 2), where all coevolutionary profiles are systematically compared at the proteome scale and the multiple-testing burden is substantial. In contrast, the initial tree-tree correlation filter ($p \leq 10^{-5}$) and the final partial-correlation filter ($p \leq 10^{-6}$) are inherited from the original ContextMirror implementation and are used as sequential screening and refinement steps rather than as independent proteome-wide hypothesis-testing procedures.

The rationale for this distinction is described in the Methods (**lines 6713-724**). Briefly, the much longer coevolutionary profiles used in CM2.0 make the original significance threshold ($p \leq 10^{-5}$) effectively uninformative at the profile-profile correlation stage, motivating the introduction of a Bonferroni-adjusted threshold ($p \leq 10^{-11}$). We agree that alternative approaches such as FDR correction could also be considered but we chose a Bonferroni correction because our objective was to conservatively control false positives while minimally modifying the original ContextMirror statistical framework.

14. It was not super clear to me how partial correlations are used to determine the number of third proteins influencing a given correlation. I would have expected this to be done, for example, by comparing the observed correlation with the corresponding partial correlations and then identifying how many third proteins produce a meaningful deviation. However, the

paper only states that this is done 'by setting a correlation cut-off,' without clearly explaining how that cutoff is defined or applied.

We thank the reviewer for the suggestion. We agree that our previous wording could be confusing in relation to how third protein influences are addressed. For each protein pair, we quantify the influence of every third protein by calculating the corresponding partial correlation coefficient. Third proteins are considered influential if their partial correlation is statistically significant ($p \leq 10^{-6}$), following the original ContextMirror implementation. Therefore, the number of influencing third proteins simply corresponds to the number of significant partial correlations and does not require any additional correlation threshold. The threshold of 50 influencing proteins is introduced only later during the calculation of the interaction score, where it is used as part of the penalization strategy to reduce the impact of highly specific correlations supported by only a few third proteins. We have revised the Methods section to make this distinction explicit (**lines 741-750**).

As a result, for each protein pair, a list of partial correlations with third proteins whose partial correlations remain statistically significant ($p \leq 10^{-6}$) is produced. This list displays the number of third proteins that influence the correlation between the coevolutionary profiles of a pair of proteins, and the magnitude of this influence, represented by the partial correlation coefficient. ~~This way, a protein pair that is influenced by a few or none third proteins or with low partial correlations could be considered as a highly specific correlation. By setting a correlation cut-off, we can extract the number of third proteins influencing the interaction of a protein pair and identify groups of proteins influenced by the same third profiles, indicative of functional relatedness.~~ This way, protein pairs influenced by only a few third proteins represent more specific coevolutionary relationships, whereas pairs influenced by many third proteins are more likely to belong to broader functional modules.

15. Selecting random seed 76 because it optimizes precision for shared predictions raises a concern about selection bias in the illustrative examples. Although the main quantitative results are averaged across 100 runs, the manuscript should clarify whether the communities shown in Figures 5, 6, and S5-S11 are representative of typical runs or correspond to a best-case scenario.

We thank the reviewer for this comment. The communities shown in Figures 5, 6 and S5-S11 (now S9-S15) are neither intended to represent an average run nor were they selected by manually searching for particularly compelling biological examples. Community detection is stochastic, so a single realization had to be chosen for visualization and the procedure described in the Methods was intended precisely to avoid subjective selection of examples. Rather than selecting one arbitrarily, we performed 100 independent runs and selected the realization that maximized a predefined objective criterion: the precision of the shared-prediction network across species (seed = 76). The illustrative communities were then extracted from this single realization.

Importantly, all quantitative analyses reported in the manuscript, including community precision and recall values, were calculated by averaging the results obtained across the full set of 100 runs and therefore do not depend on the selected seed. The choice of seed only affects the specific community layout shown in the figures and not the conclusions of the study.

16. Are the modifications mentioned in the last paragraph of Section 4.3 the only ones from ContextMirror to CM2.0? This should be briefly mentioned or described previously in the manuscript.

We thank the reviewer for this suggestion. We agree that the methodological advances of CM2.0 relative to the original ContextMirror framework should be introduced earlier in the manuscript. We have therefore revised the Introduction to briefly summarize the methodological developments (**lines 99-107**). The final paragraph of Section 4.3 continues to describe the implementation-level improvements to the computational pipeline.

Unlike existing approaches, CM2.0 is entirely unsupervised and sequence-based, making it fundamentally different from (ML)-based methods. Additionally, it provides a confidence score of the predictions, providing a reliable guide for future experimental and computational research. Compared with the original ContextMirror framework, CM2.0 introduces several methodological advances, including a redesigned algorithm that enables efficient proteome-scale analyses, an automated workflow for ortholog retrieval and paired MSA construction, revised statistical procedures for interaction scoring, and an unsupervised community-detection framework for network analysis, together with numerous technical improvements to modernize the computational implementation.

C. Text.

1. The abstract phrase starting with “While around 40%” is too long and difficult to understand.

We thank the reviewer for the observation and have consequently adapted this sentence to be easier to read (**lines 25-28**).

Around 40% of CM2.0's top-1000 predicted PPIs for E. coli, S. enterica, and S. aureus are supported by experimental PPI databases. However, comparative analysis of the resulting functional communities further revealed both highly conserved interaction patterns and species-specific interactions.

2. The manuscript reports p-values as 0.0. Since p-values are not exactly zero, these should be reported as inequalities or in scientific notation.

We thank the reviewer for pointing this out. Statistical analysis was performed using scipy v1.8 in a 64-bit system. Therefore in this case a p-value = 0.0 reflects a fundamental limitation of floating point arithmetic. If the p-value is smaller than the smallest (normal) double precision floating point number (~1e-308), it underflows and the number is rounded to zero. Because the analyses involve very large numbers of observations and highly significant test statistics, some p-values become smaller than the minimum value. We have revised the manuscript to report these results as $p < 0.00001$, avoiding the incorrect notation of $p = 0.0$ (**line 194**).

3. In page 7, there is a mention of an “interaction score (IS)”; is that the same as the IntScore described in Methods? Note that “IS” is also used in other parts of the manuscript.

We thank the reviewer for finding this inconsistency. The interaction score was renamed from IS to IntScore during manuscript revision, but two occurrences on **lines 336 and 340** were inadvertently overlooked. These have now been corrected. The remaining occurrences of IS were found in the supplementary material captions, where the original terminology had also not been updated. These have now been corrected throughout the supplementary material, and the revised version consistently uses IntScore. We also removed the incorrect legend from Figure 4, which still contained the outdated IS nomenclature.

4. The paragraph beginning with 'Using the same curated datasets' in the Discussion section presents results that would be more appropriately placed in the Results section.

We thank the reviewer for the suggestion. The paragraph has been relocated to the Results section (**lines 294-303**), where these benchmarking results are presented alongside the other validation analyses. The Discussion has been revised accordingly and now focuses on the interpretation of these findings, rather than presenting quantitative results:

The benchmarking performed in this work, together with our analysis of the curated datasets of Cong et al. [6] and BioGRID, consistently show that CM2.0 enriches experimentally supported interactions above random expectation across diverse benchmarking scenarios.

5. The last paragraph of Section 4.2 would be better placed at the figure description in the Results section.

We thank the reviewer for this suggestion. We have expanded the captions of Figures 5 and 6 to include the visual conventions used in the network representations, including the distinction between validated (solid) and unvalidated (dashed) interactions. The detailed description of the network construction remains in the Methods section to avoid unnecessary duplication.

Figure 5. Examples of protein communities detected in the combined PPI network of E. coli and S. enterica at the threshold of *IntScore* ***> 1.7 containing species-specific PPIs****.* ***A)*** *A community containing proteins from the phn gene cluster.* ***B)*** *A community containing three electron transfer flavoproteins (STM0855, STM0856 and STM0858).* ***C)*** *A community containing proteins encoded by the yej gene cluster.* ***D)*** *A community containing proteins encoded by genes form the xyl operon. The gray nodes/edges are common to both species, while the blue nodes/edges are only present in E. coli and the orange nodes/edges are only present in S. enterica. Solid edges indicate experimentally validated interactions and dashed edges indicate interactions without experimental validation.*

Figure 6*.* ***Predicted protein-protein interactions (PPIs) for the mur, pur, and acc+fab complexes in E. coli, S. enterica, and S. aureus.*** *Interactions were filtered using the following interaction thresholds:* ***A)*** *1.2 for the mur complex,* ***B)*** *1.2 for the pur complex, and* ***C)*** *1.7 for the acc+fab complex. Shared nodes and edges are shown in gray, E. coli predictions in blue, S. enterica predictions in orange, and S. aureus predictions in red. Solid edges indicate experimentally validated interactions and dashed edges indicate interactions without experimental validation.*
