# Supplementary material for: Sequence-based coevolutionary prediction of species-specific interactomes
Source: PLoS Comput Biol. 2026 Nov 6;22(11):e1014839. doi: 10.1371/journal.pcbi.1014839 (PMC13742926; doi:10.1371/journal.pcbi.1014839)
Supplement: Peer review history

# Peer review: Decision Letter 0

**DOI:** 10.1371/journal.pcbi.1014839.r001

**Authors:** Shihua Zhang, Hugues Richard

6 Jun 2025

PCOMPBIOL-D-25-00705

Sequence-based coevolutionary prediction of species-specific interactomes

PLOS Computational Biology

Dear Dr. Fernandez-Martin,

Thank you for submitting your manuscript to PLOS Computational Biology. After careful consideration, we feel that it has merit but does not fully meet PLOS Computational Biology's publication criteria as it currently stands. Therefore, we invite you to submit a revised version of the manuscript that addresses the points raised during the review process.

Please submit your revised manuscript within 60 days Aug 06 2025 11:59PM. If you will need more time than this to complete your revisions, please reply to this message or contact the journal office at ploscompbiol@plos.org. When you're ready to submit your revision, log on to https://www.editorialmanager.com/pcompbiol/ and select the 'Submissions Needing Revision' folder to locate your manuscript file.

Please include the following items when submitting your revised manuscript:

* A rebuttal letter that responds to each point raised by the editor and reviewer(s). You should upload this letter as a separate file labeled 'Response to Reviewers'. This file does not need to include responses to formatting updates and technical items listed in the 'Journal Requirements' section below.

* A marked-up copy of your manuscript that highlights changes made to the original version. You should upload this as a separate file labeled 'Revised Manuscript with Track Changes'.

* An unmarked version of your revised paper without tracked changes. You should upload this as a separate file labeled 'Manuscript'.

If you would like to make changes to your financial disclosure, competing interests statement, or data availability statement, please make these updates within the submission form at the time of resubmission. Guidelines for resubmitting your figure files are available below the reviewer comments at the end of this letter

We look forward to receiving your revised manuscript.

Kind regards,

Hugues Richard

Guest Editor

PLOS Computational Biology

Shihua Zhang

Section Editor

PLOS Computational Biology

**Additional Editor Comments:**

Dear Authors,

Thank you for your submission. The reviewers appreciated the relevance of your work but have some remarks.

In particular the authors would like to address the novelty aspect of your work and perform a more proper and detailled benchmarking of the predicted interactions. Please address those points in an appropriate mannner

Best regards

**Journal Requirements:**

1) Please ensure that the CRediT author contributions listed for every co-author are completed accurately and in full.

At this stage, the following Authors/Authors require contributions: Miguel Fernandez-Martin, Camila Pontes, Victoria Ruiz-Serra, and Alfonso Valencia. Please ensure that the full contributions of each author are acknowledged in the "Add/Edit/Remove Authors" section of our submission form.

The list of CRediT author contributions may be found here: https://journals.plos.org/ploscompbiol/s/authorship#loc-author-contributions

2) We ask that a manuscript source file is provided at Revision. Please upload your manuscript file as a .doc, .docx, .rtf or .tex. If you are providing a .tex file, please upload it under the item type u2018LaTeX Source Fileu2019 and leave your .pdf version as the item type u2018Manuscriptu2019.

3) Please provide an Author Summary. This should appear in your manuscript between the Abstract (if applicable) and the Introduction, and should be 150-200 words long. The aim should be to make your findings accessible to a wide audience that includes both scientists and non-scientists. Sample summaries can be found on our website under Submission Guidelines:

https://journals.plos.org/ploscompbiol/s/submission-guidelines#loc-parts-of-a-submission

4) Please upload all main figures as separate Figure files in .tif or .eps format. For more information about how to convert and format your figure files please see our guidelines:

https://journals.plos.org/ploscompbiol/s/figures

5) We have noticed that you have uploaded Supporting Information files, but you have not included a list of legends. Please add a full list of legends for your Supporting Information files after the references list.

6) Please ensure that the funders and grant numbers match between the Financial Disclosure field and the Funding Information tab in your submission form. Note that the funders must be provided in the same order in both places as well.

- State the initials, alongside each funding source, of each author to receive each grant. For example: "This work was supported by the National Institutes of Health (####### to AM; ###### to CJ) and the National Science Foundation (###### to AM)."

- State what role the funders took in the study. If the funders had no role in your study, please state: "The funders had no role in study design, data collection and analysis, decision to publish, or preparation of the manuscript.".

If you did not receive any funding for this study, please simply state: u201cThe authors received no specific funding for this work.u201d

**Reviewers' comments:**

Reviewer's Responses to Questions

**Comments to the Authors:**

**Please note here if the review is uploaded as an attachment.**

Reviewer #1: In this paper the authors describe an updated version of their tool ContextMirror to version 2.0 which predicts PPI-interactions. They apply it to three bacterial genomes and identify a number of previously unannotated PPI-networks.

The method is based on phylogenetic similarity of potentially interacting proteins across a number of specied.

What is severely missing in this paper is a proper benchmarking study. The authors use a positive set of proteins from : IntAct, STRING (score >0.2), EcoCyc and Interactome3D. Here it is unclear how STRING is used, as it is both a collection of experimental information and a prediction of interacting proteins using various method (including methods that are not that different from ContectMirror), so it can be asked if this is a suitable set of positive interactions. From what I understand all pairs not present in these databases are assumed to be negative - although this is certainly now true - it is likely an OK assumption. However the authors to need to answer how does the performance vary with a different set of positives. There are also large scale PPI studies (yeast2hybrid, tap-tag etc) primarily for yeast that can be used to benchmark the methods,

Once a good set of "true" interactions are made - the authors do need to compare their method with state of the art methods, for instance RoseTTAfold-PPI is fast enough to run on all pairs, but you could also consider STRING (or Funcoup) as alternative predictors. There are also other methods (which perform badly in general) such as D-script that could be included.

Another question is if this method is useful beyond bacterial genomes. How does it apply to yeast, which is not that much bigger so it should be possible to run it. I would assume that the existence of paralogs might make it less accurate though.

It should also be noted that the performance is actually really bad (10% recall at 30% precision). Perhaps the precision can be improved by using AlphaFold2 (or 3) to predict the interactions - this should filter away some FPs, I think.

I looked at the code and it looks OK (but it is not good practice to include _pycache__ and other temporary directoris in the git repository, so a small cleanup would be good.

Reviewer #2: The author introduces ContextMirror 2.0 (CM2.0), a co-evolution analysis framework that is entirely sequence-dependent, unsupervised, and capable of large-scale implementation for predicting protein-protein interaction at the species level. This framework was used to compare conserved and species-specific interactions among three bacteria: E. coli, S. enterica, and S. aureus.

1. The current paper's format has significant room for improvement. Lines 39-67 focus solely on discussing the CM2.0 tool. In accordance with standard academic writing format, the introduction should address why this research is being conducted, the challenges present in the current field, an overview of related work, and a discussion of related work. These elements are currently missing from the introduction and should be rewritten. It is recommended that the author categorize related work by introducing traditional predictive PPI algorithms and recent developments, such as 10.1109/TCBB.2022.3196336 and 10.1109/JBHI.2024.3357979.

2. The author mentions that, for a given genome, the top 1000 predictions achieve approximately 40% precision. I would like to understand what this number implies compared to other tools. How can the remaining 60% of PPI be validated, considering that these predictions are made but not found in the database?

3. I am curious whether the author has evaluated the runtime of CM2 or compared it with similar tools, as this tool involves extensive phylogenetic tree computations, such as sequence alignment and profile alignment, which are relatively time-consuming calculations. I only saw the time of CM2 in Table S7.

4. The author should address in Section discussion the tool's performance in certain protein families where public databases lack a sufficient number of shared orthologs, making it unable to meet the threshold of "N common species."

5. Regarding future work, please discuss the possibility of applying advanced graph neural networks (10.1109/JBHI.2024.3357979), that are developed to learn sequence embeddings, for improved performance of PPI prediction.

Reviewer #3: Summary:

The authors introduce a new method, ContextMirror2.0. It predicts the protein-protein interactions (PPIs) of a query proteome by quantifying the co-evolution of protein pairs. The approach evaluates all possible protein pairs (p, q), provided both proteins have a sufficient number of orthologs in other species. Co-evolution is quantified by correlating the similarity of MSA-derived phylogenetic trees. The resulting score is adjusted by accounting for the fact that proteins do not interact in isolation; thus, the co-evolution of a pair should also be reflected in third proteins. Protein pairs are then ranked based on this score, and those above a certain threshold are predicted to interact, either physically or functionally.

The authors demonstrate the usefulness of their method by predicting all PPIs for E. coli, S. enterica (as well-characterized, Gram-negative examples), and S. aureus (as a poorly characterized, Gram-positive example). By modeling their best predictions with AlphaFold Multimer, they show that their method mostly predicts non-direct interactions. They further merged the predicted networks of the three species by identifying close homologs to identify functional communities preserved across species. Within these communities, the method enables hypothesis generation about the function and divergence of uncharacterized proteins.

The strengths of the method are that it does not need (the sparsely available) negative examples, that it is interpretable, rather fast (compared to deep learning approaches), and enables network community detection for functional analysis. The drawbacks of the method are that it is probably prone to false positives and does not distinguish between physical and non-physical, functional interactions (which could, depending on the application, also be an advantage). Overall, the research is novel, of high quality, and useful to the field. I strongly encourage publication after addressing the following points:

Major Points:

1. Methods: The methods section is at times difficult to follow, particularly regarding which steps are performed per protein and which are performed per protein pair. This could be greatly improved by including pseudocode or using mathematical notation. For example:

(1) For each protein p, we identify a set of orthologs O_p using RBH.

(2) For each possible protein pair (p,q) in the proteome of the query species, we construct O_{pq} = O_p \intersect O_q.

(3) If |O_{pq}| >= 1000, we calculate two MSEs MSE_p, MSE_q against all ortholog sequences of O_{pq}

… and so on. Please consider revising the methods accordingly, as this is central to the paper’s clarity.

2. Methods: Please also revise the figure of the ContextMirror2.0 pipeline (Fig. 7) for better clarity:

2.1. Change "Multi-FASTA protein" to, e.g., "Multi-FASTA file of the species proteome".

2.2. Show that the FASTA input consists of proteins A, B, C, D, possibly using color coding.

2.3. Make it visually clear that the next steps operate on pairs of proteins. Currently, it appears the steps are for a single protein. Highlight, for instance, that you’re working with (A, D) in subsequent panels.

2.4. Indicate explicitly that each dot in the tree-tree correlation plots corresponds to a specific protein pair, and annotate accordingly (e.g., “(A, D)”).

2.5. Label matrices with their names (e.g., r for the tree similarity matrix) for easier reference.

3. Methods/Figure 1: I am unsure about the calculation of the random precision, more precisely, about the denominator. You currently calculate the probability of obtaining the TPs by chance among all possible edges. However, your method filters out edges that lack sufficient ortholog coverage (>1000). Shouldn’t the denominator only include pairs that meet this criterion? Please clarify or correct this if needed.

4. Methods: You state that your interaction score is bounded between 0 and 2. I don’t know if this is true. If n>6, -0.1+0.02(n-1) is >0, which makes the score grow with the number of third interactors. If we had a perfect correlation of 1 for PPcorr_{AB} and 100 perfectly correlated third interactors (+1/100*100=1), we would get 1+1-0.1+0.02*99=3.88. If they were perfectly negatively correlated, we would get -1-1-0.1+0.02*99=-0.12. Am I misunderstanding something here? If so, please make this clearer in the formula or surrounding text.

5. Results: Did you re-run the method from Cong et al. for your comparisons? Since their work dates back to 2019, rerunning it with current datasets could yield different results. Also, did you compare run times from their reported values or based on your own benchmarking setup? Please clarify and, if not already done, consider re-running Cong et al. for a fair comparison.

6. Results: You appear to model only single copies of each protein. However, many protein complexes include multiple copies of the same subunit. If I understand correctly, your approach could capture this. If so, this is an important capability and should be discussed in the manuscript, especially given that many PPIs form complexes involving multiple copies of the same proteins, cofactors ,or ligands.

7. Availability: I cannot access your GitHub, I think it might be private. Please change it to public.

Minor Points

1. Consider making Figure 7 your new Figure 1, as it outlines the core methodological contribution. You might also incorporate the interaction score formula in this figure to fully represent your pipeline visually.

2. Results 2.1: The sentence: “…CM2.0 tends to consistently assign high scores to true positive PPIs […] with an accuracy […] comparable to other similar methods” could be improved:

2.1. “tends to consistently” contradicts itself. I would suggest to strike the consistently as you only recover ~20% of the ground truth set with a precision of ~20%.

2.2. As you compare only to Cong et al., please revise “other similar methods” to “Cong et al.” for precision.

3. Results 2.3: The transition from “three electron transfer flavoproteins are predicted to interact with yibF and rfbI” to “this suggests host-pathogen interactions and oxidative stress in S. enterica” happens too abruptly for me. As a bioinformatician with not much knowledge of bacterial pathways, I found this reasoning hard to follow. Please provide additional context or references to help readers understand the biological rationale.

4. Methods: In your interaction score, you write PPcorr_{AB}, but before, you term this matrix r’. For consistency, please update to r’_{AB}. Also, you sum from i=1 to n, but i does not occur.

5. Methods: Why are there three different p-value cut-offs (10^-5 for r, 10^-11 for r’, 10^-6 for rho)? Why is only r’ corrected for multiple testing? Please justify the differences in thresholds and correction procedures, or adapt for consistency.

6. Supplement, Figure S2: The x-axis label “Min Nf” is unclear. Please explain this in the figure caption.

7. Methods: You currently refer to the interaction score as “IS,” which may carry unintended associations. Consider renaming it to something IntScore_{AB}? score_{AB}? CM2.0 score_{AB}?

Reviewer #5: The study introduces ContextMirror2.0 (CM2.0), a sequence-based coevolutionary method for predicting species-specific protein-protein interactions (PPIs), aiming to overcome limitations of previous computational and experimental approaches. Recent DL and ML tools have revolutionized PPIs prediction; however, predicting protein-protein or protein-ligand interactions without any prior knowledge remains a challenge for the scientific community. Achieving 40% precision in top predictions is considered strong for PPI prediction, as in realistic proteome-wide PPI datasets, interacting protein pairs are extremely rare compared to all possible pairs.

Overall, this is a clear, concise, and well-written manuscript. I find the research topic interesting and relevant to PLOS Comp Bio. The simulation results are easy to follow, and the manuscript mostly describes methods used, presented figures, and structural details. The results achieved are well-discussed in the main body of the reported manuscript. There are a few points that should be addressed:

When tested with AlphaFold, only 3 out of 30 high-scoring predicted interactions produced structurally acceptable models. Is there a reason for AlphaFold's failure other than no experimental data to predict these interactions, or does this suggest that high coevolutionary scores do not always indicate physically plausible complexes?

Line#141-142: “…3 out of 17 true positive interactions tested did surpass the pDockQ2 threshold.” This statement is not clear to me and needs elaboration on AF distinctions.

**********

**Have the authors made all data and (if applicable) computational code underlying the findings in their manuscript fully available?**

The PLOS Data policyrequires authors to make all data and code underlying the findings described in their manuscript fully available without restriction, with rare exception (please refer to the Data Availability Statement in the manuscript PDF file). The data and code should be provided as part of the manuscript or its supporting information, or deposited to a public repository. For example, in addition to summary statistics, the data points behind means, medians and variance measures should be available. If there are restrictions on publicly sharing data or code —e.g. participant privacy or use of data from a third party—those must be specified.

Reviewer #1: Yes

Reviewer #2: Yes

Reviewer #3: **No:**  I could not access the GitHub repository, the link was not working. I think it might still be set to private.

Reviewer #5: Yes

**********

PLOS authors have the option to publish the peer review history of their article (what does this mean?). If published, this will include your full peer review and any attached files.

If you choose “no”, your identity will remain anonymous but your review may still be made public.

**Do you want your identity to be public for this peer review?** For information about this choice, including consent withdrawal, please see our Privacy Policy.

Reviewer #1: **Yes:**  Arne Elofsson

Reviewer #2: No

Reviewer #3: No

Reviewer #5: No

[NOTE: If reviewer comments were submitted as an attachment file, they will be attached to this email and accessible via the submission site. Please log into your account, locate the manuscript record, and check for the action link "View Attachments". If this link does not appear, there are no attachment files.]

**Figure resubmission:**

While revising your submission, please upload your figure files to the Preflight Analysis and Conversion Engine (PACE) digital diagnostic tool, https://pacev2.apexcovantage.com/. PACE helps ensure that figures meet PLOS requirements. To use PACE, you must first register as a user. Registration is free. Then, login and navigate to the UPLOAD tab, where you will find detailed instructions on how to use the tool. If you encounter any issues or have any questions when using PACE, please email PLOS at figures@plos.org. Please note that Supporting Information files do not need this step. If there are other versions of figure files still present in your submission file inventory at resubmission, please replace them with the PACE-processed versions.

**Reproducibility:**

To enhance the reproducibility of your results, we recommend that authors of applicable studies deposit laboratory protocols in protocols.io, where a protocol can be assigned its own identifier (DOI) such that it can be cited independently in the future. Additionally, PLOS ONE offers an option to publish peer-reviewed clinical study protocols. Read more information on sharing protocols at https://plos.org/protocols?utm_medium=editorial-email&utm_source=authorletters&utm_campaign=protocols

# Author comment: Author response to Decision Letter 1

**DOI:** 10.1371/journal.pcbi.1014839.r002

7 Oct 2025

Attachment Submitted filename: Response to Reviewers.pdf

# Peer review: Decision Letter 1

**DOI:** 10.1371/journal.pcbi.1014839.r003

**Authors:** Shihua Zhang

3 Dec 2025

PCOMPBIOL-D-25-00705R1

Sequence-based coevolutionary prediction of species-specific interactomes

PLOS Computational Biology

Dear Dr. Fernandez-Martin,

Thank you for submitting your manuscript to PLOS Computational Biology. After careful consideration, we feel that it has merit but does not fully meet PLOS Computational Biology's publication criteria as it currently stands. Therefore, we invite you to submit a revised version of the manuscript that addresses the points raised during the review process.

Please submit your revised manuscript by Feb 02 2026 11:59PM. If you will need more time than this to complete your revisions, please reply to this message or contact the journal office at ploscompbiol@plos.org. When you're ready to submit your revision, log on to https://www.editorialmanager.com/pcompbiol/ and select the 'Submissions Needing Revision' folder to locate your manuscript file.

Please include the following items when submitting your revised manuscript:

* A rebuttal letter that responds to each point raised by the editor and reviewer(s). You should upload this letter as a separate file labeled 'Response to Reviewers'. This file does not need to include responses to formatting updates and technical items listed in the 'Journal Requirements' section below.

* A marked-up copy of your manuscript that highlights changes made to the original version. You should upload this as a separate file labeled 'Revised Manuscript with Track Changes'.

* An unmarked version of your revised paper without tracked changes. You should upload this as a separate file labeled 'Manuscript'.

If you would like to make changes to your financial disclosure, competing interests statement, or data availability statement, please make these updates within the submission form at the time of resubmission. Guidelines for resubmitting your figure files are available below the reviewer comments at the end of this letter.

We look forward to receiving your revised manuscript.

Kind regards,

Shihua Zhang

Section Editor

PLOS Computational Biology

Shihua Zhang

Section Editor

PLOS Computational Biology

**Additional Editor Comments:**

We suggest you address the concerns proposed by Reviewers 3 and 1. We will reevaluate your submission.

**Reviewers' comments:**

Reviewer's Responses to Questions

**Comments to the Authors:**

**Please note here if the review is uploaded as an attachment.**

Reviewer #1: I really have no comments as the authors basically did not do anything regarding my raised concerns, so I leave it up to the editor to evaluate if my concerns where important or not.

Reviewer #2: All of my concerns have been addressed.

Reviewer #3: Almost all of my comments were adequately addressed. The only comment that is still confusing to me is the one about the calculation of the random precision:

In your answer, you stated that “the denominator includes only the protein pairs that CM2.0 is able to evaluate (i.e., those with ≥1000 shared orthologs)”. However, in Supplementary Table S4, you calculate the random precision as true positives / potential PPIs, where the number of potential PPIs is calculated as [N*(N-1)]/2, N := number of proteins in the proteome of the species. I still think you need to adjust this to the number of protein pairs that ContextMirror 2.0 could potentially predict, which is the number of protein pairs that survive the filtering step. An alternative approach would be to draw a second line into the plot/make another panel in Figure 1, representing this form of random precision, so that readers can see how ContextMirror2.0 performs in comparison to the baseline, which simply predicts a PPI for all protein pairs that share at least 1000 orthologs.

Apart from that, I just have some small formal comments:

1. In the introduction, a sentence starting with “We benchmarked” is left unfinished.

2. There are some formatting artifacts from the markup, but maybe that was just the submission system. Examples are: “…are kept.-In these cases…”, “…where N M is the number of proteins…”, “…given a third protein (N k)…”, “…of proteins A p and B q…”, or the definition of the penalty term.

3. In the formula of r_{pq}, there is a ^2 missing in the first term of the denominator.

Reviewer #4: My recommendations were not included in the decision letter.

Reviewer #5: The authors have adequately addressed all of the revision requests. They have made significant improvements to the manuscript, and the revised version has been substantially strengthened. The current version meets the standards for publication and is suitable for acceptance.

Minor correction: Some of the equations are not displaying correctly, possibly due to file conversion issues. Please review the Methods section to ensure all equations appear properly.

**********

**Have the authors made all data and (if applicable) computational code underlying the findings in their manuscript fully available?**

The PLOS Data policyrequires authors to make all data and code underlying the findings described in their manuscript fully available without restriction, with rare exception (please refer to the Data Availability Statement in the manuscript PDF file). The data and code should be provided as part of the manuscript or its supporting information, or deposited to a public repository. For example, in addition to summary statistics, the data points behind means, medians and variance measures should be available. If there are restrictions on publicly sharing data or code —e.g. participant privacy or use of data from a third party—those must be specified.

Reviewer #1: Yes

Reviewer #2: None

Reviewer #3: Yes

Reviewer #4: None

Reviewer #5: Yes

**********

PLOS authors have the option to publish the peer review history of their article (what does this mean?). If published, this will include your full peer review and any attached files.

If you choose “no”, your identity will remain anonymous but your review may still be made public.

**Do you want your identity to be public for this peer review?** For information about this choice, including consent withdrawal, please see our Privacy Policy.

Reviewer #1: **Yes:**  arne

Reviewer #2: No

Reviewer #3: No

Reviewer #4: No

Reviewer #5: No

[NOTE: If reviewer comments were submitted as an attachment file, they will be attached to this email and accessible via the submission site. Please log into your account, locate the manuscript record, and check for the action link "View Attachments". If this link does not appear, there are no attachment files.]

**Figure resubmission:**

While revising your submission, we strongly recommend that you use PLOS’s NAAS tool (https://ngplosjournals.pagemajik.ai/artanalysis) to test your figure files. NAAS can convert your figure files to the TIFF file type and meet basic requirements (such as print size, resolution), or provide you with a report on issues that do not meet our requirements and that NAAS cannot fix.

After uploading your figures to PLOS’s NAAS tool - https://ngplosjournals.pagemajik.ai/artanalysis, NAAS will process the files provided and display the results in the "Uploaded Files" section of the page as the processing is complete. If the uploaded figures meet our requirements (or NAAS is able to fix the files to meet our requirements), the figure will be marked as "fixed" above. If NAAS is unable to fix the files, a red "failed" label will appear above. When NAAS has confirmed that the figure files meet our requirements, please download the file via the download option, and include these NAAS processed figure files when submitting your revised manuscript.

**Reproducibility:**

To enhance the reproducibility of your results, we recommend that authors of applicable studies deposit laboratory protocols in protocols.io, where a protocol can be assigned its own identifier (DOI) such that it can be cited independently in the future. Additionally, PLOS ONE offers an option to publish peer-reviewed clinical study protocols. Read more information on sharing protocols at https://plos.org/protocols?utm_medium=editorial-email&utm_source=authorletters&utm_campaign=protocols

# Author comment: Author response to Decision Letter 2

**DOI:** 10.1371/journal.pcbi.1014839.r004

21 Jan 2026

Attachment Submitted filename: Response_to_Reviewers_auresp_2.pdf

# Peer review: Decision Letter 2

**DOI:** 10.1371/journal.pcbi.1014839.r005

**Authors:** Shihua Zhang, Hugues Richard

8 Apr 2026

PCOMPBIOL-D-25-00705R2

Sequence-based coevolutionary prediction of species-specific interactomes

PLOS Computational Biology

Dear Dr. Fernandez-Martin,

Thank you for submitting your manuscript to PLOS Computational Biology. After careful consideration, we feel that it has merit but does not fully meet PLOS Computational Biology's publication criteria as it currently stands. Therefore, we invite you to submit a revised version of the manuscript that addresses the points raised during the review process.

Please submit your revised manuscript by Jun 08 2026 11:59PM. If you will need more time than this to complete your revisions, please reply to this message or contact the journal office at ploscompbiol@plos.org. When you're ready to submit your revision, log on to https://www.editorialmanager.com/pcompbiol/ and select the 'Submissions Needing Revision' folder to locate your manuscript file.

Please include the following items when submitting your revised manuscript:

* A letter that responds to each point raised by the editor and reviewer(s). You should upload this letter as a separate file labeled 'Response to Reviewers'. This file does not need to include responses to formatting updates and technical items listed in the 'Journal Requirements' section below.

* A marked-up copy of your manuscript that highlights changes made to the original version. You should upload this as a separate file labeled 'Revised Manuscript with Track Changes'.

* An unmarked version of your revised paper without tracked changes. You should upload this as a separate file labeled 'Manuscript'.

If you would like to make changes to your financial disclosure, competing interests statement, or data availability statement, please make these updates within the submission form at the time of resubmission. Guidelines for resubmitting your figure files are available below the reviewer comments at the end of this letter

We look forward to receiving your revised manuscript.

Kind regards,

Hugues Richard

Guest Editor

PLOS Computational Biology

Shihua Zhang

Section Editor

PLOS Computational Biology

**Additional Editor Comments:**

Dear Authors,

Thank you for sending the revision for this paper. Because of some remaining differences in the reviews in the last submission, I decided to ask two additional reviewers for their opinion. You will find their reviews below.

While both reviews are globally positive, some points still need to be addressed to ensure that the paper is suitable for publication. In particular the evaluation of the results against the baseline must be improved, by providing more comparison than the simple random baseline with generalist tools such as D-Script or RoseTTAFold-PPI. Additionally, Reviewer 7 also noted that operons and syntenic cluster could play a role as a confounder, and this should be integrated in the validation, either as a baseline or as an effect that can be ruled out.

**Reviewers' comments:**

Reviewer's Responses to Questions

**Comments to the Authors:**

**Please note here if the review is uploaded as an attachment.**

Reviewer #6: The manuscript "Sequence-based coevolutionary prediction of species-specific interactomes" reports on a phylogeny-based method for detecting pairs of proteins with strong coevolution signal suggesting functional and/or physical interaction. The method is applicable at large scale. The authors report predictive performance and enrichment compared to random baseline and showcase how it can be used to get new biological insights about functional communities.

The title is somewhat an over-statement since the method was applied and validated only in bacteria and the authors recognised in the first revision that it would require adaptation to apply it to eukaryotic species, in particular for disentangling paralogs. These aspects should be made explicit in the manuscript.

"For instance, although a S. aureus PPI received a high CM2.0 interaction score and was validated in experimental databases, the AF-predicted complex had a very low pDockQ2, suggesting that the proteins are functionally related but do not form a stable 375 physical interaction. " I would recommend to nuance the statement here. AlphaFold is unable to predict even certain complexes that are experimentally resolved with high resolution and extended interface.

Quantitative comparisons are reported only against a random baseline. While I understand that there are few currently available methods that address exactly the same question as CM2.0 and that it is not so easy to reproduce or update with more recent sequence data the work of Cong et al. 2019, the reader is left unsure about the positioning of the present work with respect to SOTA in PPI prediction (or even prioritization). The lack of predictive performance or even runtime comparisons with RoseTTAFold-PPI or D-Script for instance is not satisfactorily justified. If CM2.0 is faster, then the authors should quantify how much. If CM2.0 has lower predicion or resolution, then the authors should (even roughly) estimate by how much, and discuss the tradeoff between predictive performance on one side, and speed, interpretability and lack of supervision on the other side. The argument of defining an alternative or niche task for which the only available tool is CM2.0 is a bit light.

Reviewer #7: I think that the presented method is worth being published; however, the current state of the paper doesn't do it justice. The paper could greatly benefit from some editions to make it more self-contained and easier to read. Some clarifications are needed, and some extra tests could be valuable to ensure a proper evaluation of the presented method.

A. Method evaluation.

1. The CM2.0 method is presented as an improvement over a previous version, ContextMirror. However, the paper does not compare them directly, so it is difficult to address whether there is an improvement in the quality of the protein-protein interaction predictions. I guess based on some phrases, such as the one indicating that this is “a new high-throughput version of the ContextMirror method” that running the original method at the genome level is unfeasible. However, I imagine that some comparisons can still be done at the community level. This idea of picking a community can also be useful for comparing against more expensive methods, such as the ones predicting structures of the complexes (e.g., AF3) and the method from Cong et al. (2019). In such cases, picking a community defined from the ground truth PPI graph could be a better approach than picking one resolved by CM2.0.

2. Figure 1 shows the “precision values that would be expected by chance”, which are calculated as the number of true positives over the potential PPIs after ortholog filtering, as stated in Table S4 and in the methods section. However, a reader of the paper needs to go to those places to know that. It would have been better to state that earlier in the manuscript. In any case, such a value is “too random” to be the only metric to compare CM2.0 against. I would propose that a better comparison could have been to measure the precision and recall of inferring functional interactions using operons as communities. An idea that it is qualitatively referenced in the paper, but there is no quantitative comparison between the approaches. Furthermore, most of the genes in the communities shown in Figure 5 belong to operons. I can see that CM2.0 can also capture relations among operons, but still, it would be the most sensible baseline. Another simple metric that could also be evaluated as a baseline is the number of shared putative-interacting ortholog sequences in the MSA. It would be nice to see the precision and recall curves of those baselines and to compare the AUC values.

3. In the section where AlphaFold-Multimer is used to assess selected predicted PPIs, the authors rely on pDockQ2 as the main confidence metric. I would suggest that the authors use ipTM or the combined AlphaFold-Multimer ranking confidence score (0.8·ipTM + 0.2·pTM) instead or as a complement. This would make the analysis more consistent with standard practice and would provide a more appropriate assessment of whether high-scoring CM2.0 pairs also tend to yield confident complex models. Furthermore, it would be great to compare ipTM (and the combined score) and the IntScore using a continuous metric, such as the Pearson correlation coefficient.

4. Can CM2.0 be applied to non-bacterial genomes? Since the method was tested only on bacterial species, it is difficult to determine from the manuscript whether it generalizes beyond bacteria. If it is intended only for bacterial genomes, this limitation should be stated clearly. If not, including an eukaryotic example, ideally *H. sapiens* because of the abundance and quality of available PPI annotations, would strengthen the paper.

B. Other comments and questions.

Most of the style problems and missing details I have found came from the fact that the methods section is at the end. The methods section is well written and detailed; however, a reader that tackles the paper linearly is unable to fully understand the results. I would recommend introducing some method details early to ease readers tackling the paper linearly.

1. I have found the abstract to be a little cryptic, as “coevolutionary approaches” can mean many things; I will emphasize in the abstract that CM2.0 is measuring coevolution at the phylogenetic level, similarly to MirrorTree.

2. In the introduction, in the third paragraph, I would also acknowledge the current PPI models in AFDB and works such as Todor et al., “Predicting the protein interaction landscape of a free-living bacterium with pooled-AlphaFold3” in Molecular Systems Biology (2026). The field advances so quickly that this paragraph needs an update.

3. In the fourth introduction paragraph, I would add a longer description of the previous method, ContextMirror (and make more clear what CM 2.0 introduces). As mentioned before, coevolution can be measured among different entities, and the reader needs to know it as early as possible when reading the paper which one is being used here.

4. Can you really find species-specific interactions? While comparing species can highlight species-specific interactions, the level of resolution may be limited by the fact that at least 1,000 common species are needed when searching for orthologs. Therefore, it is possible that the method is actually detecting interactions that are specific at the genus or family level. However, truly species-specific interactions could also arise in contexts without that degree of phylogenetic conservation, and the method may fail to detect them. This is something that needs to be clarified or tested, as the current title of the article seems to suggest that the method identifies species-specific interactions.

5. In the first paragraph of the Results section, the authors note that strongly correlated protein pairs tend to be influenced by many third proteins. I wonder to what extent this trend may be expected from the topology of protein-protein interaction networks themselves, which are often described as having a small-world, modular, and hub-rich architecture. In such networks, proteins belonging to the same functional neighborhoods or dense communities would naturally be associated with many indirect contextual influences. Therefore, this pattern may reflect not only the specific biological signal captured by CM2.0 but also a property of PPI network organization. I do not necessarily see this as a flaw of the method, but I think it would be worth commenting on it explicitly in the manuscript.

6. You have tested different IntScore thresholds (1.6, 1.7 and 1.8) but you have a ground truth data to compare with. How can users without such data set a proper IntScore value?

7. When creating structural models, the authors tested whether these PPIs are present in the PDB and found that they are not. However, some of them may still have homologous interactions represented in the PDB, including entries deposited before the AlphaFold-Multimer training-data cutoff. If you want, you can use Foldseek-Multimer to find similar interfaces on the PDB.

8. A Venn diagram is the most effective way to represent Figure 3. If it doesn’t work, default to a bar chart, as it is more appropriate than a pie chart.

9. The last paragraph of page 8 does not provide a clear assessment of what fraction of these ‘potentially novel PPIs’ are likely to correspond to true functional interactions. If I understand correctly, these are interactions predicted by CM2.0 that are absent from the PPI databases used as ground truth. Therefore, based on the current analysis alone, they are more accurately described as false positives rather than as novel PPIs. Of course, some may indeed be genuine unannotated interactions, but the manuscript does not establish the likelihood of that scenario. Could transcriptomic data can give another layer of evidence to assess those cases? Perhaps those novel PPIs are due to functional rather than to physical association; therefore, better captured by transcriptomic associations than by conventional PPI annotations in databases.

10. When reading the paper, I often wondered to what extent your results could be confounded by operon organization and synteny, especially in Section 2.3, since most of the interactions shown in Figure 5 involve proteins encoded in the same operon. To account for synteny, it could be useful to include intergenic distance as an additional baseline, since it may serve as a proxy for both factors. However, I would appreciate it if you could find another way to better control or test for synteny.

11. Because this method is applied to bacterial genomes, where horizontal gene transfer can be common, I think the manuscript should explicitly acknowledge that non-tree-like evolutionary histories lie outside the scope of the presented method, if I understood correctly.

12. Does ABD30300.1 occupy a network environment similar to that of fakA? More generally, to what extent can similarity in network topology be used to transfer functional annotations across species?

13. In many places, correlation coefficients are filtered using p-value cutoffs. However, it is not always clear at which steps FDR or Bonferroni corrections are applied. As far as I understand, a Bonferroni correction is explicitly used for the profile-profile correlation step but not clearly for the other filtering steps. If multiple-testing correction is not applied throughout, would the results improve, or at least become more statistically consistent, by considering FDR or another multiple-testing correction at each filtering step?

14. It was not super clear to me how partial correlations are used to determine the number of third proteins influencing a given correlation. I would have expected this to be done, for example, by comparing the observed correlation with the corresponding partial correlations and then identifying how many third proteins produce a meaningful deviation. However, the paper only states that this is done ‘by setting a correlation cut-off,’ without clearly explaining how that cutoff is defined or applied.

15. Selecting random seed 76 because it optimizes precision for shared predictions raises a concern about selection bias in the illustrative examples. Although the main quantitative results are averaged across 100 runs, the manuscript should clarify whether the communities shown in Figures 5, 6, and S5–S11 are representative of typical runs or correspond to a best-case scenario.

16. Are the modifications mentioned in the last paragraph of Section 4.3 the only ones from ContextMirror to CM2.0? This should be briefly mentioned or described previously in the manuscript.

C. Text.

1. The abstract phrase starting with “While around 40%” is too long and difficult to understand.

2. The manuscript reports p-values as 0.0. Since p-values are not exactly zero, these should be reported as inequalities or in scientific notation.

3. In page 7, there is a mention of an “interaction score (IS)”; is that the same as the IntScore described in Methods? Note that “IS” is also used in other parts of the manuscript.

4. The paragraph beginning with ‘Using the same curated datasets’ in the Discussion section presents results that would be more appropriately placed in the Results section.

5. The last paragraph of Section 4.2 would be better placed at the figure description in the Results section.

**********

**Have the authors made all data and (if applicable) computational code underlying the findings in their manuscript fully available?**

The PLOS Data policyrequires authors to make all data and code underlying the findings described in their manuscript fully available without restriction, with rare exception (please refer to the Data Availability Statement in the manuscript PDF file). The data and code should be provided as part of the manuscript or its supporting information, or deposited to a public repository. For example, in addition to summary statistics, the data points behind means, medians and variance measures should be available. If there are restrictions on publicly sharing data or code —e.g. participant privacy or use of data from a third party—those must be specified.

Reviewer #6: None

Reviewer #7: Yes

**********

PLOS authors have the option to publish the peer review history of their article (what does this mean?). If published, this will include your full peer review and any attached files.

If you choose “no”, your identity will remain anonymous but your review may still be made public.

**Do you want your identity to be public for this peer review?** For information about this choice, including consent withdrawal, please see our Privacy Policy.

Reviewer #6: No

Reviewer #7: No

[NOTE: If reviewer comments were submitted as an attachment file, they will be attached to this email and accessible via the submission site. Please log into your account, locate the manuscript record, and check for the action link "View Attachments". If this link does not appear, there are no attachment files.]

**Figure resubmission:**

While revising your submission, we strongly recommend that you use PLOS’s NAAS tool (https://ngplosjournals.pagemajik.ai/artanalysis) to test your figure files. NAAS can convert your figure files to the TIFF file type and meet basic requirements (such as print size, resolution), or provide you with a report on issues that do not meet our requirements and that NAAS cannot fix.

After uploading your figures to PLOS’s NAAS tool - https://ngplosjournals.pagemajik.ai/artanalysis, NAAS will process the files provided and display the results in the "Uploaded Files" section of the page as the processing is complete. If the uploaded figures meet our requirements (or NAAS is able to fix the files to meet our requirements), the figure will be marked as "fixed" above. If NAAS is unable to fix the files, a red "failed" label will appear above. When NAAS has confirmed that the figure files meet our requirements, please download the file via the download option, and include these NAAS processed figure files when submitting your revised manuscript.

**Reproducibility:**

To enhance the reproducibility of your results, we recommend that authors of applicable studies deposit laboratory protocols in protocols.io, where a protocol can be assigned its own identifier (DOI) such that it can be cited independently in the future. Additionally, PLOS ONE offers an option to publish peer-reviewed clinical study protocols. Read more information on sharing protocols at https://plos.org/protocols?utm_medium=editorial-email&utm_source=authorletters&utm_campaign=protocols

# Author comment: Author response to Decision Letter 3

**DOI:** 10.1371/journal.pcbi.1014839.r006

2 Aug 2026

Attachment Submitted filename: Response_to_Reviewers_auresp_3.pdf

# Peer review: Decision Letter 3

**DOI:** 10.1371/journal.pcbi.1014839.r007

**Authors:** Shihua Zhang, Hugues Richard

21 Sep 2026

Dear Mr. Fernandez-Martin,

We are pleased to inform you that your manuscript 'Sequence-based coevolutionary prediction of species-specific interactomes' has been provisionally accepted for publication in PLOS Computational Biology.

Before your manuscript can be formally accepted you will need to complete some formatting changes, which you will receive in a follow up email. A member of our team will be in touch with a set of requests.

Please note that your manuscript will not be scheduled for publication until you have made the required changes, so a swift response is appreciated.

IMPORTANT: The editorial review process is now complete. PLOS will only permit corrections to spelling, formatting or significant scientific errors from this point onwards. Requests for major changes, or any which affect the scientific understanding of your work, will cause delays to the publication date of your manuscript.

Should you, your institution's press office or the journal office choose to press release your paper, you will automatically be opted out of early publication. We ask that you notify us now if you or your institution is planning to press release the article. All press must be co-ordinated with PLOS.

Thank you again for supporting Open Access publishing; we are looking forward to publishing your work in PLOS Computational Biology.

Best regards,

Hugues Richard

Guest Editor

PLOS Computational Biology

Shihua Zhang

Section Editor

PLOS Computational Biology

***********************************************************

Dear Mr Fernandez-Martin,

Thank you for submitting your revision, the main remarks from the reviewers have been substantially addressed and I am happy to share with you that the manuscript is now suitable for publication.

Please address any remaining remarks before submitting the manuscript.

Reviewer's Responses to Questions

**Comments to the Authors:**

**Please note here if the review is uploaded as an attachment.**

Reviewer #6: The authors have substantially improved the work and the manuscript.

Reviewer #7: I am glad to see how the author has taken the reviewers' comments into consideration, making the publication much stronger than it was before. There are only a few details, especially the newly added modifications that need some extra attention or discussion.

1. You have defined IntScore = 0 for the cases in which the protein pair doesn't meet the filtering criteria. We can see in the table that AUC values decreased because of that. However, it could have been nice to have a measure of the false positives we have in each dataset when we use IntScore = 0. In other words, to measure how many protein pairs we are losing because there is not enough information to use CM2.0.

2. I understand that CM2.0 infers coevolution by comparing the evolutionary histories of homologous protein families. In this sense, although the predictions are made for a particular target proteome, the evidence supporting each prediction necessarily comes from homologues distributed across multiple species. I therefore wonder whether “species-specific” can be deleted from the title and used in the article where it can be clarified. The current wording might suggest that the interaction is inferred from information specific to a single species, whereas the coevolutionary signal is inherently derived from a broader phylogenetic context.

3. Related to the previous point, it would be useful to better characterize how much phylogenetic information is required for CM2.0 to produce a reliable prediction. I understand that a threshold of 1,000 sequences is used by default. However, the raw number of sequences or taxa may not be sufficient to describe the information content of the corresponding phylogeny: 1,000 very closely related sequences are unlikely to provide the same information as 1,000 sequences spanning a broader evolutionary range. Could the authors discuss whether other quantities—such as the effective number of sequences, phylogenetic diversity, total tree length, or the distribution of pairwise evolutionary distances—could provide a more informative criterion? It would also be interesting to know whether there is an empirically identifiable range of phylogenetic diversity over which CM2.0 performs reliably, rather than simply a minimum number of sequences.

4. Rather than discarding all protein pairs with fewer than 1,000 orthologs, could the authors consider assessing more directly whether a given protein pair contains sufficient information for a reliable prediction? A conventional power calculation for the Pearson correlation would not be straightforward here, because the observations are not statistically independent but are related through the underlying phylogeny. I wonder whether this dependence also has implications for the calculation and interpretation of the correlation coefficients used to derive IntScore itself. Nevertheless, an empirical approach might be possible. For example, the authors could repeatedly subsample taxa and assess the stability of the correlation or final IntScore as a function of both the number of orthologs and their phylogenetic diversity. This could provide a more informative criterion than a fixed cutoff of 1,000 orthologs. In particular, identifying cases in which there is genuinely insufficient phylogenetic information could help mitigate the loss of performance caused by assigning IntScore = 0 to all pairs that fail the current filtering criterion.

5. I am not sure I understand the interpretation of the partial correlations in Step 3. As I understand it, if controlling for a third protein substantially reduces the correlation between the coevolutionary profiles of two proteins, then that third protein could be considered to explain or influence part of their original association. Conversely, if the partial correlation remains large and statistically significant after controlling for the third protein, this would indicate that the original association persists and that the third protein does not explain much of it. Therefore, the statement that statistically significant partial correlations identify third proteins that “influence” the correlation appears to be reversed. Could the authors clarify the rationale and, if necessary, correct the interpretation?

**********

**Have the authors made all data and (if applicable) computational code underlying the findings in their manuscript fully available?**

The PLOS Data policyrequires authors to make all data and code underlying the findings described in their manuscript fully available without restriction, with rare exception (please refer to the Data Availability Statement in the manuscript PDF file). The data and code should be provided as part of the manuscript or its supporting information, or deposited to a public repository. For example, in addition to summary statistics, the data points behind means, medians and variance measures should be available. If there are restrictions on publicly sharing data or code —e.g. participant privacy or use of data from a third party—those must be specified.

Reviewer #6: None

Reviewer #7: Yes

**********

PLOS authors have the option to publish the peer review history of their article (what does this mean?). If published, this will include your full peer review and any attached files.

If you choose “no”, your identity will remain anonymous but your review may still be made public.

**Do you want your identity to be public for this peer review?** For information about this choice, including consent withdrawal, please see our Privacy Policy.

Reviewer #6: No

Reviewer #7: No

# Editor report: Acceptance letter

**DOI:** 10.1371/journal.pcbi.1014839.r008

**Authors:** Shihua Zhang, Hugues Richard

PCOMPBIOL-D-25-00705R3

Sequence-based coevolutionary prediction of species-specific interactomes

Dear Dr Fernandez-Martin,

I am pleased to inform you that your manuscript has been formally accepted for publication in PLOS Computational Biology. Your manuscript is now with our production department and you will be notified of the publication date in due course.

The corresponding author will soon be receiving a typeset proof for review, to ensure errors have not been introduced during production. Please review the PDF proof of your manuscript carefully, as this is the last chance to correct any errors. Please note that major changes, or those which affect the scientific understanding of the work, will likely cause delays to the publication date of your manuscript.

Soon after your final files are uploaded, unless you have opted out, the early version of your manuscript will be published online. The date of the early version will be your article's publication date. The final article will be published to the same URL, and all versions of the paper will be accessible to readers.

For Research, Software, and Methods articles, you will receive an invoice from PLOS for your publication fee after your manuscript has reached the completed accept phase. If you receive an email requesting payment before acceptance or for any other service, this may be a phishing scheme. Learn how to identify phishing emails and protect your accounts at https://explore.plos.org/phishing.

Thank you again for supporting PLOS Computational Biology and open-access publishing. We are looking forward to publishing your work!

With kind regards,

Sharmila Kamatchi

PLOS Computational Biology | Carlyle House, Carlyle Road, Cambridge CB4 3DN | United Kingdom ploscompbiol@plos.org | Phone +44 (0) 1223-442824 | ploscompbiol.org | @PLOSCompBiol
